# Supplementary material for: The Transcriptional Regulator SpxA1 Influences the Morphology and Virulence of Listeria monocytogenes
Source: Infect Immun. 2022 Sep 14;90(10):e00211-22. doi: 10.1128/iai.00211-22 (PMC9584327; doi:10.1128/iai.00211-22)
Supplement: Supplemental file 1 — Fig. S1 to S4 and Tables S1 to S4. Download iai.00211-22-s0001.pdf, PDF file, 4.2 MB [file iai.00211-22-s0001.pdf]

SUPPLEMENTAL MATERIAL

The transcriptional regulator SpxA1 influences the morphology and virulence of *Listeria monocytogenes*

Monica R. Cesinger, Oluwasegun I. Daramola, Lucy M. Kwiatkowski, and Michelle L. Reniere

SUPPLEMENTAL FIGURES

Figure S1. Anaerobic growth kinetics of *L. monocytogenes* wt and Δ*spxA1*. Anerobic cultures were diluted to an OD_{600} of 0.02 in BHI and grown anaerobically. Every two hours, bacterial cultures were serially diluted and plated to enumerate CFU. Data are the means and SEM of three biological replicates. The differences between wt and Δ*spxA1* were not statistically different at any time point, as determined by unpaired *t*-test.

Figure S2. SpxA1-regulated redox homeostasis genes do not influence elongation. Bacteria were grown anaerobically in rich broth and cell perimeters were measured from phase contrast images using Celltool. The dotted line represents the wt mean, and solid black lines indicate the means of each strain.

Figure S3. Co-culture with wt does not rescue Δ*spxA1* filamentation. *L. monocytogenes* wt constitutively producing GFP was co-cultured with Δ*spxA1* constitutively producing mCherry for 2 hours. (A) A representative image of a 2:1 (wt: Δ*spxA1*) co-culture is shown. (B) Cell perimeters of each condition were measured with Celltool and the data shown for the co-cultures indicate Δ*spxA1* cell perimeters. Data are from a single representative replicate where at least 1,000 cells or ten fields of view were quantified. The lines represent the means.

Figure S4. Motility of *L. monocytogenes* incubated anaerobically. BHI 0.4% agar motility plates were inoculated via stab with bacteria and incubated anaerobically at either room temperature or 37ºC for 4 days. The diameter of bacterial migration from the central stab was measured and compared to

that of wt at room temperature. Data are from 4 or 6 biological replicates. The diameter of the non-motile Δ*flaA* mutant migration represents the diameter of the inoculation stab. Bars represent means and SEMs, and significance was determined by unpaired *t*-test. *** $p < 0.001$; n.s. $p > 0.05$.

SUPPLEMENTAL TABLES

Table S1. Proteins decreased in abundance in Δ*spxA1* compared to wt

Table S2. Proteins increased in abundance in Δ*spxA1* compared to wt

Table S3. Complete list of proteins detected by whole cell proteomics

Table S4. Strains used in this study

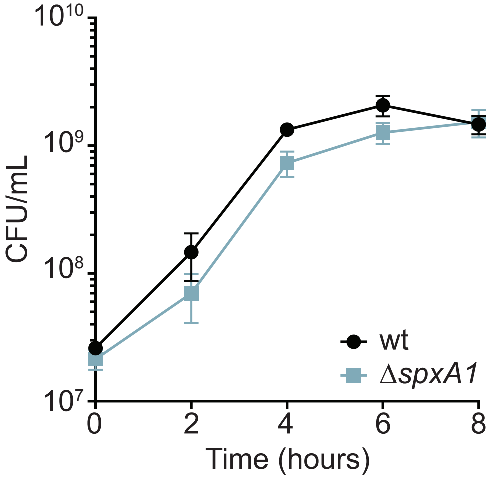

CFU/mL
10^10
10^9
10^8
10^7
0
2
4
6
8
Time (hours)
wt
ΔspxA1

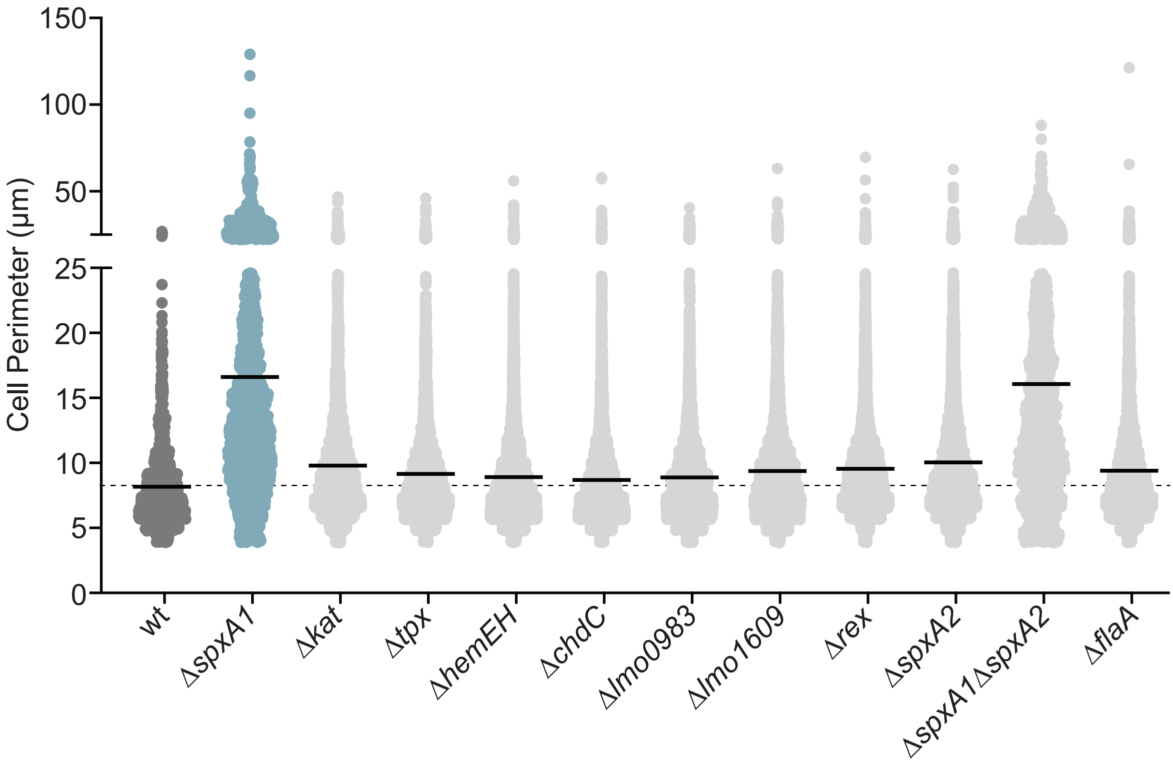
Cell Perimeter (µm)
150
100
50
25
20
15
10
5
0
wt
ΔspxA1
Δkat
Δtpx
ΔhemEH
ΔchdC
Δlmo0983
Δlmo1609
Δrex
ΔspxA2
ΔspxA1ΔspxA2
ΔflaA

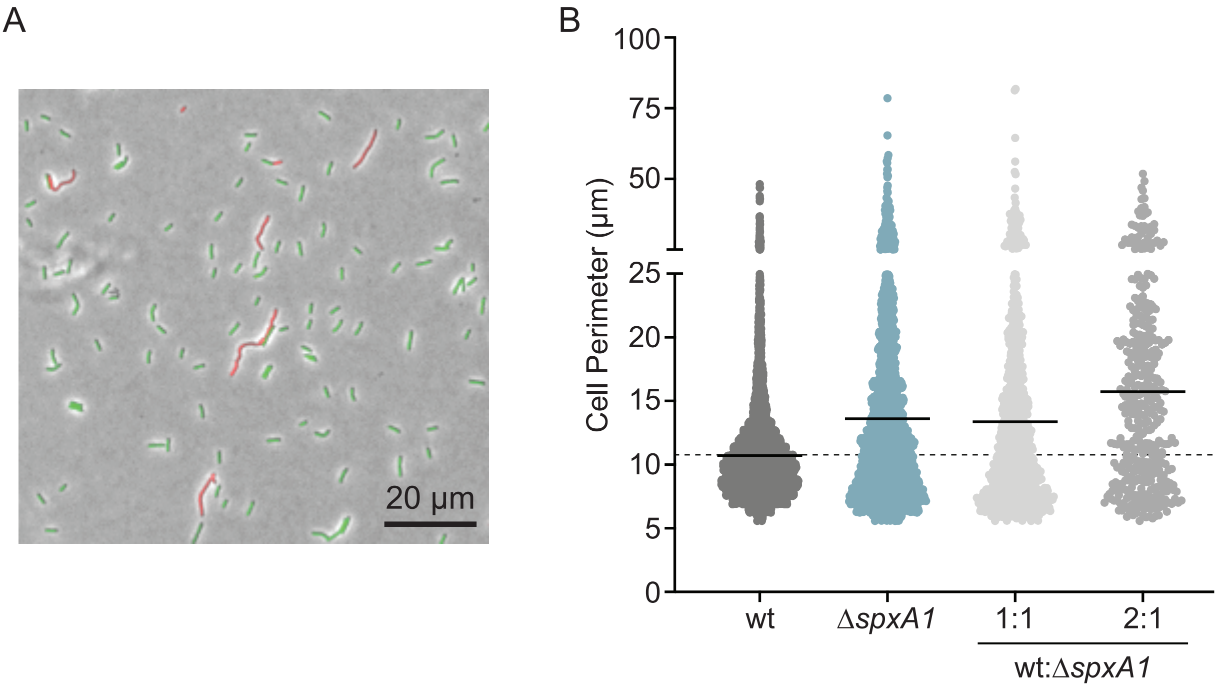
A
20 µm
B
Cell Perimeter (µm)
100
75
50
25
20
15
10
5
0
wt
ΔspxA1
1:1
2:1
wt:ΔspxA1

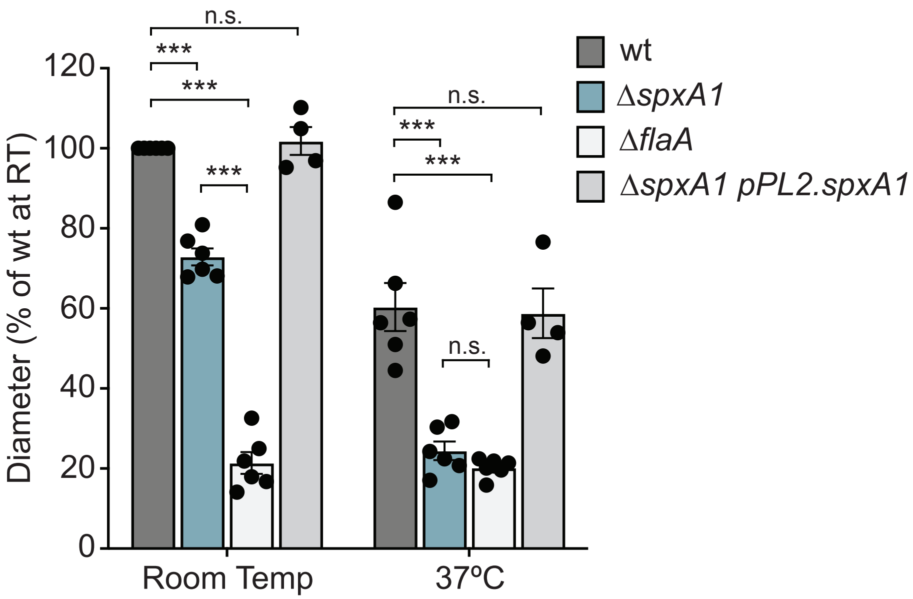
n.s.
***
***
***
***
***
n.s.
n.s.
120
100
80
60
40
20
0
Diameter (% of wt at RT)
Room Temp
37ºC
wt
ΔspxA1
ΔflaA
ΔspxA1 pPL2.spxA1

Table S1. Proteins decreased in abundance in Δ*spxA1* compared to wt

10403S	EGD-e	Protein name	Protein function	Functional Group	wt LFQ average	Δ*spxA1* LFQ average	Fold decrease (wt/Δ*spxA1*)	P value
**Redox & respiration**								
LMRG_01912	lmo2785	Kat	catalase	redox	2.8E+08	5.6E+06	50.1	1.4E-02
LMRG_02735	lmo2390		hypothetical thioredoxin reductase	redox	3.9E+07	3.2E+06	12.3	1.1E-03
LMRG_01267	lmo2113	ChdC	heme peroxidase	redox	1.7E+08	1.9E+07	9.4	4.4E-05
LMRG_02083	lmo0983		glutathione peroxidase	redox	4.2E+07	4.9E+06	8.7	6.0E-03
LMRG_01822	lmo2426	SpxA2	ArsC-family protein	redox	1.2E+08	1.6E+07	7.8	5.7E-04
LMRG_01770	lmo2478	TrxB	thioredoxin reductase	redox	2.2E+08	6.9E+07	3.2	6.6E-05
LMRG_00294	lmo0611	AzoR	FMN-dependent NADH-azoreductase	redox	3.9E+07	1.3E+07	3.0	9.7E-06
LMRG_01585	lmo2247		hypothetical oxidoreductase of aldo/keto reductase family	redox	6.4E+07	2.2E+07	2.8	1.1E-04
LMRG_02734	lmo2389		NADH dehydrogenase	redox	1.5E+08	5.2E+07	2.8	1.3E-03
LMRG_00891	lmo1439	SodA	manganese superoxide dismutase	redox	9.3E+08	3.3E+08	2.8	7.1E-04
LMRG_00679	lmo1233	TrxA	thioredoxin	redox	2.9E+08	1.2E+08	2.4	1.4E-03
LMRG_02241	lmo2694		hypothetical arginine decarboxylase	redox	2.9E+07	1.3E+07	2.1	4.6E-04
LMRG_02352	lmo0103		putative oxidoreductase	redox	1.6E+07	7.3E+06	2.1	2.9E-02
LMRG_01680	lmo2152		putative thioredoxin	redox	5.8E+07	2.8E+07	2.0	3.5E-02
LMRG_01357	lmo1609		thioredoxin	redox	4.5E+07	2.2E+07	2.0	5.2E-03
LMRG_01294	lmo1673	MenB	naphthoate synthase	redox	8.3E+08	4.1E+08	2.0	4.4E-04
LMRG_01223	lmo2072	Rex	redox-sensitive transcriptional repressor	redox	2.8E+08	1.4E+08	2.0	1.2E-03
LMRG_01621	lmo2211	HemH	ferrochelatase	redox	2.9E+08	0	-	1.6E-07
LMRG_01620	lmo2212	HemE	uroporphyrinogen decarboxylase	redox	7.6E+07	0	-	1.2E-04
LMRG_01384	lmo1583	Tpx	thiol peroxidase	redox	7.2E+07	0	-	1.2E-07
LMRG_01641	lmo2191	SpxA1	redox-responsive transcriptional regulator	redox	6.4E+07	0	-	6.7E-06
LMRG_02248	lmo0823		hypothetical oxidoreductase of aldo/keto reductase family	redox	3.6E+07	0	-	2.0E-03
LMRG_01981	lmo2715	CydD	ABC transporter	respiration	5.5E+07	2.0E+06	27.5	1.3E-03
LMRG_01978	lmo2718	CydA	cytochrome d ubiquinol oxidase subunit I	respiration	6.3E+07	4.9E+06	12.9	9.2E-04
LMRG_01980	lmo2716	CydC	transport ATP-binding protein	respiration	5.7E+07	0	-	1.2E-04
**Motility**								
LMRG_00386	lmo0697	FlgE	flagellar hook protein	motility	6.9E+07	3.0E+06	23.3	1.4E-03
LMRG_00378	lmo0690	FlaA	flagellin	motility	2.5E+09	3.6E+08	7.1	4.1E-04
LMRG_00412	lmo0723		methyl-accepting chemotaxis protein	motility	6.1E+08	9.2E+07	6.7	2.9E-03
LMRG_00402	lmo0713	FliF	flagellar M-ring protein	motility	9.0E+07	1.5E+07	6.2	2.3E-05
LMRG_00395	lmo0706	FlgL	flagellar hook-associated protein	motility	4.9E+07	8.1E+06	6.1	2.1E-02
LMRG_02773	lmo1699		methyl-accepting chemotaxis protein	motility	2.3E+08	4.0E+07	5.9	3.3E-03

LMRG_00394	lmo0705	FlgK	flagellar hook-associated protein	motility	1.3E+08	2.2E+07	5.7	2.4E-03
LMRG_00380	lmo0692	CheA	signal transduction histidine kinase	motility	2.4E+08	5.0E+07	4.7	3.5E-04
LMRG_00396	lmo0707	FliD	flagellar hook-associated protein	motility	7.0E+07	1.5E+07	4.6	1.8E-02
LMRG_00379	lmo0691	CheY	chemotaxis regulator	motility	2.5E+08	6.1E+07	4.2	3.9E-05
LMRG_00403	lmo0714	FliG	flagellar motor switch protein	motility	1.2E+08	3.1E+07	3.8	6.5E-04
LMRG_00373	lmo0685	MotA	flagellar motor rotation protein	motility	5.6E+07	1.8E+07	3.2	7.5E-03
LMRG_00377	lmo0689	CheV	chemotaxis protein	motility	4.1E+08	1.3E+08	3.0	1.9E-04
LMRG_00376	lmo0688	GmaR	flagellin glycosyltransferase	motility	2.3E+08	8.4E+07	2.8	2.7E-04
LMRG_00388	lmo0699	FliM	flagellar motor switch protein	motility	2.8E+07	0	-	6.2E-04
LMRG_00389	lmo0700	CheC	chemotaxis protein	motility	2.7E+07	0	-	2.9E-04
**Protein Turnover**								
LMRG_01575	lmo2256		hypothetical peptidase	protein turnover	1.0E+08	2.5E+07	4.1	5.8E-03
LMRG_01644	lmo2188	PepF	oligoendopeptidase F	protein turnover	2.9E+08	9.2E+07	3.1	2.1E-04
LMRG_01389	lmo1578		hypothetical proline dipeptidase	protein turnover	2.5E+08	8.2E+07	3.0	3.5E-05
LMRG_02832	lmo1780	PepT	tripeptide aminopeptidase	protein turnover	2.8E+08	1.0E+08	2.7	2.1E-04
LMRG_02082	lmo0982		hypothetical deblocking aminopeptidase	protein turnover	1.8E+08	6.8E+07	2.6	5.6E-05
LMRG_00844	lmo1392		hypothetical zinc protease	protein turnover	8.6E+07	3.6E+07	2.4	1.5E-03
LMRG_00804	lmo1354		hypothetical aminopeptidase	protein turnover	4.0E+08	1.7E+08	2.3	1.3E-03
LMRG_01382	lmo1585		hypothetical protease IV	protein turnover	3.7E+07	1.6E+07	2.3	4.0E-03
LMRG_01505	lmo2338	PepC	aminopeptidase C	protein turnover	5.6E+07	2.7E+07	2.1	3.8E-04
LMRG_00845	lmo1393		metallopeptidase, M16 family	protein turnover	1.7E+08	8.6E+07	2.0	2.1E-04
**Signal Transduction, Transcription, & Translation**								
LMRG_02239	lmo2692	PstA	c-di-AMP binding signal transduction protein	signal transduction	1.1E+08	2.4E+07	4.7	7.2E-07
LMRG_02064	lmo0965		adenylate cyclase	signal transduction	2.4E+07	7.4E+06	3.3	1.8E-02
LMRG_02218	lmo2673	UspA2	universal stress protein	signal transduction	4.3E+07	1.6E+07	2.8	7.8E-04
LMRG_01165	lmo2016	CspD	cold shock protein	signal transduction	1.5E+09	5.6E+08	2.7	1.2E-04
LMRG_00830	lmo1378	LisK	two-component sensor histidine kinase	signal transduction	4.7E+07	1.9E+07	2.5	4.9E-02
LMRG_01082	lmo1935		protein tyrosine phosphatase	signal transduction	8.7E+07	3.7E+07	2.4	2.6E-04
LMRG_00918	lmo1466	PgpH	cyclic-di-AMP phosphodiesterase	signal transduction	2.7E+07	1.2E+07	2.1	2.1E-02
LMRG_01024	lmo1878	MntR	Mn-dependent transcriptional regulator	transcription	6.4E+07	2.8E+06	23.1	7.3E-05
LMRG_00164	lmo0483		transcriptional regulator, AraC family	transcription	4.4E+07	1.5E+07	3.0	3.7E-05
LMRG_00128	lmo0436		Rrf2 family transcriptional regulator, group III	transcription	2.9E+07	0	-	4.3E-04
LMRG_01690	lmo2557		transcription regulator	transcription	6.8E+06	0	-	2.0E-04

LMRG_02633	lmo0211	RplY	ribosomal protein L25p	translation	5.9E+08	2.0E+08	2.9	2.8E-03
LMRG_01737	lmo2511	Hpf	ribosomal subunit interface protein	translation	9.3E+08	3.2E+08	2.9	1.1E-03
LMRG_01977	lmo2719	TadA	tRNA-specific adenosine deaminase	translation	1.7E+07	0	-	7.4E-06
**Metabolism**								
LMRG_01388	lmo1579	Ald	alanine dehydrogenase	amino acid metabolism	2.4E+08	6.8E+07	3.6	1.8E-04
LMRG_01336	lmo1630	TrpC	indole-3-glycerol phosphate synthase	amino acid metabolism	1.9E+07	9.1E+06	2.1	1.4E-05
LMRG_01873	lmo2825	SerC	phosphoserine aminotransferase	amino acid metabolism	2.8E+07	0	-	2.7E-05
LMRG_02589	lmo0282	AmiE	aliphatic amidase	carbon metabolism	3.2E+07	8.3E+06	3.9	4.7E-02
LMRG_00351	lmo0664		maltose O-acetyltransferase	carbon metabolism	4.5E+07	1.9E+07	2.4	3.2E-03
LMRG_02007	lmo0907		phosphoglycerate mutase family protein	carbon metabolism	1.3E+08	6.0E+07	2.1	1.0E-03
LMRG_01300	lmo1667		L-lactate dehydrogenase	carbon metabolism	2.1E+07	1.0E+07	2.0	5.6E-03
LMRG_01771	lmo2477	GalE	UDP-glucose 4-epimerase	carbon metabolism	4.7E+07	2.0E+07	2.4	4.2E-03
LMRG_01976	lmo2720		acyl-coA synthetase	cofactor metabolism	2.0E+08	5.6E+06	36.1	4.9E-05
LMRG_01823	lmo2425	GcvH	glycine cleavage system H protein	cofactor metabolism	1.8E+08	3.0E+07	5.9	7.0E-05
LMRG_01023	lmo1877	Fhs	formate-tetrahydrofolate ligase	cofactor metabolism	2.7E+08	5.0E+07	5.4	5.0E-06
LMRG_01080	lmo1933	FolE	GTP cyclohydrolase I	cofactor metabolism	7.4E+07	2.9E+07	2.5	1.4E-03
LMRG_02030	lmo0931	LplA1	lipoate-protein ligase A	cofactor metabolism	1.4E+08	6.2E+07	2.2	5.2E-03
LMRG_02359	lmo0110		esterase/lipase	lipid metabolism	3.3E+07	2.6E+06	12.5	7.8E-04
LMRG_01240	lmo2089		esterase/lipase	lipid metabolism	4.3E+08	8.8E+07	4.9	2.6E-05
LMRG_02762	lmo1688	FabL	enoyl-acyl carrier protein reductase I	lipid metabolism	2.6E+07	5.6E+06	4.7	2.7E-02
LMRG_00462	lmo0774		diacylglycerol kinase-related protein	lipid metabolism	1.5E+08	5.2E+07	2.9	2.8E-05
LMRG_00820	lmo1370	Buk	butyrate kinase	lipid metabolism	3.4E+07	0	-	3.9E-05
**Transport**								
LMRG_00672	lmo1226		MMPL family transporter	transport	6.0E+07	2.0E+06	30.4	7.6E-03
LMRG_02264	lmo0841	LmcA1	calcium-transporting ATPase	transport	5.8E+07	9.0E+06	6.5	3.5E-03
LMRG_02717	lmo2372		ABC transporter, ATP-binding protein	transport	1.5E+08	2.8E+07	5.3	5.5E-04
LMRG_02716	lmo2371		ABC transporter, permease protein	transport	1.2E+08	3.2E+07	3.8	7.2E-07
LMRG_02117	lmo1017		PTS system, glucose-specific IIA component	transport	1.5E+08	4.4E+07	3.3	3.4E-04
LMRG_01617	lmo2215	EcsA	ABC transporter, ATP-binding protein	transport	2.1E+07	6.8E+06	3.1	1.5E-02
LMRG_02114	lmo1014	GbuA	glycine betaine ABC transport system, ATP-binding protein	transport	2.7E+08	1.0E+08	2.7	7.3E-03
LMRG_00354	lmo0667		ABC transporter, ATP-binding protein	transport	4.4E+07	1.7E+07	2.6	4.0E-02
LMRG_02801	lmo2139		ABC transporter, ATP-binding protein	transport	1.2E+07	0	-	1.1E-04

**DNA repair & metabolism**								
LMRG_01403	lmo1564	MutM	formamidopyrimidine-DNA glycosylase	DNA repair	3.1E+07	9.5E+06	3.3	2.3E-03
LMRG_01200	lmo2050		excinuclease ABC subunit A	DNA repair	2.0E+07	0	-	2.9E-05
LMRG_00915	lmo1463		cytidine deaminase	nucleotide metabolism	1.1E+08	5.2E+07	2.2	4.4E-04
**Unknown & Other**								
LMRG_00146	lmo0454		MoxR family ATPase	unknown	3.0E+07	1.1E+06	26.8	1.3E-03
LMRG_00274	lmo0592		hypothetical protein	unknown	3.7E+08	3.9E+07	9.3	4.5E-04
LMRG_00372	lmo0684		hypothetical protein	unknown	3.5E+07	4.9E+06	7.3	8.6E-03
LMRG_00143	lmo0451		hypothetical protein	unknown	2.2E+07	3.4E+06	6.4	1.2E-02
LMRG_00407	lmo0718		hypothetical protein	unknown	1.3E+08	3.3E+07	3.8	3.7E-04
LMRG_00479	lmo0791		hypothetical protein	unknown	1.4E+08	3.9E+07	3.7	4.5E-05
LMRG_00393	lmo0704		hypothetical protein	unknown	6.0E+07	1.8E+07	3.3	2.7E-03
LMRG_01234	lmo2083		hypothetical protein	unknown	6.8E+07	2.0E+07	3.3	8.1E-05
LMRG_00527	lmo1065		hypothetical protein	unknown	7.2E+07	2.2E+07	3.3	4.7E-04
LMRG_01358	lmo1608		hypothetical protein	unknown	2.4E+08	8.6E+07	2.8	1.3E-02
LMRG_02630	lmo0208		hypothetical protein	unknown	1.1E+08	4.7E+07	2.4	4.4E-03
LMRG_02609	lmo0267		glyoxalase family protein	unknown	2.1E+07	8.9E+06	2.4	3.0E-02
LMRG_01230	lmo2079		hypothetical protein	unknown	1.3E+08	5.5E+07	2.3	4.0E-04
LMRG_01937	lmo2759		putative ADP-ribose-binding protein	unknown	7.4E+07	3.2E+07	2.3	4.4E-03
LMRG_01665	lmo2167		hypothetical protein	unknown	1.5E+08	6.8E+07	2.2	1.8E-02
LMRG_01366	lmo1601		general stress protein	unknown	8.5E+08	4.0E+08	2.1	1.0E-03
LMRG_01616	lmo2216		HIT domain-containing protein	unknown	5.9E+07	2.8E+07	2.1	4.6E-02
LMRG_01666	lmo2166		cyclic nucleotide-binding protein	unknown	7.3E+07	3.4E+07	2.1	1.9E-04
LMRG_01905	lmo2792		non-specific DNA-binding protein	unknown	5.3E+08	2.5E+08	2.1	2.3E-03
LMRG_01365	lmo1602		general stress protein	unknown	5.1E+08	2.5E+08	2.1	3.5E-04
LMRG_02403	lmo0158		Cof-like hydrolase	unknown	2.8E+07	1.4E+07	2.1	1.0E-02
LMRG_00521	lmo1059		hypothetical protein	unknown	3.6E+08	1.8E+08	2.0	2.3E-03
LMRG_00833	lmo1381		putative acylphosphatase	unknown	5.2E+07	0	-	9.0E-06
LMRG_02786	lmo0811		carbonic anhydrase	unknown	4.8E+07	0	-	5.6E-06
LMRG_01842	lmo2406		uncharacterized conserved protein	unknown	2.9E+07	0	-	1.3E-06
LMRG_02267	lmo0844		hypothetical protein	unknown	1.1E+07	0	-	3.9E-06
LMRG_00563	lmo1121		hypothetical protein	unknown	1.1E+07	0	-	2.1E-06

Table S2. Proteins increased in abundance in Δ*spxA1* compared to wt

10403S	EGD-e	Protein name	Protein function	Functional Group	wt LFQ average	Δ*spxA1* LFQ average	Fold increase (Δ*spxA1*/wt)	P value
**Protein synthesis & turnover**								
LMRG_01642	lmo2190	MecA	putative proteolysis adaptor protein	protein turnover	3.8E+07	7.4E+08	19.3	1.8E-04
LMRG_02097	lmo0997	ClpE	ATP-dependent Clp protease, ATP-binding subunit	protein turnover	2.3E+08	2.3E+09	9.9	1.1E-03
LMRG_02579	lmo0292	HtrA	serine protease	protein turnover	2.3E+08	6.5E+08	2.8	2.2E-02
LMRG_00922	lmo1469	RpsU	SSU ribosomal protein S21p	translation	2.7E+08	7.9E+08	2.9	2.9E-03
LMRG_02201	lmo2656	RpsL	SSU ribosomal protein S12p	translation	1.4E+09	3.9E+09	2.7	3.8E-03
LMRG_02652	lmo0257	RtcB	tRNA splicing RNA ligase	translation	1.6E+07	4.1E+07	2.6	2.3E-02
LMRG_02163	lmo2619	RpsZ	SSU ribosomal protein S14p	translation	2.4E+08	4.9E+08	2.1	3.3E-02
**Cell envelope biogenesis**								
LMRG_01722	lmo2526	MurA	UDP-N-acetylglucosamine 1-carboxyvinyltransferase	cell envelope biogenesis	6.3E+08	1.6E+09	2.5	4.7E-05
LMRG_00264	lmo0582	lap/p60	P60 autolysin	cell envelope biogenesis	2.8E+08	6.1E+08	2.1	1.0E-03
**Metabolism**								
LMRG_01146	lmo1998		putative glucosamine-fructose-6-phosphate aminotransferase	amino acid metabolism	2.0E+07	5.2E+07	2.6	8.4E-04
LMRG_02456	lmo0027		PTS system component EIIB	carbon metabolism	1.2E+07	3.3E+07	2.8	1.7E-03
LMRG_02747	lmo0182		glycosyl hydrolase	carbon metabolism	4.1E+07	1.1E+08	2.6	1.9E-02
LMRG_02748	lmo0183		alpha-glucosidase	carbon metabolism	4.0E+07	9.2E+07	2.3	3.2E-02
LMRG_00094	lmo0401		alpha-mannosidase	carbon metabolism	0.0E+00	3.3E+07	-	3.3E-07
LMRG_00492	lmo1031		unknown pentose isomerase	carbon metabolism	0.0E+00	3.0E+07	-	1.1E-03
LMRG_02205	lmo2660	Tkt	transketolase, pentose phosphate pathway	carbon metabolism	0.0E+00	1.5E+07	-	5.1E-05
LMRG_01049	lmo1902	PanB	phosphopantothenate biosynthesis	cofactor metabolism	2.8E+08	1.6E+09	5.6	4.1E-06
LMRG_01048	lmo1901	PanC	phosphopantothenate biosynthesis	cofactor metabolism	8.7E+07	3.2E+08	3.6	6.9E-05
**Transport**								
LMRG_02532	lmo1739		amino acid ABC transporter, ATP-binding protein	transport	2.5E+06	1.9E+07	7.6	7.7E-03
LMRG_01944	lmo2752		ABC transporter, ATP-binding/permease protein	transport	4.8E+07	1.7E+08	3.5	2.8E-03
LMRG_00994	lmo1847	MntA	manganese ABC transporter, periplasmic-binding protein	transport	6.8E+07	2.2E+08	3.3	4.8E-06
LMRG_01945	lmo2751		ABC transporter, ATP-binding protein	transport	8.0E+07	2.6E+08	3.2	1.6E-03
LMRG_02533	lmo1738		amino acid ABC transporter, amino acid-binding protein	transport	9.1E+06	2.4E+07	2.6	2.8E-03
LMRG_00996	lmo1849	MntB	manganese ABC transporter, ATP-binding protein	transport	6.6E+07	1.7E+08	2.6	1.3E-04
LMRG_00327	lmo0641	FrvA	heme-transporting ATPase	transport	8.3E+07	2.1E+08	2.6	1.0E-04
LMRG_00874	lmo1422		glycine betaine ABC transport system	transport	1.3E+07	2.8E+07	2.1	5.3E-03

**DNA repair & metabolism**								
LMRG_01611	lmo2221		DNA double-strand break repair rad50 ATPase	DNA repair	3.1E+06	1.5E+07	4.9	2.0E-02
LMRG_02496	lmo1775	PurE	inosine-5'-phosphate biosynthesis II	nucleotide metabolism	4.1E+07	1.1E+08	2.7	1.0E-02
LMRG_02499	lmo1772	PurC	inosine-5'-phosphate biosynthesis II	nucleotide metabolism	5.8E+07	1.4E+08	2.4	1.9E-02
LMRG_02502	lmo1769	PurL	phosphoribosylformyl-glycinamidine synthase	nucleotide metabolism	7.7E+07	1.7E+08	2.2	4.9E-03
LMRG_02504	lmo1767	PurM	phosphoribosylformyl-glycinamidine cycloligase	nucleotide metabolism	2.9E+07	6.4E+07	2.2	2.2E-02
LMRG_02498	lmo1773	PurB	adenylosuccinate lyase	nucleotide metabolism	2.8E+08	6.2E+08	2.2	2.9E-03
LMRG_02503	lmo1768	PurF	amidophosphoribosyl-transferase	nucleotide metabolism	1.5E+07	3.0E+07	2.0	2.9E-03
LMRG_02507	lmo1764	PurD	phosphoribosylamine-glycine ligase	nucleotide metabolism	4.8E+07	9.8E+07	2.0	9.6E-03
LMRG_02485	lmo0055	PurA	adenylosuccinate synthetase	nucleotide metabolism	9.6E+08	1.9E+09	2.0	1.1E-03
**Unknown & Other**								
LMRG_01715	lmo2533	AtpF	ATP synthase B chain	energy production	5.4E+08	1.1E+09	2.1	1.3E-03
LMRG_00129	lmo0437		oxidoreductase	redox	6.9E+07	6.8E+08	9.8	1.2E-05
LMRG_00928	lmo1475	HrcA	heat-inducible transcription repressor	transcription	2.7E+07	8.9E+07	3.3	3.4E-03
LMRG_02677	lmo0229	CtsR	transcriptional regulator	transcription	3.8E+07	8.9E+07	2.4	2.0E-04
LMRG_01608	lmo2224		hypothetical protein	unknown	4.2E+06	1.7E+07	4.2	4.2E-02
LMRG_00177	lmo0496		hypothetical protein	unknown	2.8E+07	8.2E+07	2.9	4.2E-03
LMRG_01577	lmo2254		permease	unknown	8.9E+06	2.3E+07	2.6	3.5E-02
LMRG_02795	lmo0802		hypothetical protein	unknown	1.1E+07	2.3E+07	2.2	2.1E-02
LMRG_02486	lmo0056		secreted protein	unknown	1.8E+07	3.8E+07	2.2	6.2E-03
LMRG_01841	lmo2407		hypothetical protein	unknown	3.6E+07	7.4E+07	2.1	1.0E-02

Table S3. Complete list of proteins detected by whole cell proteomics

10403S	EGD-e	Protein Name	avg wt LFQ	avg Δ*spxA1* LFQ	Δ*spxA1*/wt LFQ	wt/Δ*spxA1* LFQ	Unpaired t-test
LMRG_00001	lmo0304		1.4E+06	0.0E+00	0.0E+00	#DIV/0!	3.7E-01
LMRG_00002	lmo0305		7.5E+07	4.1E+07	5.5E-01	1.8E+00	5.6E-03
LMRG_00003	lmo0306		1.3E+06	0.0E+00	0.0E+00	#DIV/0!	3.7E-01
LMRG_00007	lmo0314		0.0E+00	4.3E+05	#DIV/0!	0.0E+00	3.7E-01
LMRG_00012	lmo0319		1.1E+08	1.5E+08	1.4E+00	7.3E-01	1.7E-02
LMRG_00015	lmo0323		8.3E+06	1.1E+07	1.4E+00	7.3E-01	5.7E-01
LMRG_00026	lmo0334		0.0E+00	4.4E+05	#DIV/0!	0.0E+00	3.7E-01
LMRG_00043	lmo0352		1.1E+07	1.1E+07	9.3E-01	1.1E+00	4.0E-01
LMRG_00045	lmo0354		1.2E+09	1.7E+09	1.5E+00	6.8E-01	9.0E-03
LMRG_00046	lmo0355		2.2E+10	1.6E+10	7.1E-01	1.4E+00	1.7E-03
LMRG_00047	lmo0356		2.7E+08	1.6E+08	5.8E-01	1.7E+00	8.4E-07
LMRG_00055	lmo0363		5.1E+06	6.1E+06	1.2E+00	8.3E-01	2.7E-01
LMRG_00058	lmo0366		6.9E+05	0.0E+00	0.0E+00	#DIV/0!	3.7E-01
LMRG_00061	lmo0369		4.7E+08	7.6E+08	1.6E+00	6.2E-01	1.0E-03
LMRG_00062	lmo0370	PhnA	3.6E+08	4.7E+08	1.3E+00	7.6E-01	7.5E-03
LMRG_00063	lmo0371		0.0E+00	3.8E+06	#DIV/0!	0.0E+00	1.2E-01
LMRG_00067	lmo0375		6.7E+07	6.9E+07	1.0E+00	9.7E-01	8.4E-01
LMRG_00069	lmo0377		1.5E+06	0.0E+00	0.0E+00	#DIV/0!	3.7E-01
LMRG_00075	lmo0382		9.1E+06	1.0E+07	1.1E+00	8.9E-01	4.3E-01
LMRG_00080	lmo0387		8.6E+07	6.9E+07	8.1E-01	1.2E+00	6.5E-02
LMRG_00083	lmo0390	Ung	2.3E+07	1.1E+07	4.9E-01	2.0E+00	1.9E-01
LMRG_00084	lmo0391		2.8E+06	0.0E+00	0.0E+00	#DIV/0!	3.7E-01
LMRG_00085	lmo0392	FloA	6.8E+08	4.3E+08	6.4E-01	1.6E+00	6.1E-04
LMRG_00086	lmo0393		6.0E+06	1.7E+06	2.9E-01	3.5E+00	1.2E-01
LMRG_00088	lmo0395		0.0E+00	4.1E+07	#DIV/0!	0.0E+00	3.7E-01
LMRG_00089	lmo0396	ProC	5.3E+07	4.1E+07	7.7E-01	1.3E+00	1.9E-02
LMRG_00091	lmo0398		0.0E+00	1.4E+07	#DIV/0!	0.0E+00	1.1E-03
LMRG_00092	lmo0399		3.7E+06	1.6E+07	4.4E+00	2.3E-01	4.0E-03
LMRG_00094	lmo0401		0.0E+00	3.3E+07	#DIV/0!	0.0E+00	3.3E-07
LMRG_00096	lmo0403		4.5E+06	1.9E+06	4.3E-01	2.3E+00	4.4E-01
LMRG_00099	lmo0406		4.3E+07	3.8E+07	8.9E-01	1.1E+00	1.3E-01
LMRG_00100	lmo0407		8.7E+06	7.5E+06	8.6E-01	1.2E+00	8.5E-01
LMRG_00103	lmo0410		4.7E+06	0.0E+00	0.0E+00	#DIV/0!	9.1E-05
LMRG_00104	lmo0412		0.0E+00	1.1E+06	#DIV/0!	0.0E+00	3.7E-01
LMRG_00105	lmo0413		0.0E+00	2.4E+06	#DIV/0!	0.0E+00	3.7E-01
LMRG_00107	lmo0415	PgdA	1.2E+09	1.9E+09	1.5E+00	6.6E-01	4.3E-02
LMRG_00118	lmo0426		5.3E+06	1.2E+07	2.2E+00	4.5E-01	9.3E-02
LMRG_00119	lmo0427		0.0E+00	2.2E+07	#DIV/0!	0.0E+00	1.3E-01
LMRG_00120	lmo0428		0.0E+00	1.8E+06	#DIV/0!	0.0E+00	3.7E-01
LMRG_00128	lmo0436		2.9E+07	0.0E+00	0.0E+00	#DIV/0!	4.3E-04
LMRG_00129	lmo0437		6.9E+07	6.8E+08	9.8E+00	1.0E-01	1.2E-05
LMRG_00132	lmo0440		4.6E+06	0.0E+00	0.0E+00	#DIV/0!	3.7E-01
LMRG_00133	lmo0441	PbpB3	6.0E+07	5.9E+07	9.9E-01	1.0E+00	9.3E-01
LMRG_00135	lmo0443		5.6E+08	7.7E+08	1.4E+00	7.2E-01	7.8E-03
LMRG_00143	lmo0451		2.2E+07	3.4E+06	1.6E-01	6.4E+00	1.2E-02
LMRG_00145	lmo0453		8.5E+05	0.0E+00	0.0E+00	#DIV/0!	3.7E-01
LMRG_00146	lmo0454		3.0E+07	1.1E+06	3.7E-02	2.7E+01	1.3E-03
LMRG_00150	lmo0458		0.0E+00	2.9E+06	#DIV/0!	0.0E+00	3.7E-01
LMRG_00163	lmo0482	RlmN	3.0E+08	4.4E+08	1.4E+00	7.0E-01	1.4E-03
LMRG_00164	lmo0483		4.4E+07	1.5E+07	3.3E-01	3.0E+00	3.7E-05
LMRG_00166	lmo0485		8.1E+05	0.0E+00	0.0E+00	#DIV/0!	3.7E-01
LMRG_00167	lmo0486	RpmF	1.3E+08	1.1E+08	8.4E-01	1.2E+00	1.2E-01
LMRG_00168	lmo0487		1.6E+08	1.3E+08	8.1E-01	1.2E+00	1.1E-01
LMRG_00169	lmo0488	QuiR	0.0E+00	2.2E+06	#DIV/0!	0.0E+00	3.7E-01
LMRG_00170	lmo0489		0.0E+00	4.2E+06	#DIV/0!	0.0E+00	1.2E-01
LMRG_00171	lmo0490	AroE	1.2E+08	1.2E+08	9.9E-01	1.0E+00	7.3E-01
LMRG_00172	lmo0491	AroD	1.5E+08	1.6E+08	1.1E+00	9.2E-01	8.2E-05
LMRG_00173	lmo0492		0.0E+00	6.6E+05	#DIV/0!	0.0E+00	3.7E-01
LMRG_00174	lmo0493		7.7E+06	1.5E+07	1.9E+00	5.2E-01	5.8E-02
LMRG_00175	lmo0494		5.2E+07	7.3E+07	1.4E+00	7.2E-01	1.3E-03
LMRG_00177	lmo0496		2.8E+07	8.2E+07	2.9E+00	3.4E-01	4.2E-03
LMRG_00178	lmo0497		0.0E+00	1.3E+06	#DIV/0!	0.0E+00	3.7E-01
LMRG_00190	lmo0509	Prs	4.3E+08	4.4E+08	1.0E+00	9.8E-01	7.7E-01
LMRG_00192	lmo0511		0.0E+00	4.9E+05	#DIV/0!	0.0E+00	3.7E-01
LMRG_00193	lmo0512		6.1E+07	4.4E+07	7.2E-01	1.4E+00	1.1E-03
LMRG_00194	lmo0513		1.0E+07	4.7E+06	4.5E-01	2.2E+00	8.0E-02
LMRG_00196	lmo0515		4.3E+07	2.5E+07	5.7E-01	1.8E+00	2.2E-01
LMRG_00197	lmo0516		2.3E+07	2.4E+07	1.0E+00	9.8E-01	8.1E-01
LMRG_00200	lmo0519	MdrA	0.0E+00	1.0E+06	#DIV/0!	0.0E+00	3.7E-01
LMRG_00201	lmo0520		6.5E+06	6.5E+06	1.0E+00	1.0E+00	1.0E+00
LMRG_00202	lmo0521		2.3E+08	1.7E+08	7.4E-01	1.4E+00	1.7E-02
LMRG_00203	lmo0522		1.6E+06	0.0E+00	0.0E+00	#DIV/0!	3.7E-01
LMRG_00205	lmo0524		8.6E+06	9.7E+05	1.1E-01	8.9E+00	2.4E-02
LMRG_00211	lmo0529	PssC	4.6E+07	5.5E+07	1.2E+00	8.3E-01	3.0E-01
LMRG_00212	lmo0530	PssD	6.4E+07	5.9E+07	9.1E-01	1.1E+00	1.8E-01
LMRG_00213	lmo0531	PssE	3.5E+07	3.3E+07	9.3E-01	1.1E+00	2.9E-01
LMRG_00214	lmo0532		2.5E+07	1.7E+07	7.1E-01	1.4E+00	2.0E-02
LMRG_00215	lmo0533		1.5E+07	1.2E+07	7.8E-01	1.3E+00	1.6E-01
LMRG_00216	lmo0534		1.1E+08	1.0E+08	9.3E-01	1.1E+00	3.5E-01
LMRG_00217	lmo0535	LacI	1.1E+07	0.0E+00	0.0E+00	#DIV/0!	1.6E-04
LMRG_00218	lmo0536		2.5E+07	2.9E+07	1.1E+00	8.7E-01	4.0E-01
LMRG_00221	lmo0539	LacD	1.0E+09	1.4E+09	1.4E+00	7.0E-01	1.3E-02

LMRG_00222	lmo0540	PbpX	1.8E+07	2.4E+07	1.3E+00	7.5E-01	5.5E-01
LMRG_00223	lmo0541		9.0E+06	1.4E+07	1.5E+00	6.5E-01	9.6E-03
LMRG_00229	lmo0547		7.1E+06	2.0E+07	2.8E+00	3.6E-01	3.8E-01
LMRG_00235	lmo0553	CbpA	6.5E+08	5.1E+08	7.9E-01	1.3E+00	3.2E-02
LMRG_00236	lmo0554		1.9E+08	2.3E+08	1.3E+00	8.0E-01	3.6E-02
LMRG_00238	lmo0556		3.3E+07	2.3E+07	7.0E-01	1.4E+00	4.5E-02
LMRG_00239	lmo0557		2.1E+07	1.6E+07	7.5E-01	1.3E+00	1.5E-01
LMRG_00240	lmo0558	Pgl	9.6E+08	5.5E+08	5.7E-01	1.8E+00	1.1E-04
LMRG_00241	lmo0559		7.3E+06	5.7E+06	7.8E-01	1.3E+00	7.5E-01
LMRG_00242	lmo0560	GdhA	4.3E+08	3.8E+08	8.9E-01	1.1E+00	5.8E-03
LMRG_00252	lmo0570	HisJ	0.0E+00	9.7E+05	#DIV/0!	0.0E+00	3.7E-01
LMRG_00254	lmo0572		7.8E+06	5.3E+06	6.8E-01	1.5E+00	4.2E-01
LMRG_00256	lmo0574	GmuD	4.2E+07	2.9E+07	6.7E-01	1.5E+00	1.1E-02
LMRG_00257	lmo0575		1.8E+07	1.5E+07	8.6E-01	1.2E+00	2.0E-01
LMRG_00260	lmo0578		2.8E+06	0.0E+00	0.0E+00	#DIV/0!	1.2E-01
LMRG_00261	lmo0579		6.0E+06	0.0E+00	0.0E+00	#DIV/0!	3.7E-01
LMRG_00262	lmo0580		1.2E+07	0.0E+00	0.0E+00	#DIV/0!	5.5E-07
LMRG_00263	lmo0581		5.3E+07	3.8E+07	7.2E-01	1.4E+00	4.9E-02
LMRG_00264	lmo0582	CwhA	2.8E+08	6.1E+08	2.1E+00	4.7E-01	1.0E-03
LMRG_00265	lmo0583	SecA2	6.9E+07	5.9E+07	8.6E-01	1.2E+00	8.8E-02
LMRG_00266	lmo0584		7.6E+06	2.8E+06	3.6E-01	2.8E+00	3.6E-01
LMRG_00274	lmo0592		3.7E+08	3.9E+07	1.1E-01	9.3E+00	4.5E-04
LMRG_00275	lmo0593		0.0E+00	3.2E+05	#DIV/0!	0.0E+00	3.7E-01
LMRG_00277	lmo0595		1.0E+06	0.0E+00	0.0E+00	#DIV/0!	3.7E-01
LMRG_00282	lmo0599	LltR	2.5E+06	4.0E+06	1.6E+00	6.3E-01	6.7E-01
LMRG_00283	lmo0600		2.7E+07	2.7E+07	1.0E+00	9.8E-01	8.9E-01
LMRG_00284	lmo0601		3.9E+07	3.4E+07	8.6E-01	1.2E+00	3.4E-02
LMRG_00289	lmo0606		0.0E+00	5.8E+06	#DIV/0!	0.0E+00	4.0E-04
LMRG_00290	lmo0607		6.7E+07	1.1E+08	1.6E+00	6.4E-01	6.9E-03
LMRG_00291	lmo0608		7.2E+07	1.2E+08	1.6E+00	6.3E-01	7.0E-03
LMRG_00292	lmo0609		2.2E+07	0.0E+00	0.0E+00	#DIV/0!	4.8E-04
LMRG_00294	lmo0611	AzoR	3.9E+07	1.3E+07	3.3E-01	3.0E+00	9.7E-06
LMRG_00296	lmo0613		8.3E+07	4.9E+07	5.9E-01	1.7E+00	1.3E-03
LMRG_00303	lmo0620		4.0E+07	5.0E+07	1.3E+00	8.0E-01	9.1E-02
LMRG_00309	lmo0626	GtlB	1.6E+07	9.1E+06	5.7E-01	1.8E+00	2.1E-01
LMRG_00318	lmo0635		1.2E+07	1.7E+07	1.4E+00	7.1E-01	1.3E-01
LMRG_00319	lmo0636		1.2E+06	0.0E+00	0.0E+00	#DIV/0!	3.7E-01
LMRG_00320	lmo0637		2.8E+07	2.8E+07	9.9E-01	1.0E+00	8.0E-01
LMRG_00326	lmo0640		1.3E+08	7.5E+07	5.7E-01	1.8E+00	1.1E-02
LMRG_00327	lmo0641	FrvA	8.3E+07	2.1E+08	2.6E+00	3.9E-01	1.0E-04
LMRG_00331	lmo0644	LtaP	8.9E+06	0.0E+00	0.0E+00	#DIV/0!	1.2E-01
LMRG_00332	lmo0645		3.4E+06	0.0E+00	0.0E+00	#DIV/0!	3.7E-01
LMRG_00333	lmo0646		9.3E+05	0.0E+00	0.0E+00	#DIV/0!	3.7E-01
LMRG_00337	lmo0650		0.0E+00	5.7E+06	#DIV/0!	0.0E+00	3.7E-01
LMRG_00338	lmo0651	MouR	0.0E+00	0.0E+00	#DIV/0!	#DIV/0!	#DIV/0!
LMRG_00339	lmo0652		8.7E+06	0.0E+00	0.0E+00	#DIV/0!	1.2E-03
LMRG_00340	lmo0653		2.9E+07	0.0E+00	0.0E+00	#DIV/0!	3.5E-05
LMRG_00341	lmo0654		6.4E+07	5.4E+07	8.4E-01	1.2E+00	1.1E-01
LMRG_00342	lmo0655		0.0E+00	1.0E+06	#DIV/0!	0.0E+00	3.7E-01
LMRG_00345	lmo0658		6.9E+05	0.0E+00	0.0E+00	#DIV/0!	3.7E-01
LMRG_00349	lmo0662	ThiD	4.1E+08	2.4E+08	5.8E-01	1.7E+00	1.9E-04
LMRG_00350	lmo0663		1.0E+08	5.5E+07	5.2E-01	1.9E+00	3.9E-04
LMRG_00351	lmo0664		4.5E+07	1.9E+07	4.1E-01	2.4E+00	3.2E-03
LMRG_00352	lmo0665		8.7E+05	0.0E+00	0.0E+00	#DIV/0!	3.7E-01
LMRG_00354	lmo0667		4.4E+07	1.7E+07	3.9E-01	2.6E+00	4.0E-02
LMRG_00357	lmo0669		0.0E+00	2.2E+06	#DIV/0!	0.0E+00	1.2E-01
LMRG_00361	lmo0673		1.1E+07	1.8E+07	1.6E+00	6.2E-01	3.0E-01
LMRG_00362	lmo0674	MogR	2.3E+07	3.7E+07	1.6E+00	6.3E-01	1.2E-02
LMRG_00364	lmo0676	FliP	0.0E+00	9.7E+05	#DIV/0!	0.0E+00	3.7E-01
LMRG_00368	lmo0680	FlhA	2.4E+06	0.0E+00	0.0E+00	#DIV/0!	1.2E-01
LMRG_00369	lmo0681	FlhF	1.3E+07	0.0E+00	0.0E+00	#DIV/0!	7.4E-04
LMRG_00371	lmo0683	CheR	2.5E+05	0.0E+00	0.0E+00	#DIV/0!	3.7E-01
LMRG_00372	lmo0684		3.5E+07	4.9E+06	1.4E-01	7.3E+00	8.6E-03
LMRG_00373	lmo0685	MotA	5.6E+07	1.8E+07	3.1E-01	3.2E+00	7.5E-03
LMRG_00374	lmo0686	MotB	1.4E+07	3.3E+06	2.4E-01	4.3E+00	1.2E-02
LMRG_00375	lmo0687		3.7E+06	0.0E+00	0.0E+00	#DIV/0!	4.0E-03
LMRG_00376	lmo0688	GmaR	2.3E+08	8.4E+07	3.6E-01	2.8E+00	2.7E-04
LMRG_00377	lmo0689	CheV	4.1E+08	1.3E+08	3.3E-01	3.0E+00	1.9E-04
LMRG_00378	lmo0690	FlaA	2.5E+09	3.6E+08	1.4E-01	7.1E+00	4.1E-04
LMRG_00379	lmo0691	CheY	2.5E+08	6.1E+07	2.4E-01	4.2E+00	3.9E-05
LMRG_00380	lmo0692	CheA	2.4E+08	5.0E+07	2.1E-01	4.7E+00	3.5E-04
LMRG_00381	lmo0693	FliY	8.2E+05	0.0E+00	0.0E+00	#DIV/0!	3.7E-01
LMRG_00384	lmo0695	FliK	2.0E+06	0.0E+00	0.0E+00	#DIV/0!	3.7E-01
LMRG_00385	lmo0696	FlgD	2.0E+07	0.0E+00	0.0E+00	#DIV/0!	5.7E-04
LMRG_00386	lmo0697	FlgE	6.9E+07	3.0E+06	4.3E-02	2.3E+01	1.4E-03
LMRG_00387	lmo0698		1.6E+07	0.0E+00	0.0E+00	#DIV/0!	8.4E-05
LMRG_00388	lmo0699	FliM	2.8E+07	0.0E+00	0.0E+00	#DIV/0!	6.2E-04
LMRG_00389	lmo0700	FliY	2.7E+07	0.0E+00	0.0E+00	#DIV/0!	2.9E-04
LMRG_00390	lmo0701		1.5E+06	0.0E+00	0.0E+00	#DIV/0!	3.7E-01
LMRG_00391	lmo0702		3.8E+06	0.0E+00	0.0E+00	#DIV/0!	3.7E-01
LMRG_00392	lmo0703		1.8E+06	0.0E+00	0.0E+00	#DIV/0!	3.7E-01
LMRG_00393	lmo0704		6.0E+07	1.8E+07	3.0E-01	3.3E+00	2.7E-03
LMRG_00394	lmo0705	FlgK	1.3E+08	2.2E+07	1.8E-01	5.7E+00	2.4E-03
LMRG_00395	lmo0706	FlgL	4.9E+07	8.1E+06	1.6E-01	6.1E+00	2.1E-02
LMRG_00396	lmo0707	FliD	7.0E+07	1.5E+07	2.2E-01	4.6E+00	1.8E-02
LMRG_00397	lmo0708	FliS	1.8E+07	0.0E+00	0.0E+00	#DIV/0!	6.6E-03

LMRG_00398	lmo0709		1.1E+07	0.0E+00	0.0E+00	#DIV/0!	1.5E-03
LMRG_00399	lmo0710	FlgB	4.5E+06	0.0E+00	0.0E+00	#DIV/0!	1.2E-01
LMRG_00400	lmo0711	FlgC	1.3E+06	0.0E+00	0.0E+00	#DIV/0!	3.7E-01
LMRG_00402	lmo0713	FliF	9.0E+07	1.5E+07	1.6E-01	6.2E+00	2.3E-05
LMRG_00403	lmo0714	FliG	1.2E+08	3.1E+07	2.7E-01	3.8E+00	6.5E-04
LMRG_00404	lmo0715	FliH	6.1E+06	0.0E+00	0.0E+00	#DIV/0!	2.4E-05
LMRG_00405	lmo0716	FliI	3.0E+05	0.0E+00	0.0E+00	#DIV/0!	3.7E-01
LMRG_00406	lmo0717		4.5E+06	0.0E+00	0.0E+00	#DIV/0!	1.2E-01
LMRG_00407	lmo0718		1.3E+08	3.3E+07	2.6E-01	3.8E+00	3.7E-04
LMRG_00408	lmo0719	LftR	8.6E+06	7.5E+06	8.7E-01	1.2E+00	1.8E-01
LMRG_00409	lmo0720	LftS	1.2E+07	0.0E+00	0.0E+00	#DIV/0!	1.4E-04
LMRG_00410	lmo0721		0.0E+00	1.2E+07	#DIV/0!	0.0E+00	1.7E-03
LMRG_00411	lmo0722		1.1E+08	1.8E+08	1.6E+00	6.2E-01	1.9E-02
LMRG_00412	lmo0723		6.1E+08	9.2E+07	1.5E-01	6.7E+00	2.9E-03
LMRG_00415	lmo0727	FlmS	2.1E+09	2.3E+09	1.1E+00	9.4E-01	2.5E-02
LMRG_00417	lmo0729		3.4E+06	0.0E+00	0.0E+00	#DIV/0!	1.2E-01
LMRG_00418	lmo0730		1.0E+07	1.3E+07	1.2E+00	8.3E-01	3.5E-01
LMRG_00422	lmo0734		1.8E+07	1.4E+07	8.0E-01	1.3E+00	1.6E-02
LMRG_00423	lmo0735	Rpe	0.0E+00	5.4E+05	#DIV/0!	0.0E+00	3.7E-01
LMRG_00424	lmo0736	RpiB	1.3E+07	1.3E+07	9.9E-01	1.0E+00	9.4E-01
LMRG_00426	lmo0738		7.9E+06	8.5E+06	1.1E+00	9.4E-01	6.4E-01
LMRG_00427	lmo0739		1.7E+07	1.8E+07	1.1E+00	9.2E-01	7.2E-01
LMRG_00428	lmo0740		1.2E+06	0.0E+00	0.0E+00	#DIV/0!	3.7E-01
LMRG_00430	lmo0742		0.0E+00	4.1E+06	#DIV/0!	0.0E+00	3.7E-01
LMRG_00431	lmo0743		0.0E+00	1.3E+06	#DIV/0!	0.0E+00	3.7E-01
LMRG_00447	lmo0759		1.6E+07	1.3E+07	8.5E-01	1.2E+00	3.8E-01
LMRG_00450	lmo0762	HflX	7.2E+06	0.0E+00	0.0E+00	#DIV/0!	2.9E-04
LMRG_00451	lmo0763		5.9E+06	0.0E+00	0.0E+00	#DIV/0!	1.2E-01
LMRG_00452	lmo0764	LplA2	7.4E+06	0.0E+00	0.0E+00	#DIV/0!	8.8E-05
LMRG_00456	lmo0768		4.9E+06	0.0E+00	0.0E+00	#DIV/0!	1.2E-01
LMRG_00458	lmo0770		2.7E+07	2.4E+07	8.8E-01	1.1E+00	1.3E-02
LMRG_00460	lmo0772		6.8E+06	1.7E+06	2.6E-01	3.9E+00	6.8E-02
LMRG_00461	lmo0773	YhfP	6.9E+07	8.1E+07	1.2E+00	8.5E-01	1.1E-01
LMRG_00462	lmo0774		1.5E+08	5.2E+07	3.4E-01	2.9E+00	2.8E-05
LMRG_00463	lmo0775		8.5E+06	0.0E+00	0.0E+00	#DIV/0!	2.9E-05
LMRG_00464	lmo0776		1.8E+06	0.0E+00	0.0E+00	#DIV/0!	3.7E-01
LMRG_00465	lmo0777		3.2E+07	3.6E+07	1.1E+00	8.9E-01	3.6E-01
LMRG_00467	lmo0779		0.0E+00	1.4E+06	#DIV/0!	0.0E+00	3.7E-01
LMRG_00469	lmo0781		9.8E+06	6.3E+06	6.4E-01	1.6E+00	4.1E-01
LMRG_00470	lmo0782		4.8E+06	2.4E+06	4.9E-01	2.0E+00	5.1E-01
LMRG_00472	lmo0784		2.7E+06	0.0E+00	0.0E+00	#DIV/0!	3.7E-01
LMRG_00473	lmo0785		1.8E+08	1.9E+08	1.0E+00	9.6E-01	5.4E-01
LMRG_00474	lmo0786	AzoR	8.4E+08	1.3E+09	1.6E+00	6.3E-01	3.1E-03
LMRG_00475	lmo0787		7.0E+06	4.9E+06	7.0E-01	1.4E+00	7.5E-01
LMRG_00476	lmo0788		1.6E+08	8.8E+07	5.5E-01	1.8E+00	4.2E-03
LMRG_00478	lmo0790	YbaK	3.9E+06	0.0E+00	0.0E+00	#DIV/0!	3.7E-01
LMRG_00479	lmo0791	SpoIIIAH	1.4E+08	3.9E+07	2.7E-01	3.7E+00	4.5E-05
LMRG_00480	lmo0792		2.3E+06	0.0E+00	0.0E+00	#DIV/0!	1.2E-01
LMRG_00484	lmo0796		2.0E+08	2.1E+08	1.0E+00	9.7E-01	7.6E-01
LMRG_00485	lmo0797		1.4E+07	2.1E+07	1.5E+00	6.6E-01	3.7E-01
LMRG_00489	lmo0800		2.7E+06	0.0E+00	0.0E+00	#DIV/0!	3.7E-01
LMRG_00491	lmo1030		2.0E+07	1.4E+07	6.8E-01	1.5E+00	1.3E-02
LMRG_00492	lmo1031		0.0E+00	3.0E+07	#DIV/0!	0.0E+00	1.1E-03
LMRG_00493	lmo1032		0.0E+00	9.8E+06	#DIV/0!	0.0E+00	1.1E-05
LMRG_00494	lmo1033		0.0E+00	2.2E+07	#DIV/0!	0.0E+00	9.0E-04
LMRG_00500	lmo1039		2.3E+06	0.0E+00	0.0E+00	#DIV/0!	3.7E-01
LMRG_00506	lmo1045	MoaD	6.6E+05	0.0E+00	0.0E+00	#DIV/0!	3.7E-01
LMRG_00509	lmo1048		1.3E+06	0.0E+00	0.0E+00	#DIV/0!	3.7E-01
LMRG_00512	lmo1051	Def	3.9E+08	4.1E+08	1.1E+00	9.4E-01	2.3E-01
LMRG_00514	lmo1052	PdhA	6.9E+08	9.4E+08	1.4E+00	7.3E-01	1.5E-02
LMRG_00515	lmo1053	PdhB	6.1E+08	9.9E+08	1.6E+00	6.2E-01	3.0E-04
LMRG_00516	lmo1054	PdhC	6.0E+08	9.0E+08	1.5E+00	6.6E-01	3.8E-03
LMRG_00517	lmo1055	PdhD	5.2E+08	6.5E+08	1.3E+00	8.0E-01	1.1E-02
LMRG_00518	lmo1056		5.5E+06	4.0E+06	7.3E-01	1.4E+00	5.1E-01
LMRG_00519	lmo1057		3.1E+06	0.0E+00	0.0E+00	#DIV/0!	3.7E-01
LMRG_00520	lmo1058		2.0E+07	2.8E+06	1.4E-01	7.2E+00	2.1E-02
LMRG_00521	lmo1059		3.6E+08	1.8E+08	5.0E-01	2.0E+00	2.3E-03
LMRG_00523	lmo1061		1.1E+08	3.7E+07	3.2E-01	3.1E+00	1.1E-01
LMRG_00526	lmo1064		4.8E+07	5.3E+07	1.1E+00	9.1E-01	4.2E-01
LMRG_00527	lmo1065		7.2E+07	2.2E+07	3.0E-01	3.3E+00	4.7E-04
LMRG_00528	lmo1066		1.4E+08	1.4E+08	1.0E+00	9.9E-01	9.0E-01
LMRG_00529	lmo1067	BipA	2.2E+09	2.5E+09	1.1E+00	8.8E-01	6.1E-02
LMRG_00530	lmo1068		1.2E+08	1.1E+08	8.9E-01	1.1E+00	2.3E-01
LMRG_00531	lmo1069		3.5E+07	3.5E+07	9.9E-01	1.0E+00	9.5E-01
LMRG_00533	lmo1071	FtsW1	0.0E+00	2.3E+06	#DIV/0!	0.0E+00	3.7E-01
LMRG_00534	lmo1072	PycA	1.4E+09	1.0E+09	7.5E-01	1.3E+00	8.1E-04
LMRG_00535	lmo1073		1.8E+08	1.7E+08	9.4E-01	1.1E+00	4.5E-01
LMRG_00536	lmo1074	TagG	9.9E+06	1.1E+07	1.1E+00	9.4E-01	9.4E-01
LMRG_00537	lmo1075	TagH	1.4E+08	1.3E+08	9.0E-01	1.1E+00	3.4E-01
LMRG_00538	lmo1076	Auto	3.2E+08	3.6E+08	1.1E+00	8.9E-01	2.4E-01
LMRG_00539	lmo1077		5.0E+08	5.1E+08	1.0E+00	9.9E-01	8.9E-01
LMRG_00540	lmo1078	GalU	3.2E+08	2.8E+08	8.7E-01	1.1E+00	3.7E-02
LMRG_00541	lmo1079	YfhO	1.1E+08	1.0E+08	9.1E-01	1.1E+00	1.8E-01
LMRG_00542	lmo1080	RmlT	7.1E+08	6.7E+08	9.4E-01	1.1E+00	4.6E-01
LMRG_00543	lmo1081	RmlA	4.9E+08	4.3E+08	8.8E-01	1.1E+00	4.3E-02
LMRG_00544	lmo1082	RmlC	4.3E+08	3.8E+08	8.8E-01	1.1E+00	7.6E-02

LMRG_00545	lmo1083	RmlB	5.3E+08	6.4E+08	1.2E+00	8.2E-01	1.6E-01
LMRG_00546	lmo1084	RmlD	3.9E+08	3.4E+08	8.6E-01	1.2E+00	2.6E-01
LMRG_00547	lmo1085		3.8E+07	2.1E+07	5.6E-01	1.8E+00	1.1E-01
LMRG_00548	lmo1086	TarI	1.0E+09	8.6E+08	8.3E-01	1.2E+00	4.6E-02
LMRG_00549	lmo1087	TarJ	7.1E+08	5.7E+08	8.0E-01	1.2E+00	5.5E-02
LMRG_00550	lmo1088	TagB	6.1E+06	4.9E+06	7.9E-01	1.3E+00	7.6E-01
LMRG_00551	lmo1089	TagD	1.1E+07	1.0E+07	9.4E-01	1.1E+00	6.4E-01
LMRG_00552	lmo1090		2.4E+07	2.1E+07	8.9E-01	1.1E+00	3.6E-01
LMRG_00553	lmo1091		2.9E+07	2.6E+07	8.8E-01	1.1E+00	2.6E-01
LMRG_00554	lmo1092		1.6E+08	1.1E+08	6.9E-01	1.5E+00	1.2E-03
LMRG_00555	lmo1093	NadE	9.6E+07	5.8E+07	6.0E-01	1.7E+00	4.3E-04
LMRG_00556	lmo1094		5.3E+07	2.7E+07	5.2E-01	1.9E+00	2.3E-03
LMRG_00557	lmo1095		7.2E+06	0.0E+00	0.0E+00	#DIV/0!	1.2E-01
LMRG_00558	lmo1096	GuaA	1.9E+09	2.4E+09	1.3E+00	7.8E-01	6.1E-04
LMRG_00560	lmo1117		2.1E+07	1.6E+07	7.8E-01	1.3E+00	7.3E-03
LMRG_00563	lmo1121		1.1E+07	0.0E+00	0.0E+00	#DIV/0!	2.1E-06
LMRG_00564	lmo1122		5.0E+05	0.0E+00	0.0E+00	#DIV/0!	3.7E-01
LMRG_00565	lmo1123		2.4E+06	0.0E+00	0.0E+00	#DIV/0!	3.7E-01
LMRG_00569	lmo1127		9.8E+05	0.0E+00	0.0E+00	#DIV/0!	3.7E-01
LMRG_00577	lmo1135		0.0E+00	5.1E+05	#DIV/0!	0.0E+00	3.7E-01
LMRG_00581	lmo1138	ClpP2	3.4E+08	4.1E+08	1.2E+00	8.3E-01	1.5E-01
LMRG_00586	lmo1143		0.0E+00	5.6E+05	#DIV/0!	0.0E+00	3.7E-01
LMRG_00597	lmo1154	PduD	2.6E+06	0.0E+00	0.0E+00	#DIV/0!	3.7E-01
LMRG_00617	lmo1171	PduQ	0.0E+00	2.7E+06	#DIV/0!	0.0E+00	3.7E-01
LMRG_00621	lmo1175	EutB	0.0E+00	2.7E+06	#DIV/0!	0.0E+00	3.7E-01
LMRG_00625	lmo1179		0.0E+00	3.3E+06	#DIV/0!	0.0E+00	3.7E-01
LMRG_00626	lmo1180		9.6E+06	2.5E+07	2.6E+00	3.8E-01	3.7E-02
LMRG_00633	lmo1187	EutQ	0.0E+00	1.3E+06	#DIV/0!	0.0E+00	3.7E-01
LMRG_00635	lmo1189		1.5E+06	1.5E+06	1.0E+00	9.8E-01	9.9E-01
LMRG_00645	lmo1199	CbiH	0.0E+00	1.2E+08	#DIV/0!	0.0E+00	1.4E-01
LMRG_00661	lmo1215		6.4E+06	1.0E+07	1.6E+00	6.2E-01	3.2E-02
LMRG_00663	lmo1217		2.9E+08	1.9E+08	6.7E-01	1.5E+00	2.8E-03
LMRG_00664	lmo1218		1.0E+08	8.8E+07	8.5E-01	1.2E+00	2.6E-01
LMRG_00665	lmo1219		2.7E+07	2.6E+07	9.7E-01	1.0E+00	7.7E-01
LMRG_00666	lmo1220		1.9E+07	2.0E+07	1.1E+00	9.5E-01	6.8E-01
LMRG_00667	lmo1221	PheS	3.4E+08	3.1E+08	9.2E-01	1.1E+00	2.0E-01
LMRG_00668	lmo1222	PheT	5.8E+08	5.9E+08	1.0E+00	9.9E-01	7.2E-01
LMRG_00669	lmo1223		2.6E+07	2.2E+07	8.4E-01	1.2E+00	2.8E-01
LMRG_00670	lmo1224		1.6E+07	1.5E+07	9.1E-01	1.1E+00	5.8E-01
LMRG_00671	lmo1225		4.3E+05	0.0E+00	0.0E+00	#DIV/0!	3.7E-01
LMRG_00672	lmo1226		6.0E+07	2.0E+06	3.3E-02	3.0E+01	7.6E-03
LMRG_00673	lmo1227	Ung2	4.4E+06	0.0E+00	0.0E+00	#DIV/0!	1.2E-01
LMRG_00674	lmo1228	RnhC	2.5E+07	3.7E+07	1.5E+00	6.7E-01	1.9E-02
LMRG_00675	lmo1229	ZapA	1.1E+08	1.2E+08	1.1E+00	9.1E-01	5.6E-01
LMRG_00677	lmo1231	PolX	9.5E+07	9.3E+07	9.7E-01	1.0E+00	1.3E-01
LMRG_00678	lmo1232	MutS2	2.1E+08	2.5E+08	1.2E+00	8.5E-01	9.9E-03
LMRG_00679	lmo1233	TrxA	2.9E+08	1.2E+08	4.1E-01	2.4E+00	1.4E-03
LMRG_00680	lmo1234	UvrC	1.8E+07	1.9E+07	1.0E+00	9.9E-01	9.1E-01
LMRG_00681	lmo1235		2.7E+08	2.8E+08	1.0E+00	9.5E-01	6.1E-01
LMRG_00682	lmo1236		8.8E+07	1.2E+08	1.4E+00	7.0E-01	3.8E-02
LMRG_00683	lmo1237	MurI	1.2E+08	1.6E+08	1.3E+00	7.5E-01	2.7E-03
LMRG_00684	lmo1238	Rph	1.4E+08	1.6E+08	1.2E+00	8.4E-01	1.0E-01
LMRG_00685	lmo1239		1.6E+08	1.6E+08	1.0E+00	9.8E-01	6.7E-01
LMRG_00686	lmo1240		1.3E+08	1.1E+08	8.8E-01	1.1E+00	1.5E-01
LMRG_00688	lmo1242		6.0E+07	3.3E+07	5.6E-01	1.8E+00	9.3E-03
LMRG_00691	lmo1245		4.3E+05	0.0E+00	0.0E+00	#DIV/0!	3.7E-01
LMRG_00692	lmo1246	DbpA	5.3E+07	4.3E+07	8.2E-01	1.2E+00	3.6E-02
LMRG_00695	lmo1249		0.0E+00	2.0E+07	#DIV/0!	0.0E+00	2.3E-03
LMRG_00697	lmo1251		5.2E+05	0.0E+00	0.0E+00	#DIV/0!	3.7E-01
LMRG_00699	lmo1252		0.0E+00	1.9E+06	#DIV/0!	0.0E+00	3.7E-01
LMRG_00701			9.5E+06	0.0E+00	0.0E+00	#DIV/0!	1.2E-01
LMRG_00702	lmo1253	TreR	2.2E+07	2.7E+07	1.2E+00	8.3E-01	9.6E-02
LMRG_00703	lmo1254	TreC	2.2E+08	1.7E+08	7.7E-01	1.3E+00	9.3E-03
LMRG_00704	lmo1255	TreP	8.7E+07	6.9E+07	7.8E-01	1.3E+00	7.8E-02
LMRG_00706	lmo1257		2.5E+08	1.7E+08	7.0E-01	1.4E+00	1.4E-02
LMRG_00707	lmo1258		1.4E+07	0.0E+00	0.0E+00	#DIV/0!	1.1E-04
LMRG_00708	lmo1259	ProA	2.5E+07	4.0E+07	1.6E+00	6.3E-01	1.4E-01
LMRG_00709	lmo1260	ProB	7.4E+07	8.7E+07	1.2E+00	8.5E-01	1.8E-01
LMRG_00710	lmo1261		4.1E+07	3.9E+07	9.7E-01	1.0E+00	8.4E-01
LMRG_00711	lmo1262		0.0E+00	7.1E+05	#DIV/0!	0.0E+00	3.7E-01
LMRG_00715	lmo1266		0.0E+00	1.3E+07	#DIV/0!	0.0E+00	5.9E-10
LMRG_00716	lmo1267	Tig	7.0E+09	8.4E+09	1.2E+00	8.4E-01	3.7E-03
LMRG_00718	lmo1268	ClpX	1.1E+09	1.1E+09	9.3E-01	1.1E+00	1.5E-01
LMRG_00719	lmo1269	SipX	3.4E+07	3.0E+07	8.7E-01	1.2E+00	1.8E-01
LMRG_00720	lmo1270	SipY	6.5E+06	3.1E+06	4.8E-01	2.1E+00	5.0E-01
LMRG_00721	lmo1271	SipZ	4.3E+07	4.5E+07	1.1E+00	9.4E-01	2.8E-01
LMRG_00722	lmo1272	RbgA	7.8E+07	8.9E+07	1.1E+00	8.8E-01	1.7E-01
LMRG_00723	lmo1273	RnhB	0.0E+00	1.8E+06	#DIV/0!	0.0E+00	3.7E-01
LMRG_00725	lmo1275	TopA	2.9E+08	2.3E+08	8.1E-01	1.2E+00	8.5E-02
LMRG_00726	lmo1276	TrmFO	2.4E+08	2.0E+08	8.5E-01	1.2E+00	2.5E-02
LMRG_00727	lmo1277	XerC	5.8E+05	0.0E+00	0.0E+00	#DIV/0!	3.7E-01
LMRG_00728	lmo1278	HslV	4.7E+07	4.5E+07	9.5E-01	1.1E+00	6.2E-01
LMRG_00729	lmo1279	HslU	3.3E+08	2.8E+08	8.5E-01	1.2E+00	1.5E-02
LMRG_00730	lmo1280	CodY	1.1E+09	1.2E+09	1.1E+00	9.0E-01	8.8E-02
LMRG_00731	lmo1281		6.4E+06	0.0E+00	0.0E+00	#DIV/0!	5.3E-05
LMRG_00733	lmo1283		2.4E+08	1.9E+08	7.7E-01	1.3E+00	7.1E-04

LMRG_00734	lmo1284	PlsY	0.0E+00	4.9E+06	#DIV/0!	0.0E+00	3.7E-01
LMRG_00735	lmo1285		6.2E+07	6.5E+07	1.0E+00	9.7E-01	7.8E-01
LMRG_00736	lmo1286	ParE	3.2E+08	3.0E+08	9.5E-01	1.1E+00	5.9E-01
LMRG_00737	lmo1287	ParC	6.2E+08	6.3E+08	1.0E+00	9.9E-01	7.8E-01
LMRG_00738	lmo1288	LuxS	2.5E+07	1.9E+07	7.9E-01	1.3E+00	2.9E-02
LMRG_00741	lmo1291	OatA	2.5E+07	1.6E+07	6.4E-01	1.6E+00	1.1E-02
LMRG_00743	lmo1293	GlpD	1.8E+07	7.6E+06	4.2E-01	2.4E+00	5.4E-02
LMRG_00744	lmo1294	MiaA	1.4E+07	2.0E+07	1.5E+00	6.8E-01	4.0E-02
LMRG_00745	lmo1295	Hfq	2.1E+05	0.0E+00	0.0E+00	#DIV/0!	3.7E-01
LMRG_00748	lmo1298	GlnR	3.0E+07	2.6E+07	8.7E-01	1.1E+00	5.1E-01
LMRG_00749	lmo1299	GlnA	1.2E+09	1.0E+09	8.2E-01	1.2E+00	7.2E-02
LMRG_00752	lmo1302	LexA	6.6E+07	7.6E+07	1.2E+00	8.6E-01	3.7E-01
LMRG_00754	lmo1304		3.8E+07	4.6E+07	1.2E+00	8.3E-01	6.1E-01
LMRG_00755	lmo1305	Tkt	2.0E+09	2.2E+09	1.1E+00	9.2E-01	4.2E-02
LMRG_00756	lmo1306		2.5E+08	3.5E+08	1.4E+00	7.3E-01	8.6E-02
LMRG_00758	lmo1308		3.0E+07	1.7E+07	5.6E-01	1.8E+00	2.3E-03
LMRG_00759	lmo1309		2.6E+06	0.0E+00	0.0E+00	#DIV/0!	3.7E-01
LMRG_00763	lmo1313	PyrH	5.1E+08	6.8E+08	1.3E+00	7.5E-01	6.2E-03
LMRG_00764	lmo1314	Frr	1.8E+09	1.9E+09	1.0E+00	9.7E-01	6.6E-01
LMRG_00765	lmo1315		3.2E+07	6.3E+07	2.0E+00	5.1E-01	6.3E-03
LMRG_00766	lmo1316	CdsA	0.0E+00	2.5E+06	#DIV/0!	0.0E+00	3.7E-01
LMRG_00767	lmo1317	Dxr	5.2E+07	5.2E+07	9.9E-01	1.0E+00	8.5E-01
LMRG_00768	lmo1318	RseP	3.7E+08	4.8E+08	1.3E+00	7.7E-01	1.7E-02
LMRG_00769	lmo1319	ProS	7.7E+08	6.1E+08	7.9E-01	1.3E+00	2.0E-02
LMRG_00770	lmo1320	PolC	1.4E+08	1.4E+08	1.0E+00	9.8E-01	6.3E-01
LMRG_00771	lmo1321	RimP	1.4E+08	1.5E+08	1.1E+00	9.1E-01	4.4E-01
LMRG_00772	lmo1322	NusA	1.5E+09	1.5E+09	1.0E+00	1.0E+00	9.8E-01
LMRG_00773	lmo1323		1.1E+08	1.0E+08	9.6E-01	1.0E+00	6.3E-01
LMRG_00774	lmo1324		2.7E+07	2.8E+07	1.0E+00	9.8E-01	9.0E-01
LMRG_00775	lmo1325	InfB	1.6E+09	1.7E+09	1.1E+00	9.4E-01	3.4E-01
LMRG_00776	lmo1326		4.0E+05	0.0E+00	0.0E+00	#DIV/0!	3.7E-01
LMRG_00777	lmo1327	RbfA	7.7E+08	9.0E+08	1.2E+00	8.5E-01	3.7E-01
LMRG_00778	lmo1328	TruB	6.8E+07	6.1E+07	9.0E-01	1.1E+00	1.8E-01
LMRG_00779	lmo1329	RibC	1.4E+08	1.1E+08	7.7E-01	1.3E+00	1.8E-03
LMRG_00780	lmo1330	RpsO	2.7E+09	2.5E+09	9.4E-01	1.1E+00	5.4E-01
LMRG_00781	lmo1331	Pnp	2.3E+09	2.1E+09	9.1E-01	1.1E+00	5.2E-02
LMRG_00783	lmo1333		3.5E+08	4.8E+08	1.4E+00	7.3E-01	1.1E-02
LMRG_00784	lmo1334		2.1E+08	3.2E+08	1.5E+00	6.6E-01	1.8E-02
LMRG_00785	lmo1335	RpmG	8.0E+08	8.2E+08	1.0E+00	9.8E-01	8.1E-01
LMRG_00786	lmo1336		7.1E+06	1.8E+06	2.5E-01	4.0E+00	4.0E-02
LMRG_00787	lmo1337		2.5E+07	3.5E+07	1.4E+00	7.2E-01	3.3E-03
LMRG_00788	lmo1338		0.0E+00	3.0E+06	#DIV/0!	0.0E+00	3.7E-01
LMRG_00789	lmo1339		5.9E+08	5.5E+08	9.3E-01	1.1E+00	2.8E-01
LMRG_00790	lmo1340		0.0E+00	1.8E+06	#DIV/0!	0.0E+00	3.7E-01
LMRG_00798	lmo1348	GcvT	3.6E+06	0.0E+00	0.0E+00	#DIV/0!	1.3E-01
LMRG_00799	lmo1349	GcvPA	0.0E+00	7.7E+05	#DIV/0!	0.0E+00	3.7E-01
LMRG_00800	lmo1350	GcvPB	8.2E+06	0.0E+00	0.0E+00	#DIV/0!	1.4E-04
LMRG_00801	lmo1351		5.0E+08	6.2E+08	1.2E+00	8.0E-01	1.3E-02
LMRG_00803	lmo1353		4.0E+07	4.8E+07	1.2E+00	8.4E-01	7.7E-02
LMRG_00804	lmo1354		4.0E+08	1.7E+08	4.3E-01	2.3E+00	1.3E-03
LMRG_00805	lmo1355	Efp	2.2E+08	3.1E+08	1.4E+00	7.3E-01	5.4E-04
LMRG_00806	lmo1356	AccB	5.5E+07	6.1E+07	1.1E+00	8.9E-01	6.5E-01
LMRG_00807	lmo1357	AccC	7.0E+08	5.7E+08	8.1E-01	1.2E+00	1.2E-01
LMRG_00808	lmo1358		4.3E+07	2.7E+07	6.4E-01	1.6E+00	4.6E-02
LMRG_00809	lmo1359	NusB	1.6E+08	1.4E+08	8.7E-01	1.1E+00	3.8E-01
LMRG_00810	lmo1360	FolD	2.0E+08	1.1E+08	5.3E-01	1.9E+00	2.3E-03
LMRG_00811	lmo1361	XseA	1.2E+07	3.8E+06	3.1E-01	3.3E+00	3.5E-01
LMRG_00812	lmo1362	XseB	2.3E+07	1.1E+07	4.6E-01	2.2E+00	8.1E-02
LMRG_00813	lmo1363		3.0E+07	2.1E+07	7.2E-01	1.4E+00	4.5E-03
LMRG_00814	lmo1364	CspL	3.8E+09	4.7E+09	1.3E+00	7.9E-01	6.1E-02
LMRG_00815	lmo1365	Dxs	2.3E+07	2.2E+07	9.6E-01	1.0E+00	8.0E-01
LMRG_00816	lmo1366		2.8E+07	3.4E+07	1.2E+00	8.2E-01	2.4E-01
LMRG_00817	lmo1367	ArgR	1.2E+08	7.4E+07	6.3E-01	1.6E+00	3.7E-02
LMRG_00818	lmo1368	RecN	4.6E+08	3.1E+08	6.6E-01	1.5E+00	3.0E-02
LMRG_00819	lmo1369		5.8E+07	3.2E+07	5.5E-01	1.8E+00	2.5E-02
LMRG_00820	lmo1370	Buk	3.4E+07	0.0E+00	0.0E+00	#DIV/0!	3.9E-05
LMRG_00821	lmo1371	LpdA	2.6E+06	0.0E+00	0.0E+00	#DIV/0!	3.7E-01
LMRG_00822	lmo1372		2.9E+08	1.5E+08	5.0E-01	2.0E+00	3.9E-04
LMRG_00823	lmo1373		4.6E+08	2.7E+08	6.0E-01	1.7E+00	3.3E-04
LMRG_00824	lmo1374		2.6E+08	1.4E+08	5.2E-01	1.9E+00	4.6E-05
LMRG_00828	lmo1376	GndA	3.0E+09	3.4E+09	1.1E+00	9.0E-01	5.7E-03
LMRG_00829	lmo1377	LisR	1.1E+08	5.4E+07	5.1E-01	2.0E+00	2.9E-04
LMRG_00830	lmo1378	LisK	4.7E+07	1.9E+07	4.0E-01	2.5E+00	4.9E-02
LMRG_00831	lmo1379	YqjG	8.2E+07	1.2E+08	1.4E+00	7.1E-01	1.4E-01
LMRG_00833	lmo1381		5.2E+07	0.0E+00	0.0E+00	#DIV/0!	9.0E-06
LMRG_00834	lmo1382		4.9E+07	0.0E+00	0.0E+00	#DIV/0!	1.5E-04
LMRG_00835	lmo1383	Fni	3.4E+07	1.9E+07	5.5E-01	1.8E+00	8.5E-05
LMRG_00836	lmo1384	TrhO	3.2E+06	3.1E+06	9.4E-01	1.1E+00	9.4E-01
LMRG_00837	lmo1385		1.6E+06	0.0E+00	0.0E+00	#DIV/0!	1.2E-01
LMRG_00838	lmo1386	FtsK	1.4E+08	1.4E+08	9.7E-01	1.0E+00	6.3E-01
LMRG_00839	lmo1387	ProG	6.4E+05	0.0E+00	0.0E+00	#DIV/0!	3.7E-01
LMRG_00840	lmo1388	TcsA	5.6E+09	3.2E+09	5.8E-01	1.7E+00	3.1E-04
LMRG_00841	lmo1389		4.5E+08	3.8E+08	8.4E-01	1.2E+00	2.9E-02
LMRG_00843	lmo1391		4.3E+07	4.5E+07	1.0E+00	9.5E-01	7.7E-01
LMRG_00844	lmo1392		8.6E+07	3.6E+07	4.2E-01	2.4E+00	1.5E-03
LMRG_00845	lmo1393		1.7E+08	8.6E+07	5.0E-01	2.0E+00	2.1E-04

LMRG_00846	lmo1394		0.0E+00	3.4E+05	#DIV/0!	0.0E+00	3.7E-01
LMRG_00847	lmo1395	RodZ	3.2E+08	3.3E+08	1.0E+00	9.7E-01	8.1E-01
LMRG_00848	lmo1396	PgsA	1.8E+07	1.7E+07	9.4E-01	1.1E+00	5.7E-01
LMRG_00849	lmo1397	CinA	4.1E+07	3.9E+07	9.6E-01	1.0E+00	6.7E-01
LMRG_00850	lmo1398	RecA	6.8E+08	1.1E+09	1.6E+00	6.3E-01	2.5E-04
LMRG_00851	lmo1399	Rny	4.8E+08	4.9E+08	1.0E+00	9.6E-01	4.5E-01
LMRG_00852	lmo1400		1.1E+07	2.3E+06	2.1E-01	4.7E+00	2.3E-02
LMRG_00853	lmo1401		3.2E+08	3.5E+08	1.1E+00	9.2E-01	2.3E-01
LMRG_00854	lmo1402		2.5E+08	1.7E+08	6.9E-01	1.4E+00	3.6E-04
LMRG_00855	lmo1403	MutS	3.5E+08	3.5E+08	1.0E+00	9.9E-01	8.1E-01
LMRG_00856	lmo1404	MutL	9.2E+07	9.1E+07	9.9E-01	1.0E+00	9.2E-01
LMRG_00857	lmo1405		3.1E+07	2.1E+07	6.9E-01	1.5E+00	2.9E-01
LMRG_00858	lmo1406	PflB	3.7E+09	3.7E+09	1.0E+00	9.9E-01	9.3E-01
LMRG_00859	lmo1407	PflC	1.5E+08	1.4E+08	9.2E-01	1.1E+00	2.4E-01
LMRG_00860	lmo1408	LadR	1.5E+07	1.1E+07	7.2E-01	1.4E+00	8.4E-03
LMRG_00866	lmo1414	AtoB	3.0E+08	2.8E+08	9.2E-01	1.1E+00	9.5E-02
LMRG_00867	lmo1415	HmgS	4.8E+08	5.5E+08	1.1E+00	8.7E-01	4.8E-02
LMRG_00872	lmo1420	MurB	7.8E+07	5.5E+07	7.1E-01	1.4E+00	5.6E-02
LMRG_00873	lmo1421		1.4E+07	2.5E+07	1.8E+00	5.4E-01	2.0E-03
LMRG_00874	lmo1422		1.3E+07	2.8E+07	2.1E+00	4.8E-01	5.3E-03
LMRG_00875	lmo1423		1.4E+08	1.2E+08	8.3E-01	1.2E+00	6.3E-02
LMRG_00876	lmo1424	MntH	1.1E+07	3.3E+07	2.9E+00	3.4E-01	5.7E-03
LMRG_00878	lmo1426	OpuCC	2.4E+07	3.1E+07	1.3E+00	7.7E-01	5.4E-02
LMRG_00879	lmo1427	OpuCB	0.0E+00	1.5E+06	#DIV/0!	0.0E+00	3.7E-01
LMRG_00880	lmo1428	OpuCA	1.8E+08	2.7E+08	1.5E+00	6.7E-01	1.8E-03
LMRG_00883	lmo1431		7.8E+08	9.3E+08	1.2E+00	8.4E-01	3.8E-02
LMRG_00884	lmo1432		1.1E+07	0.0E+00	0.0E+00	#DIV/0!	1.3E-01
LMRG_00885	lmo1433		0.0E+00	1.3E+06	#DIV/0!	0.0E+00	3.7E-01
LMRG_00886	lmo1434	Rnj	5.2E+08	4.4E+08	8.4E-01	1.2E+00	2.1E-03
LMRG_00887	lmo1435	DapA	3.5E+08	2.7E+08	7.8E-01	1.3E+00	1.7E-02
LMRG_00888	lmo1436	DapG	2.2E+08	2.0E+08	8.9E-01	1.1E+00	9.0E-02
LMRG_00889	lmo1437	Asd	6.1E+08	4.3E+08	7.0E-01	1.4E+00	5.5E-03
LMRG_00890	lmo1438	PbpB1	3.2E+08	5.4E+08	1.7E+00	6.0E-01	5.7E-05
LMRG_00891	lmo1439	SodA	9.3E+08	3.3E+08	3.5E-01	2.8E+00	7.1E-04
LMRG_00892	lmo1440		7.3E+07	5.1E+07	7.0E-01	1.4E+00	1.4E-02
LMRG_00897	lmo1445	ZurR	1.8E+07	1.5E+07	8.6E-01	1.2E+00	2.2E-01
LMRG_00899	lmo1447	ZurA	1.3E+07	8.7E+06	6.9E-01	1.4E+00	6.0E-02
LMRG_00900	lmo1448	PpaC	1.5E+09	1.6E+09	1.1E+00	9.4E-01	3.5E-01
LMRG_00901	lmo1449	Nfo	1.4E+08	1.7E+08	1.2E+00	8.3E-01	5.2E-02
LMRG_00902	lmo1450	CshB	3.6E+08	4.6E+08	1.3E+00	7.8E-01	2.4E-02
LMRG_00904	lmo1452		1.4E+08	1.3E+08	9.6E-01	1.0E+00	7.2E-01
LMRG_00905	lmo1453		1.2E+08	1.0E+08	8.0E-01	1.2E+00	1.8E-02
LMRG_00906	lmo1454	SigA	4.4E+08	5.3E+08	1.2E+00	8.5E-01	3.0E-02
LMRG_00907	lmo1455	DnaG	7.5E+07	7.7E+07	1.0E+00	9.6E-01	1.9E-01
LMRG_00909	lmo1457		8.2E+07	5.5E+07	6.7E-01	1.5E+00	6.9E-03
LMRG_00910	lmo1458	GlyS	5.6E+08	6.2E+08	1.1E+00	9.2E-01	4.7E-02
LMRG_00911	lmo1459	GlyQ	2.1E+08	2.3E+08	1.1E+00	9.1E-01	3.0E-01
LMRG_00914	lmo1462	Era	2.0E+08	1.5E+08	7.2E-01	1.4E+00	2.4E-02
LMRG_00915	lmo1463		1.1E+08	5.2E+07	4.5E-01	2.2E+00	4.4E-04
LMRG_00916	lmo1464		6.6E+05	0.0E+00	0.0E+00	#DIV/0!	3.7E-01
LMRG_00917	lmo1465	YbeY	2.3E+07	1.2E+07	5.3E-01	1.9E+00	2.1E-01
LMRG_00918	lmo1466	PgpH	2.7E+07	1.2E+07	4.7E-01	2.1E+00	2.1E-02
LMRG_00919	lmo1467	PhoH	6.6E+07	3.5E+07	5.4E-01	1.9E+00	7.2E-04
LMRG_00921	lmo1468		3.4E+08	3.9E+08	1.2E+00	8.6E-01	6.6E-02
LMRG_00922	lmo1469	RpsU	2.7E+08	7.9E+08	2.9E+00	3.4E-01	2.9E-03
LMRG_00923	lmo1470		5.3E+07	6.0E+07	1.1E+00	8.9E-01	3.9E-01
LMRG_00924	lmo1471	PrmA	1.6E+08	2.4E+08	1.4E+00	6.9E-01	9.0E-04
LMRG_00925	lmo1472	DnaJ	6.8E+08	8.5E+08	1.3E+00	7.9E-01	2.2E-01
LMRG_00926	lmo1473	DnaK	1.3E+10	1.7E+10	1.3E+00	7.8E-01	4.7E-02
LMRG_00927	lmo1474	GrpE	9.1E+08	1.5E+09	1.6E+00	6.2E-01	2.7E-02
LMRG_00928	lmo1475	HrcA	2.7E+07	8.9E+07	3.3E+00	3.1E-01	3.4E-03
LMRG_00929	lmo1476	HemN	1.9E+07	2.1E+07	1.1E+00	8.9E-01	2.0E-01
LMRG_00932	lmo1479	LepA	2.7E+08	3.1E+08	1.1E+00	9.0E-01	1.6E-01
LMRG_00933	lmo1480	RpsT	1.4E+09	2.2E+09	1.6E+00	6.3E-01	3.7E-03
LMRG_00934	lmo1481	HolA	2.1E+07	1.7E+07	8.2E-01	1.2E+00	2.4E-01
LMRG_00936	lmo1483	ComEB	5.3E+07	4.5E+07	8.6E-01	1.2E+00	2.7E-01
LMRG_00938	lmo1485		0.0E+00	9.3E+05	#DIV/0!	0.0E+00	3.7E-01
LMRG_00939	lmo1486	RsfS	2.7E+08	3.6E+08	1.3E+00	7.4E-01	2.9E-02
LMRG_00940	lmo1487	YqeK	5.3E+07	6.1E+07	1.2E+00	8.6E-01	2.9E-01
LMRG_00941	lmo1488	NadD	8.7E+06	1.1E+07	1.2E+00	8.1E-01	2.2E-01
LMRG_00942	lmo1489	YhbY	2.3E+08	2.3E+08	9.7E-01	1.0E+00	7.8E-01
LMRG_00943	lmo1490	AroE	7.9E+06	8.0E+06	1.0E+00	9.9E-01	9.4E-01
LMRG_00944	lmo1491	YqeH	2.1E+08	2.6E+08	1.3E+00	7.9E-01	2.7E-02
LMRG_00945	lmo1492		4.1E+07	4.6E+07	1.1E+00	8.9E-01	5.1E-01
LMRG_00946	lmo1493		5.8E+08	5.2E+08	9.0E-01	1.1E+00	1.8E-01
LMRG_00948	lmo1801	Ffh	3.9E+08	3.8E+08	9.9E-01	1.0E+00	7.9E-01
LMRG_00949	lmo1802		1.9E+06	2.0E+06	1.1E+00	9.3E-01	9.6E-01
LMRG_00950	lmo1803	FtsY	3.5E+08	3.7E+08	1.1E+00	9.4E-01	5.7E-03
LMRG_00951	lmo1804	Smc	2.9E+08	3.1E+08	1.0E+00	9.6E-01	3.7E-01
LMRG_00952	lmo1805	Rnc	7.6E+07	7.8E+07	1.0E+00	9.7E-01	6.5E-01
LMRG_00953	lmo1806	AcpP	1.3E+09	1.2E+09	8.7E-01	1.1E+00	2.6E-01
LMRG_00954	lmo1807	FabG	1.3E+09	1.1E+09	8.7E-01	1.2E+00	1.8E-01
LMRG_00955	lmo1808	FabD	1.1E+09	1.0E+09	9.4E-01	1.1E+00	2.8E-01
LMRG_00956	lmo1809	PlsX	4.6E+08	3.9E+08	8.4E-01	1.2E+00	2.9E-02
LMRG_00957	lmo1810	FapR	4.2E+07	3.3E+07	7.9E-01	1.3E+00	1.1E-01
LMRG_00959	lmo1812	SdaAA	8.9E+07	7.3E+07	8.2E-01	1.2E+00	3.2E-02

LMRG_00960	lmo1813		1.8E+07	1.4E+07	7.8E-01	1.3E+00	1.0E-01
LMRG_00961	lmo1814		5.3E+08	5.9E+08	1.1E+00	8.9E-01	4.3E-02
LMRG_00962	lmo1815		9.9E+07	1.0E+08	1.0E+00	9.7E-01	7.0E-01
LMRG_00963	lmo1816	RpmB	1.9E+08	4.4E+08	2.3E+00	4.3E-01	7.7E-02
LMRG_00964	lmo1817		2.9E+07	2.0E+07	6.8E-01	1.5E+00	1.2E-02
LMRG_00965	lmo1818	Rpe	6.4E+07	5.1E+07	8.0E-01	1.3E+00	9.0E-02
LMRG_00966	lmo1819	RsgA	2.5E+07	2.0E+07	7.9E-01	1.3E+00	3.5E-02
LMRG_00967	lmo1820	PrkA	2.4E+08	2.2E+08	9.3E-01	1.1E+00	4.0E-01
LMRG_00968	lmo1821	PrpC	6.0E+07	6.3E+07	1.0E+00	9.5E-01	6.9E-01
LMRG_00969	lmo1822	RsmB	3.3E+08	3.9E+08	1.2E+00	8.5E-01	1.3E-02
LMRG_00970	lmo1823	Fmt	2.3E+08	2.5E+08	1.1E+00	9.1E-01	3.1E-01
LMRG_00971	lmo1824	PriA	7.9E+07	7.8E+07	9.9E-01	1.0E+00	8.9E-01
LMRG_00972	lmo1825	CoaBC	2.4E+08	2.4E+08	1.0E+00	9.9E-01	9.5E-01
LMRG_00973	lmo1826	RpoZ	6.5E+08	6.4E+08	9.7E-01	1.0E+00	7.2E-01
LMRG_00974	lmo1827	Gmk	1.3E+08	1.5E+08	1.2E+00	8.5E-01	7.3E-02
LMRG_00976	lmo1829	RqcH	6.7E+07	3.9E+07	5.9E-01	1.7E+00	2.9E-04
LMRG_00977	lmo1830		2.1E+07	3.2E+07	1.5E+00	6.7E-01	1.7E-02
LMRG_00978	lmo1831	PyrE	2.6E+07	2.1E+07	8.1E-01	1.2E+00	5.8E-02
LMRG_00979	lmo1832	PyrF	7.8E+05	0.0E+00	0.0E+00	#DIV/0!	3.7E-01
LMRG_00981	lmo1834	PyrK	0.0E+00	2.4E+05	#DIV/0!	0.0E+00	3.7E-01
LMRG_00982	lmo1835	CarB	4.4E+07	5.4E+07	1.2E+00	8.1E-01	2.7E-01
LMRG_00983	lmo1836	CarA	2.2E+06	0.0E+00	0.0E+00	#DIV/0!	3.7E-01
LMRG_00984	lmo1837	PyrC	0.0E+00	7.1E+06	#DIV/0!	0.0E+00	3.7E-01
LMRG_00985	lmo1838	PyrB	0.0E+00	8.9E+05	#DIV/0!	0.0E+00	3.7E-01
LMRG_00986	lmo1839	PyrP	0.0E+00	2.5E+06	#DIV/0!	0.0E+00	3.7E-01
LMRG_00987	lmo1840	PyrR	4.2E+07	3.3E+07	7.9E-01	1.3E+00	1.8E-01
LMRG_00990	lmo1843		6.7E+07	7.3E+07	1.1E+00	9.2E-01	3.4E-01
LMRG_00991	lmo1844	LspA	0.0E+00	2.4E+05	#DIV/0!	0.0E+00	3.7E-01
LMRG_00994	lmo1847	MntA	6.8E+07	2.2E+08	3.3E+00	3.1E-01	4.8E-06
LMRG_00995	lmo1848	MntC	0.0E+00	2.6E+06	#DIV/0!	0.0E+00	3.7E-01
LMRG_00996	lmo1849	MntB	6.6E+07	1.7E+08	2.6E+00	3.8E-01	1.3E-04
LMRG_00997	lmo1850		9.1E+07	9.5E+07	1.0E+00	9.7E-01	6.0E-01
LMRG_00998	lmo1851		1.6E+08	2.0E+08	1.3E+00	8.0E-01	1.7E-02
LMRG_00999	lmo1852	CopZ	5.7E+07	4.8E+07	8.5E-01	1.2E+00	2.0E-01
LMRG_01000	lmo1853		7.4E+07	8.6E+07	1.2E+00	8.6E-01	3.4E-01
LMRG_01001	lmo1854	CsoR	1.7E+07	1.9E+07	1.1E+00	9.0E-01	2.3E-01
LMRG_01002	lmo1855	PbpD3	1.0E+08	1.3E+08	1.3E+00	7.6E-01	3.2E-02
LMRG_01003	lmo1856	DeoD	3.5E+08	3.5E+08	9.9E-01	1.0E+00	8.2E-01
LMRG_01004	lmo1857		1.6E+06	1.6E+06	1.0E+00	9.7E-01	9.8E-01
LMRG_01005	lmo1858		6.9E+07	7.9E+07	1.1E+00	8.8E-01	3.9E-01
LMRG_01007	lmo1860	MsrA	9.1E+07	6.2E+07	6.8E-01	1.5E+00	1.2E-02
LMRG_01008	lmo1861		2.9E+07	3.7E+07	1.3E+00	7.9E-01	6.0E-02
LMRG_01009	lmo1862		5.1E+07	7.4E+07	1.4E+00	6.9E-01	7.7E-03
LMRG_01010	lmo1863		1.6E+08	1.5E+08	9.4E-01	1.1E+00	3.6E-01
LMRG_01012	lmo1865		2.0E+06	2.6E+06	1.3E+00	7.8E-01	8.7E-01
LMRG_01013	lmo1866		3.4E+07	3.5E+07	1.1E+00	9.5E-01	6.7E-01
LMRG_01014	lmo1867	PpdK	0.0E+00	3.0E+05	#DIV/0!	0.0E+00	3.7E-01
LMRG_01015	lmo1868		1.4E+07	2.4E+06	1.7E-01	6.0E+00	2.7E-02
LMRG_01016	lmo1869		1.1E+07	0.0E+00	0.0E+00	#DIV/0!	5.1E-04
LMRG_01018	lmo1871		1.7E+08	1.7E+08	1.0E+00	9.9E-01	9.3E-01
LMRG_01020	lmo1873		2.6E+07	2.3E+07	8.9E-01	1.1E+00	2.5E-01
LMRG_01021	lmo1874	ThyA	1.3E+08	1.1E+08	8.5E-01	1.2E+00	9.3E-03
LMRG_01022	lmo1875		7.0E+07	6.3E+07	9.0E-01	1.1E+00	7.3E-02
LMRG_01023	lmo01876	Fhs	2.7E+08	5.0E+07	1.9E-01	5.4E+00	5.0E-06
LMRG_01024	lmo1878	MntR	6.4E+07	2.8E+06	4.3E-02	2.3E+01	7.3E-05
LMRG_01026	lmo1879	CspD	9.3E+06	1.2E+07	1.3E+00	7.9E-01	6.4E-01
LMRG_01027	lmo1880		1.2E+07	0.0E+00	0.0E+00	#DIV/0!	2.3E-04
LMRG_01028	lmo1881		4.1E+07	4.3E+07	1.1E+00	9.5E-01	7.3E-01
LMRG_01032	lmo1885	Xpt	1.3E+08	1.5E+08	1.2E+00	8.7E-01	3.2E-01
LMRG_01033	lmo1886		2.9E+08	2.8E+08	9.8E-01	1.0E+00	8.3E-01
LMRG_01034	lmo1887		9.0E+07	9.5E+07	1.1E+00	9.5E-01	6.1E-01
LMRG_01035	lmo1888	GpsB	7.4E+08	7.7E+08	1.0E+00	9.7E-01	7.2E-01
LMRG_01037	lmo1890		6.6E+06	0.0E+00	0.0E+00	#DIV/0!	3.7E-01
LMRG_01039	lmo1892	PbpA1	2.3E+08	3.1E+08	1.3E+00	7.5E-01	4.9E-03
LMRG_01040	lmo1893		5.0E+07	9.9E+07	2.0E+00	5.0E-01	5.2E-01
LMRG_01041	lmo1894	Nth	2.5E+07	2.2E+07	8.9E-01	1.1E+00	4.3E-02
LMRG_01042	lmo1895	DnaD	3.2E+07	5.0E+07	1.6E+00	6.4E-01	5.7E-03
LMRG_01043	lmo1896	AsnS	1.1E+09	1.2E+09	1.1E+00	9.4E-01	2.5E-01
LMRG_01044	lmo1897	AspB	3.9E+08	3.5E+08	9.0E-01	1.1E+00	6.6E-03
LMRG_01045	lmo1898		6.2E+07	6.1E+07	9.9E-01	1.0E+00	9.1E-01
LMRG_01046	lmo1899	DinG	2.2E+07	9.6E+06	4.4E-01	2.3E+00	4.5E-01
LMRG_01047	lmo1900	PanD	0.0E+00	7.6E+06	#DIV/0!	0.0E+00	6.9E-04
LMRG_01048	lmo1901	PanC	8.7E+07	3.2E+08	3.6E+00	2.7E-01	6.9E-05
LMRG_01049	lmo1902	PanB	2.8E+08	1.6E+09	5.6E+00	1.8E-01	4.1E-06
LMRG_01050	lmo1903		9.3E+06	8.4E+06	9.1E-01	1.1E+00	5.3E-01
LMRG_01051	lmo1904	BirA	6.2E+06	8.1E+06	1.3E+00	7.6E-01	5.8E-01
LMRG_01052	lmo1905	Cca	8.6E+05	1.9E+06	2.3E+00	4.4E-01	4.6E-01
LMRG_01053	lmo1906	MgsA	1.5E+06	3.0E+06	2.0E+00	5.1E-01	5.3E-01
LMRG_01054	lmo1907	DapB	1.7E+08	1.8E+08	1.0E+00	9.7E-01	5.6E-01
LMRG_01055	lmo1908		3.7E+06	0.0E+00	0.0E+00	#DIV/0!	3.7E-01
LMRG_01057	lmo1910		8.1E+05	0.0E+00	0.0E+00	#DIV/0!	3.7E-01
LMRG_01059	lmo1912	DgcB	0.0E+00	2.5E+05	#DIV/0!	0.0E+00	3.7E-01
LMRG_01061	lmo1914	PdeC	3.0E+06	3.0E+06	9.8E-01	1.0E+00	9.8E-01
LMRG_01062	lmo1915		1.0E+08	8.3E+07	8.0E-01	1.2E+00	3.3E-02
LMRG_01063	lmo1916	PbpC2	5.3E+06	2.7E+06	5.1E-01	2.0E+00	1.6E-01
LMRG_01064	lmo1917	PflA	2.3E+09	2.5E+09	1.1E+00	9.2E-01	5.6E-02

LMRG_01065	lmo1918		4.5E+07	5.2E+07	1.2E+00	8.5E-01	2.5E-02
LMRG_01066	lmo1919		1.5E+08	2.1E+08	1.4E+00	7.2E-01	3.7E-02
LMRG_01068	lmo1921	ReoY	1.7E+07	3.1E+07	1.9E+00	5.3E-01	1.2E-03
LMRG_01069	lmo1922		1.3E+08	1.0E+08	8.1E-01	1.2E+00	8.2E-02
LMRG_01070	lmo1923	AroA	1.9E+08	1.6E+08	8.6E-01	1.2E+00	1.9E-01
LMRG_01071	lmo1924	TyrA	3.6E+07	3.6E+07	9.9E-01	1.0E+00	8.8E-01
LMRG_01072	lmo1925	HisC	5.0E+07	3.5E+07	7.1E-01	1.4E+00	3.9E-06
LMRG_01073	lmo1926	AroH	1.0E+07	4.1E+06	4.1E-01	2.4E+00	4.2E-01
LMRG_01074	lmo1927	AroB	2.4E+07	2.2E+07	9.1E-01	1.1E+00	1.3E-01
LMRG_01075	lmo1928	AroC	2.3E+08	2.0E+08	8.8E-01	1.1E+00	1.2E-03
LMRG_01076	lmo1929	Ndk	1.4E+08	7.1E+07	5.2E-01	1.9E+00	9.0E-03
LMRG_01077	lmo1930	HepT	6.4E+07	5.4E+07	8.5E-01	1.2E+00	1.4E-01
LMRG_01078	lmo1931	MenG	1.9E+07	1.5E+07	8.3E-01	1.2E+00	3.0E-01
LMRG_01079	lmo1932		1.1E+07	8.3E+06	7.6E-01	1.3E+00	8.5E-02
LMRG_01080	lmo1933	FolE	7.4E+07	2.9E+07	3.9E-01	2.5E+00	1.4E-03
LMRG_01081	lmo1934	Hup	1.3E+10	7.1E+09	5.4E-01	1.8E+00	1.3E-04
LMRG_01082	lmo1935		8.7E+07	3.7E+07	4.2E-01	2.4E+00	2.6E-04
LMRG_01083	lmo1936	GpsA	5.6E+08	6.2E+08	1.1E+00	9.0E-01	1.6E-01
LMRG_01084	lmo1937	Der	4.9E+08	6.2E+08	1.3E+00	7.8E-01	2.6E-02
LMRG_01085	lmo1938	RpsA	1.9E+09	2.4E+09	1.3E+00	7.8E-01	9.1E-03
LMRG_01086	lmo1939	Cmk	2.7E+08	3.1E+08	1.2E+00	8.7E-01	3.2E-02
LMRG_01087	lmo1940		1.9E+07	2.2E+07	1.2E+00	8.5E-01	3.5E-01
LMRG_01088	lmo1941		1.4E+08	1.4E+08	1.0E+00	9.9E-01	8.9E-01
LMRG_01093	lmo1946		9.3E+07	6.6E+07	7.1E-01	1.4E+00	4.8E-02
LMRG_01094	lmo1947	ResE	2.4E+07	1.5E+07	6.0E-01	1.7E+00	5.0E-02
LMRG_01095	lmo1948	ResD	2.8E+08	1.6E+08	5.6E-01	1.8E+00	5.4E-04
LMRG_01096	lmo1949		1.6E+08	1.6E+08	9.8E-01	1.0E+00	7.9E-01
LMRG_01097	lmo1950	ScpB	2.8E+07	3.2E+07	1.2E+00	8.6E-01	4.6E-02
LMRG_01098	lmo1951	ScpA	7.7E+06	5.3E+06	7.0E-01	1.4E+00	4.4E-01
LMRG_01099	lmo1952	LysA	7.3E+07	6.9E+07	9.4E-01	1.1E+00	3.8E-01
LMRG_01100	lmo1953	Pnp	8.6E+08	1.0E+09	1.2E+00	8.7E-01	1.4E-01
LMRG_01101	lmo1954	DeoB	1.2E+09	1.4E+09	1.2E+00	8.6E-01	1.7E-02
LMRG_01102	lmo1955	XerD	3.3E+07	5.1E+07	1.5E+00	6.6E-01	4.1E-04
LMRG_01103	lmo1956	Fur	9.0E+07	6.4E+07	7.1E-01	1.4E+00	5.0E-02
LMRG_01106	lmo1959	FhuD	2.3E+06	0.0E+00	0.0E+00	#DIV/0!	1.2E-01
LMRG_01107	lmo1960	FhuC	1.1E+07	8.1E+06	7.4E-01	1.4E+00	1.9E-02
LMRG_01111	lmo1964		3.1E+06	2.5E+06	8.2E-01	1.2E+00	8.9E-01
LMRG_01112	lmo1965		4.3E+07	3.3E+07	7.8E-01	1.3E+00	9.8E-02
LMRG_01113	lmo1966		6.9E+07	9.5E+07	1.4E+00	7.3E-01	3.1E-02
LMRG_01114	lmo1967		3.6E+08	4.7E+08	1.3E+00	7.7E-01	2.2E-02
LMRG_01121	lmo1974		0.0E+00	6.4E+05	#DIV/0!	0.0E+00	3.7E-01
LMRG_01122	lmo1975	DinB	0.0E+00	6.1E+05	#DIV/0!	0.0E+00	3.7E-01
LMRG_01123	lmo1976		4.5E+07	4.5E+07	9.9E-01	1.0E+00	8.7E-01
LMRG_01124	lmo1977	Rnz	4.2E+07	4.6E+07	1.1E+00	9.1E-01	2.8E-01
LMRG_01127	lmo1979		4.2E+07	4.4E+07	1.0E+00	9.6E-01	7.0E-01
LMRG_01140	lmo1992	AlsD	3.2E+07	2.1E+07	6.5E-01	1.5E+00	1.8E-01
LMRG_01141	lmo1993	Pdp	3.1E+08	3.3E+08	1.1E+00	9.3E-01	2.1E-01
LMRG_01143	lmo1995	DeoC	3.9E+08	2.5E+08	6.4E-01	1.6E+00	3.8E-03
LMRG_01145	lmo1997		0.0E+00	1.2E+06	#DIV/0!	0.0E+00	3.7E-01
LMRG_01146	lmo1998		2.0E+07	5.2E+07	2.6E+00	3.9E-01	8.4E-04
LMRG_01147	lmo1999		0.0E+00	8.8E+05	#DIV/0!	0.0E+00	3.7E-01
LMRG_01150	lmo2002		0.0E+00	1.4E+07	#DIV/0!	0.0E+00	2.3E-05
LMRG_01151	lmo2003		0.0E+00	1.2E+06	#DIV/0!	0.0E+00	3.7E-01
LMRG_01152	lmo2004		0.0E+00	5.9E+06	#DIV/0!	0.0E+00	3.7E-01
LMRG_01153	lmo2005		4.4E+06	1.8E+06	4.0E-01	2.5E+00	4.1E-01
LMRG_01154	lmo2006	AlsS	8.3E+08	7.3E+08	8.8E-01	1.1E+00	1.6E-01
LMRG_01159	lmo2011	YesM	5.7E+06	0.0E+00	0.0E+00	#DIV/0!	3.7E-01
LMRG_01165	lmo2016	CspB	1.5E+09	5.6E+08	3.8E-01	2.7E+00	1.2E-04
LMRG_01166	lmo2017		3.4E+06	0.0E+00	0.0E+00	#DIV/0!	3.7E-01
LMRG_01167	lmo2018	DapF	1.2E+08	7.8E+07	6.4E-01	1.6E+00	5.6E-03
LMRG_01168	lmo2019	IleS	1.7E+09	1.6E+09	9.7E-01	1.0E+00	5.9E-01
LMRG_01169	lmo2020	DivIVA	8.9E+08	8.1E+08	9.1E-01	1.1E+00	1.6E-01
LMRG_01170	lmo2021		2.3E+07	1.6E+07	7.1E-01	1.4E+00	5.4E-02
LMRG_01177	lmo2028		1.1E+06	0.0E+00	0.0E+00	#DIV/0!	3.7E-01
LMRG_01178	lmo2029		5.9E+06	8.5E+06	1.4E+00	6.9E-01	4.3E-01
LMRG_01179	lmo2030	SepF	9.6E+07	1.3E+08	1.4E+00	7.2E-01	2.9E-02
LMRG_01180	lmo2031		5.4E+07	5.6E+07	1.0E+00	9.7E-01	8.2E-01
LMRG_01181	lmo2032	FtsZ	1.4E+09	1.2E+09	8.7E-01	1.1E+00	4.5E-03
LMRG_01182	lmo2033	FtsA	5.8E+08	5.3E+08	9.2E-01	1.1E+00	4.3E-02
LMRG_01183	lmo2034	DivIB	1.6E+07	1.6E+07	9.5E-01	1.0E+00	5.5E-01
LMRG_01184	lmo2035	MurG	2.9E+08	2.7E+08	9.1E-01	1.1E+00	2.1E-01
LMRG_01185	lmo2036	MurD	3.9E+08	3.6E+08	9.2E-01	1.1E+00	4.6E-01
LMRG_01187	lmo2038	MurE	1.8E+08	1.7E+08	9.6E-01	1.0E+00	3.7E-01
LMRG_01188	lmo2039	PbpB2	8.5E+07	7.5E+07	8.9E-01	1.1E+00	2.7E-02
LMRG_01190	lmo2041	RsmH	8.0E+07	4.0E+07	5.0E-01	2.0E+00	9.5E-04
LMRG_01191	lmo2042	MraZ	2.8E+07	1.6E+07	5.8E-01	1.7E+00	3.8E-03
LMRG_01195	lmo2046		2.9E+07	2.2E+07	7.6E-01	1.3E+00	4.6E-01
LMRG_01197	lmo2047	RpmF	1.4E+09	2.2E+09	1.6E+00	6.4E-01	1.3E-02
LMRG_01198	lmo2048		1.2E+08	1.3E+08	1.2E+00	8.6E-01	2.0E-02
LMRG_01200	lmo2050		2.0E+07	0.0E+00	0.0E+00	#DIV/0!	2.9E-05
LMRG_01201	lmo2051		3.2E+07	1.8E+07	5.4E-01	1.8E+00	8.2E-04
LMRG_01202	lmo2052	CoaD	5.7E+06	4.7E+06	8.3E-01	1.2E+00	8.1E-01
LMRG_01203	lmo2053	RsmD	3.5E+07	2.6E+07	7.4E-01	1.4E+00	7.6E-03
LMRG_01204	lmo2054		2.1E+06	1.8E+06	8.8E-01	1.1E+00	9.3E-01
LMRG_01205	lmo2055		1.1E+07	0.0E+00	0.0E+00	#DIV/0!	1.3E-01
LMRG_01206	lmo2056		3.3E+07	3.9E+07	1.2E+00	8.4E-01	3.0E-02

LMRG_01208	lmo2058	CtaA	7.4E+06	0.0E+00	0.0E+00	#DIV/0!	7.6E-05
LMRG_01209	lmo2059		5.9E+06	0.0E+00	0.0E+00	#DIV/0!	3.7E-01
LMRG_01211	lmo2061		3.5E+06	0.0E+00	0.0E+00	#DIV/0!	3.7E-01
LMRG_01214	lmo2064	MscL	1.1E+07	2.6E+06	2.4E-01	4.2E+00	5.2E-02
LMRG_01217	lmo2067	Bsh	2.6E+07	3.5E+07	1.3E+00	7.4E-01	4.0E-02
LMRG_01218	lmo2068	GroL	1.9E+10	1.7E+10	9.2E-01	1.1E+00	5.2E-01
LMRG_01219	lmo2069	GroS	4.4E+09	3.5E+09	8.1E-01	1.2E+00	1.5E-01
LMRG_01223	lmo2072	Rex	2.8E+08	1.4E+08	5.0E-01	2.0E+00	1.2E-03
LMRG_01224	lmo2073		9.9E+07	1.1E+08	1.1E+00	9.0E-01	1.5E-01
LMRG_01225	lmo2074		1.1E+07	1.2E+07	1.1E+00	8.8E-01	4.5E-01
LMRG_01226	lmo2075	TsaD	4.5E+07	3.1E+07	7.0E-01	1.4E+00	1.5E-02
LMRG_01227	lmo2076		7.7E+05	0.0E+00	0.0E+00	#DIV/0!	3.7E-01
LMRG_01228	lmo2077	TsaB	4.9E+07	3.0E+07	6.0E-01	1.7E+00	9.2E-04
LMRG_01229	lmo2078	TsaE	1.8E+07	1.5E+07	8.3E-01	1.2E+00	1.9E-01
LMRG_01230	lmo2079		1.3E+08	5.5E+07	4.4E-01	2.3E+00	4.0E-04
LMRG_01231	lmo2080		2.0E+07	2.1E+07	1.0E+00	9.6E-01	6.7E-01
LMRG_01234	lmo2083		6.8E+07	2.0E+07	3.0E-01	3.3E+00	8.1E-05
LMRG_01239	lmo2088		4.9E+06	7.3E+06	1.5E+00	6.7E-01	3.9E-01
LMRG_01240	lmo2089		4.3E+08	8.8E+07	2.0E-01	4.9E+00	2.6E-05
LMRG_01241	lmo2090	ArgG	1.8E+06	0.0E+00	0.0E+00	#DIV/0!	3.7E-01
LMRG_01242	lmo2091	ArgH	1.6E+06	0.0E+00	0.0E+00	#DIV/0!	3.7E-01
LMRG_01245	lmo2094		0.0E+00	8.0E+06	#DIV/0!	0.0E+00	2.4E-04
LMRG_01252	lmo2101	PdxS	9.5E+08	1.1E+09	1.1E+00	8.8E-01	5.3E-01
LMRG_01253	lmo2102	PdxT	9.1E+07	1.3E+08	1.4E+00	6.9E-01	1.1E-01
LMRG_01255			1.7E+06	7.3E+06	4.3E+00	2.3E-01	3.9E-02
LMRG_01256	lmo2103	Pta	1.2E+09	1.1E+09	8.7E-01	1.1E+00	1.4E-01
LMRG_01258	lmo2105	FeoB	1.5E+07	6.9E+06	4.6E-01	2.2E+00	4.7E-01
LMRG_01260	lmo2106		1.1E+07	1.1E+07	1.0E+00	9.8E-01	8.7E-01
LMRG_01261	lmo2107		1.6E+07	1.3E+07	7.8E-01	1.3E+00	1.4E-01
LMRG_01262	lmo2108	NagA	0.0E+00	1.3E+06	#DIV/0!	0.0E+00	3.7E-01
LMRG_01264	lmo2110	ManA	5.2E+08	4.9E+08	9.4E-01	1.1E+00	4.2E-01
LMRG_01265	lmo2111		4.6E+07	5.7E+07	1.2E+00	8.1E-01	1.1E-02
LMRG_01266	lmo2112		7.9E+06	0.0E+00	0.0E+00	#DIV/0!	1.2E-01
LMRG_01267	lmo2113		1.7E+08	1.9E+07	1.1E-01	9.4E+00	4.4E-05
LMRG_01268	lmo2114	AnrA	1.4E+09	1.9E+09	1.4E+00	7.4E-01	2.0E-01
LMRG_01269	lmo2115	AnrB	5.2E+08	6.6E+08	1.3E+00	7.9E-01	2.3E-01
LMRG_01272	lmo2118	GlmM	7.9E+08	7.1E+08	8.9E-01	1.1E+00	1.6E-01
LMRG_01273	lmo2119	CdaR	1.7E+07	2.3E+07	1.3E+00	7.5E-01	1.8E-01
LMRG_01274	lmo2120	DacA	3.8E+07	4.4E+07	1.2E+00	8.5E-01	1.0E-01
LMRG_01279	lmo2125		0.0E+00	2.9E+06	#DIV/0!	0.0E+00	1.2E-01
LMRG_01282	lmo2128		1.5E+07	1.4E+07	9.2E-01	1.1E+00	6.4E-01
LMRG_01284	lmo2130	KimA	1.2E+07	1.5E+07	1.2E+00	8.1E-01	6.4E-02
LMRG_01286	lmo1681	MetE	1.9E+06	0.0E+00	0.0E+00	#DIV/0!	3.7E-01
LMRG_01290	lmo1677	MenA	1.6E+07	1.7E+07	1.0E+00	9.5E-01	5.7E-01
LMRG_01291	lmo1676	MenF	1.6E+08	1.1E+08	6.8E-01	1.5E+00	1.2E-02
LMRG_01292	lmo1675	MenD	1.3E+08	8.1E+07	6.1E-01	1.6E+00	1.6E-03
LMRG_01294	lmo1673	MenB	8.3E+08	4.1E+08	4.9E-01	2.0E+00	4.4E-04
LMRG_01295	lmo1672	MenE	1.1E+07	5.4E+06	4.8E-01	2.1E+00	1.1E-01
LMRG_01296	lmo1671		3.6E+07	3.9E+07	1.1E+00	9.1E-01	3.7E-01
LMRG_01299	lmo1668		6.1E+07	3.3E+07	5.4E-01	1.9E+00	3.9E-03
LMRG_01300	lmo1667		2.1E+07	1.0E+07	4.9E-01	2.0E+00	5.6E-03
LMRG_01303	lmo1664	MetK	2.1E+08	2.6E+08	1.2E+00	8.0E-01	1.7E-02
LMRG_01304	lmo1663	AnsB	1.5E+09	2.0E+09	1.3E+00	7.6E-01	5.5E-03
LMRG_01305	lmo1662		8.4E+06	1.2E+07	1.4E+00	7.3E-01	1.4E-01
LMRG_01306	lmo1661		4.1E+07	4.6E+07	1.1E+00	8.8E-01	4.1E-01
LMRG_01307	lmo1660	LeuS	1.0E+09	1.1E+09	1.1E+00	9.1E-01	1.4E-01
LMRG_01309	lmo1658	RpsB	8.8E+09	9.0E+09	1.0E+00	9.7E-01	4.0E-01
LMRG_01310	lmo1657	Tsf	1.2E+10	1.2E+10	1.0E+00	9.6E-01	4.0E-01
LMRG_01315	lmo1652		6.5E+05	0.0E+00	0.0E+00	#DIV/0!	3.7E-01
LMRG_01316	lmo1651		5.6E+06	0.0E+00	0.0E+00	#DIV/0!	1.2E-01
LMRG_01318	lmo1649		8.3E+08	8.0E+08	9.6E-01	1.0E+00	4.9E-01
LMRG_01319	lmo1647	PlsC	1.4E+08	1.3E+08	9.2E-01	1.1E+00	3.5E-01
LMRG_01320	lmo1646	SbcD	2.2E+07	1.4E+07	6.5E-01	1.5E+00	7.9E-03
LMRG_01321	lmo1645	SbcC	1.3E+08	1.1E+08	8.5E-01	1.2E+00	1.1E-01
LMRG_01322	lmo1644		2.1E+07	1.6E+07	7.8E-01	1.3E+00	1.2E-02
LMRG_01323	lmo1643		7.2E+07	4.5E+07	6.2E-01	1.6E+00	3.3E-03
LMRG_01324	lmo1642	RsbR	1.8E+07	1.6E+07	9.0E-01	1.1E+00	3.6E-01
LMRG_01325	lmo1641	CitB	2.8E+08	3.6E+08	1.3E+00	7.6E-01	1.6E-03
LMRG_01327	lmo1639	Tag	1.7E+06	4.4E+06	2.7E+00	3.7E-01	3.7E-01
LMRG_01328	lmo1638		6.8E+06	1.2E+07	1.8E+00	5.5E-01	2.0E-01
LMRG_01329	lmo1637		7.4E+07	7.7E+07	1.0E+00	9.6E-01	8.1E-01
LMRG_01330	lmo1636		1.7E+08	1.8E+08	1.1E+00	9.2E-01	4.2E-01
LMRG_01331	lmo1635		0.0E+00	7.1E+05	#DIV/0!	0.0E+00	3.7E-01
LMRG_01332	lmo1634	Lap	4.1E+10	4.7E+10	1.1E+00	8.7E-01	7.0E-03
LMRG_01333	lmo1633	TrpE	1.2E+07	5.8E+06	4.9E-01	2.1E+00	1.9E-03
LMRG_01334	lmo1632	TrpG	2.3E+06	0.0E+00	0.0E+00	#DIV/0!	3.7E-01
LMRG_01335	lmo1631	TrpD	9.2E+06	0.0E+00	0.0E+00	#DIV/0!	1.1E-03
LMRG_01336	lmo1630	TrpC	1.9E+07	9.1E+06	4.8E-01	2.1E+00	1.4E-05
LMRG_01337	lmo1629	TrpF	3.9E+07	2.9E+07	7.3E-01	1.4E+00	1.8E-03
LMRG_01338	lmo1628	TrpB	5.4E+07	3.2E+07	6.0E-01	1.7E+00	4.9E-04
LMRG_01339	lmo1627	TrpA	7.2E+07	4.1E+07	5.7E-01	1.8E+00	6.1E-03
LMRG_01340	lmo1626		0.0E+00	1.7E+07	#DIV/0!	0.0E+00	3.7E-01
LMRG_01341	lmo1625		3.9E+06	2.0E+06	5.2E-01	1.9E+00	4.1E-01
LMRG_01344	lmo1622	NnrD	1.9E+08	0.0E+00	0.0E+00	#DIV/0!	3.7E-01
LMRG_01345	lmo1621		1.3E+08	1.2E+08	9.5E-01	1.1E+00	5.5E-01
LMRG_01346	lmo1620	PepV	1.6E+09	1.2E+09	7.6E-01	1.3E+00	3.0E-03

LMRG_01347	lmo1619	Dat	3.6E+08	5.7E+08	1.6E+00	6.2E-01	1.9E-02
LMRG_01350	lmo1616		2.4E+07	2.0E+07	8.6E-01	1.2E+00	4.4E-01
LMRG_01351	lmo1615	TrmB	8.5E+07	8.1E+07	9.5E-01	1.1E+00	5.0E-01
LMRG_01353	lmo1613		3.1E+06	1.2E+06	3.7E-01	2.7E+00	3.7E-01
LMRG_01354	lmo1612		4.5E+07	5.4E+07	1.2E+00	8.4E-01	1.9E-02
LMRG_01355	lmo1611		6.1E+08	4.4E+08	7.2E-01	1.4E+00	7.1E-03
LMRG_01357	lmo1609	YtpP	4.5E+07	2.2E+07	4.9E-01	2.0E+00	5.2E-03
LMRG_01358	lmo1608		2.4E+08	8.6E+07	3.6E-01	2.8E+00	1.3E-02
LMRG_01359	lmo1607		5.5E+08	4.8E+08	8.7E-01	1.2E+00	1.1E-01
LMRG_01360	lmo1606	FtsK/SpoIIIE	1.5E+08	2.0E+08	1.3E+00	7.8E-01	2.0E-02
LMRG_01361	lmo1605	MurC	4.8E+08	6.1E+08	1.3E+00	7.9E-01	7.4E-02
LMRG_01363	lmo1604	AhpA	4.6E+08	4.2E+08	9.1E-01	1.1E+00	2.1E-01
LMRG_01364	lmo1603		1.0E+09	1.1E+09	1.1E+00	9.1E-01	6.4E-02
LMRG_01365	lmo1602		5.1E+08	2.5E+08	4.8E-01	2.1E+00	3.5E-04
LMRG_01366	lmo1601		8.5E+08	4.0E+08	4.7E-01	2.1E+00	1.0E-03
LMRG_01367	lmo1600	AroA	4.0E+08	5.7E+08	1.4E+00	7.0E-01	2.3E-03
LMRG_01368	lmo1599	CcpA	1.0E+09	9.7E+08	9.4E-01	1.1E+00	3.5E-01
LMRG_01369	lmo1598	TyrS	6.4E+08	7.6E+08	1.2E+00	8.4E-01	1.7E-03
LMRG_01371	lmo1596	RpsD	6.3E+09	7.6E+09	1.2E+00	8.3E-01	1.7E-02
LMRG_01372	lmo1595		3.6E+07	1.4E+07	3.9E-01	2.5E+00	5.3E-02
LMRG_01373	lmo1594	EzrA	3.9E+08	3.8E+08	9.8E-01	1.0E+00	6.1E-01
LMRG_01374	lmo1593	NifS/IcsS	8.1E+08	6.4E+08	7.9E-01	1.3E+00	2.0E-01
LMRG_01375	lmo1592	ThiI	2.3E+08	2.8E+08	1.2E+00	8.2E-01	4.2E-02
LMRG_01381	lmo1586	NadK	4.6E+08	4.2E+08	9.2E-01	1.1E+00	3.0E-01
LMRG_01382	lmo1585	SppA	3.7E+07	1.6E+07	4.3E-01	2.3E+00	4.0E-03
LMRG_01384	lmo1583	Tpx	7.2E+07	0.0E+00	0.0E+00	#DIV/0!	1.2E-07
LMRG_01385	lmo1582		9.1E+07	1.0E+08	1.1E+00	9.0E-01	1.7E-01
LMRG_01386	lmo1581	AckA	2.0E+09	2.3E+09	1.2E+00	8.6E-01	2.9E-03
LMRG_01387	lmo1580		1.2E+09	1.3E+09	1.0E+00	9.8E-01	7.8E-01
LMRG_01388	lmo1579	Ald	2.4E+08	6.8E+07	2.8E-01	3.6E+00	1.8E-04
LMRG_01389	lmo1578		2.5E+08	8.2E+07	3.3E-01	3.0E+00	3.5E-05
LMRG_01390	lmo1577		1.4E+08	1.1E+08	7.6E-01	1.3E+00	1.1E-01
LMRG_01391	lmo1576	YtoI	3.7E+07	2.7E+07	7.3E-01	1.4E+00	2.8E-02
LMRG_01392	lmo1575		9.4E+07	8.5E+07	9.0E-01	1.1E+00	2.0E-01
LMRG_01394	lmo1573	AccD	2.6E+08	1.8E+08	7.1E-01	1.4E+00	5.2E-04
LMRG_01395	lmo1572	AccA	2.7E+08	1.8E+08	6.5E-01	1.5E+00	4.9E-03
LMRG_01396	lmo1571	PfkA	3.3E+09	3.7E+09	1.1E+00	9.1E-01	2.6E-01
LMRG_01397	lmo1570	PykA	1.1E+10	1.0E+10	9.2E-01	1.1E+00	9.1E-03
LMRG_01398	lmo1569	FxsA	5.0E+05	0.0E+00	0.0E+00	#DIV/0!	3.7E-01
LMRG_01400	lmo1567	CitZ	2.2E+07	0.0E+00	0.0E+00	#DIV/0!	8.4E-03
LMRG_01401	lmo1566	CitC	1.8E+08	9.4E+07	5.4E-01	1.9E+00	3.5E-04
LMRG_01402	lmo1565	PolA	2.1E+08	1.3E+08	6.4E-01	1.6E+00	1.0E-03
LMRG_01403	lmo1564	MutM	3.1E+07	9.5E+06	3.0E-01	3.3E+00	2.3E-03
LMRG_01405	lmo1562	NrdR	8.1E+07	9.6E+07	1.2E+00	8.4E-01	1.2E-02
LMRG_01406	lmo1561	DnaB	2.2E+07	2.2E+07	9.6E-01	1.0E+00	6.0E-01
LMRG_01407	lmo1560	DnaI	1.9E+07	1.6E+07	8.3E-01	1.2E+00	3.3E-02
LMRG_01408	lmo1559	ThrS	7.9E+08	8.4E+08	1.1E+00	9.3E-01	2.4E-01
LMRG_01409	lmo1558	EngB	1.0E+08	9.9E+07	9.5E-01	1.0E+00	6.4E-01
LMRG_01412	lmo1556	HemC	5.5E+07	3.5E+07	6.3E-01	1.6E+00	8.3E-04
LMRG_01414	lmo1554	HemB	2.7E+08	1.8E+08	6.8E-01	1.5E+00	3.6E-03
LMRG_01415	lmo1553	HemL	1.6E+08	1.1E+08	6.7E-01	1.5E+00	1.7E-03
LMRG_01416	lmo1552	ValS	1.3E+09	1.3E+09	1.0E+00	9.7E-01	5.6E-01
LMRG_01417	lmo1551	FolC	1.2E+08	1.4E+08	1.2E+00	8.6E-01	1.2E-02
LMRG_01422	lmo1548	MreBH	1.9E+09	2.6E+09	1.4E+00	7.2E-01	1.1E-03
LMRG_01423	lmo1547	MreC	1.8E+08	2.5E+08	1.4E+00	7.1E-01	3.3E-03
LMRG_01425	lmo1545	MinC	8.0E+07	8.0E+07	1.0E+00	1.0E+00	1.0E+00
LMRG_01426	lmo1544	MinD	2.6E+08	3.1E+08	1.2E+00	8.6E-01	4.4E-02
LMRG_01427	lmo1543		2.9E+07	3.8E+07	1.3E+00	7.7E-01	5.5E-02
LMRG_01428	lmo1542	RplU	4.1E+09	4.1E+09	9.8E-01	1.0E+00	8.8E-01
LMRG_01429	lmo1541		1.1E+07	1.8E+07	1.6E+00	6.3E-01	3.3E-01
LMRG_01430	lmo1540	RpmA	2.2E+09	1.9E+09	8.6E-01	1.2E+00	2.0E-03
LMRG_01432	lmo1538	GlpK	1.3E+08	1.3E+08	1.0E+00	1.0E+00	1.0E+00
LMRG_01433	lmo1537	Obg	4.5E+08	5.7E+08	1.3E+00	8.0E-01	2.7E-02
LMRG_01434	lmo1536	PheA	2.8E+07	2.6E+07	9.5E-01	1.1E+00	5.2E-01
LMRG_01435	lmo1535		7.7E+06	5.5E+06	7.1E-01	1.4E+00	1.5E-01
LMRG_01436	lmo1534	Ldh	4.6E+08	4.5E+08	9.9E-01	1.0E+00	8.6E-01
LMRG_01437	lmo1533	RuvA	7.4E+07	7.6E+07	1.0E+00	9.7E-01	7.4E-01
LMRG_01438	lmo1532	RuvB	5.5E+07	4.9E+07	8.9E-01	1.1E+00	3.3E-01
LMRG_01439	lmo1531	QueA	2.1E+08	2.6E+08	1.2E+00	8.3E-01	6.9E-02
LMRG_01440	lmo1530	Tgt	2.2E+08	3.1E+08	1.4E+00	7.2E-01	1.2E-02
LMRG_01441	lmo1529	YajC	4.0E+08	5.0E+08	1.3E+00	7.9E-01	1.6E-01
LMRG_01442	lmo1528		2.1E+07	1.4E+07	6.7E-01	1.5E+00	2.8E-01
LMRG_01443	lmo1527	SecD	5.8E+08	3.7E+08	6.4E-01	1.6E+00	8.0E-04
LMRG_01444	lmo1526		0.0E+00	2.4E+06	#DIV/0!	0.0E+00	3.7E-01
LMRG_01445	lmo1525	RecJ	1.1E+08	1.4E+08	1.3E+00	7.5E-01	2.4E-02
LMRG_01446	lmo1524	Apt	2.7E+08	3.1E+08	1.1E+00	8.8E-01	8.0E-02
LMRG_01447	lmo1523	RelA	1.1E+08	1.3E+08	1.2E+00	8.6E-01	9.8E-02
LMRG_01448	lmo1522	Dtd	8.1E+06	0.0E+00	0.0E+00	#DIV/0!	1.1E-03
LMRG_01449	lmo1521		4.4E+07	3.9E+07	8.9E-01	1.1E+00	3.8E-01
LMRG_01450	lmo1520	HisS	6.8E+08	7.0E+08	1.0E+00	9.8E-01	5.1E-01
LMRG_01451	lmo1519	AspS	1.1E+09	1.3E+09	1.1E+00	8.9E-01	5.4E-02
LMRG_01452	lmo1518		2.7E+06	2.3E+07	8.4E+00	1.2E-01	3.5E-03
LMRG_01455	lmo1515	CymR	1.6E+08	1.1E+08	6.7E-01	1.5E+00	1.0E-02
LMRG_01456	lmo1514		3.0E+07	3.0E+07	9.8E-01	1.0E+00	6.4E-01
LMRG_01457	lmo1513		5.5E+07	3.7E+07	6.7E-01	1.5E+00	3.8E-01
LMRG_01458	lmo1512	MnmA	1.9E+08	2.0E+08	1.1E+00	9.5E-01	3.0E-01

LMRG_01459	lmo1511		5.6E+07	4.7E+07	8.3E-01	1.2E+00	2.0E-01
LMRG_01460	lmo1510		3.3E+08	3.4E+08	1.0E+00	9.9E-01	9.3E-01
LMRG_01461	lmo1509	RecD2	1.2E+07	1.0E+07	8.7E-01	1.2E+00	2.2E-01
LMRG_01462	lmo1508		0.0E+00	3.8E+05	#DIV/0!	0.0E+00	3.7E-01
LMRG_01463	lmo1507		6.7E+06	5.7E+06	8.5E-01	1.2E+00	4.7E-01
LMRG_01464	lmo1506		0.0E+00	1.4E+06	#DIV/0!	0.0E+00	3.7E-01
LMRG_01465	lmo1505		0.0E+00	1.5E+06	#DIV/0!	0.0E+00	3.7E-01
LMRG_01466	lmo1504	AlaS	1.7E+09	2.0E+09	1.1E+00	8.7E-01	2.7E-02
LMRG_01467	lmo1503	ReoM	2.2E+08	1.8E+08	8.1E-01	1.2E+00	4.7E-02
LMRG_01468	lmo1502	RuvX	4.3E+07	5.0E+07	1.2E+00	8.6E-01	2.6E-01
LMRG_01469	lmo1501		3.8E+07	3.9E+07	1.0E+00	9.9E-01	9.0E-01
LMRG_01471	lmo1499	MltG	1.5E+08	1.6E+08	1.1E+00	9.1E-01	9.4E-02
LMRG_01472	lmo1498	TrmR	5.7E+07	7.8E+07	1.4E+00	7.2E-01	2.6E-02
LMRG_01473	lmo1497	Udk	2.1E+07	3.1E+07	1.5E+00	6.9E-01	2.7E-01
LMRG_01474	lmo1496	GreA	9.9E+08	7.6E+08	7.7E-01	1.3E+00	3.2E-03
LMRG_01475	lmo1495		5.5E+07	4.9E+07	8.9E-01	1.1E+00	4.5E-01
LMRG_01476	lmo1494	MtnN	2.7E+08	2.0E+08	7.4E-01	1.4E+00	1.4E-02
LMRG_01479	lmo2363	GadD2	5.0E+09	3.8E+09	7.7E-01	1.3E+00	6.5E-02
LMRG_01480	lmo2362	GadT2	6.7E+07	5.8E+07	8.7E-01	1.2E+00	1.8E-01
LMRG_01481	lmo2361		1.4E+08	1.6E+08	1.2E+00	8.4E-01	5.4E-01
LMRG_01482	lmo2360		1.5E+09	1.5E+09	1.0E+00	9.6E-01	8.9E-01
LMRG_01483	lmo2359		1.3E+07	1.8E+07	1.3E+00	7.6E-01	4.3E-01
LMRG_01484	lmo2358		1.9E+08	1.5E+08	8.0E-01	1.2E+00	9.9E-02
LMRG_01488	lmo2355		0.0E+00	1.6E+06	#DIV/0!	0.0E+00	3.7E-01
LMRG_01489	lmo2354		6.6E+07	7.2E+07	1.1E+00	9.1E-01	5.1E-01
LMRG_01490	lmo2353		5.7E+06	9.8E+06	1.7E+00	5.8E-01	2.4E-01
LMRG_01501	lmo2342		6.9E+06	2.0E+06	2.9E-01	3.5E+00	6.9E-02
LMRG_01502	lmo2341		8.9E+06	2.5E+06	2.8E-01	3.6E+00	6.7E-02
LMRG_01503	lmo2340	PsuG	3.4E+08	3.2E+08	9.4E-01	1.1E+00	4.3E-01
LMRG_01505	lmo2338	PepC	5.6E+07	2.7E+07	4.9E-01	2.1E+00	3.8E-04
LMRG_01506	lmo2337		3.5E+07	4.1E+07	1.2E+00	8.4E-01	3.0E-01
LMRG_01507	lmo2336	FruB	1.7E+08	2.8E+08	1.6E+00	6.1E-01	3.9E-02
LMRG_01508	lmo2335	FruA	8.1E+08	1.1E+09	1.3E+00	7.6E-01	8.8E-02
LMRG_01509	lmo2334		2.0E+07	1.0E+07	4.9E-01	2.0E+00	2.5E-03
LMRG_01511	lmo2332	Int	0.0E+00	0.0E+00	#DIV/0!	#DIV/0!	#DIV/0!
LMRG_01512			1.0E+07	8.0E+06	8.0E-01	1.2E+00	9.7E-02
LMRG_01513			2.3E+07	1.6E+07	6.8E-01	1.5E+00	3.8E-02
LMRG_01514	lmo2329		5.3E+07	5.5E+07	1.0E+00	9.7E-01	9.0E-01
LMRG_01530			7.3E+05	0.0E+00	0.0E+00	#DIV/0!	3.7E-01
LMRG_01545	lmo2287		0.0E+00	1.4E+07	#DIV/0!	0.0E+00	1.5E-01
LMRG_01555			4.6E+06	0.0E+00	0.0E+00	#DIV/0!	3.7E-01
LMRG_01562	lmo2268	AddB	1.9E+07	2.5E+07	1.3E+00	7.5E-01	2.1E-02
LMRG_01565	lmo2266		1.6E+07	2.1E+07	1.3E+00	7.9E-01	7.6E-01
LMRG_01568	lmo2263		4.7E+07	4.6E+07	1.0E+00	1.0E+00	9.7E-01
LMRG_01569	lmo2262	YjfP	1.1E+07	1.2E+07	1.1E+00	9.4E-01	6.4E-01
LMRG_01570	lmo2261		0.0E+00	1.3E+06	#DIV/0!	0.0E+00	3.7E-01
LMRG_01571	lmo2260		0.0E+00	1.2E+06	#DIV/0!	0.0E+00	3.7E-01
LMRG_01572	lmo2259		5.1E+06	0.0E+00	0.0E+00	#DIV/0!	3.7E-01
LMRG_01573	lmo2258		3.3E+07	6.0E+07	1.8E+00	5.5E-01	6.1E-02
LMRG_01575	lmo2256		1.0E+08	2.5E+07	2.4E-01	4.1E+00	5.8E-03
LMRG_01577	lmo2254		8.9E+06	2.3E+07	2.6E+00	3.8E-01	3.5E-02
LMRG_01578	lmo2253		1.7E+07	3.6E+06	2.1E-01	4.8E+00	2.2E-02
LMRG_01579	lmo2252		1.5E+08	2.2E+08	1.4E+00	6.9E-01	1.6E-01
LMRG_01580	lmo2251	GlnQ	9.8E+07	1.3E+08	1.3E+00	7.4E-01	2.3E-01
LMRG_01581	lmo2250	ArpJ	6.7E+07	1.0E+08	1.6E+00	6.4E-01	9.0E-02
LMRG_01582	lmo2249		1.7E+07	7.9E+07	4.6E+00	2.2E-01	2.2E-01
LMRG_01583	lmo2248		7.4E+08	6.9E+08	9.3E-01	1.1E+00	3.9E-01
LMRG_01585	lmo2247		6.4E+07	2.2E+07	3.5E-01	2.8E+00	1.1E-04
LMRG_01588	lmo2244		7.8E+07	1.3E+08	1.7E+00	6.0E-01	9.6E-04
LMRG_01592	lmo2240		2.9E+06	2.5E+06	8.5E-01	1.2E+00	9.1E-01
LMRG_01593	lmo2239		1.3E+06	3.1E+06	2.3E+00	4.3E-01	4.4E-01
LMRG_01599	lmo2233		0.0E+00	3.2E+05	#DIV/0!	0.0E+00	3.7E-01
LMRG_01600	lmo2232		6.9E+07	7.7E+07	1.1E+00	8.9E-01	4.7E-02
LMRG_01603	lmo2229	PbpA2	1.4E+08	1.6E+08	1.1E+00	8.7E-01	1.4E-01
LMRG_01607	lmo2225	FumC	5.2E+07	3.8E+07	7.3E-01	1.4E+00	1.3E-02
LMRG_01608	lmo2224		4.2E+06	1.7E+07	4.2E+00	2.4E-01	4.2E-02
LMRG_01609	lmo2223		1.6E+09	8.4E+08	5.3E-01	1.9E+00	7.0E-04
LMRG_01611	lmo2221		3.1E+06	1.5E+07	4.9E+00	2.1E-01	2.0E-02
LMRG_01612	lmo2220	YhaM	1.2E+08	1.3E+08	1.1E+00	9.4E-01	4.8E-01
LMRG_01613	lmo2219	PrsA2	1.9E+09	2.9E+09	1.6E+00	6.4E-01	1.6E-02
LMRG_01614	lmo2218		0.0E+00	5.4E+05	#DIV/0!	0.0E+00	3.7E-01
LMRG_01615	lmo2217		1.1E+08	6.9E+07	6.2E-01	1.6E+00	4.5E-02
LMRG_01616	lmo2216		5.9E+07	2.8E+07	4.7E-01	2.1E+00	4.6E-02
LMRG_01617	lmo2215		2.1E+07	6.8E+06	3.3E-01	3.1E+00	1.5E-02
LMRG_01618	lmo2214		4.4E+06	1.5E+06	3.4E-01	3.0E+00	3.4E-01
LMRG_01619	lmo2213	Isd-LmHde	9.6E+07	1.3E+08	1.4E+00	7.1E-01	2.4E-02
LMRG_01620	lmo2212	HemE	7.6E+07	0.0E+00	0.0E+00	#DIV/0!	1.2E-04
LMRG_01621	lmo2211	HemH	2.9E+08	0.0E+00	0.0E+00	#DIV/0!	1.6E-07
LMRG_01623	lmo2209		1.8E+07	2.5E+07	1.4E+00	7.0E-01	5.6E-02
LMRG_01624	lmo2208		8.3E+07	1.0E+08	1.2E+00	8.1E-01	9.3E-03
LMRG_01625	lmo2207		3.2E+06	0.0E+00	0.0E+00	#DIV/0!	3.7E-01
LMRG_01626	lmo2206	ClpB	1.3E+09	1.2E+09	8.6E-01	1.2E+00	1.5E-01
LMRG_01627	lmo2205	GpmA	2.1E+08	2.2E+08	1.0E+00	9.7E-01	7.6E-01
LMRG_01628	lmo2204		0.0E+00	2.0E+06	#DIV/0!	0.0E+00	3.7E-01
LMRG_01630	lmo2202	FabH	9.9E+08	8.2E+08	8.3E-01	1.2E+00	1.5E-01
LMRG_01631	lmo2201	FabF	2.1E+09	1.4E+09	6.8E-01	1.5E+00	3.0E-03

LMRG_01633	lmo2199	OhrA	6.0E+07	4.3E+07	7.2E-01	1.4E+00	4.7E-02
LMRG_01634	lmo2198	TrpS	3.0E+08	3.0E+08	1.0E+00	9.9E-01	8.8E-01
LMRG_01636	lmo2196	OppA	8.6E+09	5.1E+09	5.9E-01	1.7E+00	1.5E-04
LMRG_01637	lmo2195	OppB	1.2E+08	1.2E+08	1.0E+00	9.7E-01	7.4E-01
LMRG_01638	lmo2194	OppC	3.3E+08	3.1E+08	9.5E-01	1.1E+00	3.9E-01
LMRG_01639	lmo2193	OppD	7.7E+08	7.9E+08	1.0E+00	9.8E-01	7.5E-01
LMRG_01640	lmo2192	OppF	1.2E+09	1.2E+09	9.8E-01	1.0E+00	8.0E-01
LMRG_01641	lmo2191	SpxA1	6.4E+07	0.0E+00	0.0E+00	#DIV/0!	6.7E-06
LMRG_01642	lmo2190	MecA	3.8E+07	7.4E+08	1.9E+01	5.2E-02	1.8E-04
LMRG_01643	lmo2189		0.0E+00	1.6E+06	#DIV/0!	0.0E+00	3.7E-01
LMRG_01644	lmo2188	PepF	2.9E+08	9.2E+07	3.2E-01	3.1E+00	2.1E-04
LMRG_01645	lmo2187		0.0E+00	6.5E+05	#DIV/0!	0.0E+00	3.7E-01
LMRG_01657	lmo2175	FabG	7.4E+05	0.0E+00	0.0E+00	#DIV/0!	3.7E-01
LMRG_01659	lmo2173		2.4E+06	6.1E+06	2.5E+00	4.0E-01	4.2E-01
LMRG_01662	lmo2170	FabK2	6.0E+06	1.1E+07	1.9E+00	5.3E-01	2.1E-01
LMRG_01664	lmo2168		7.2E+07	2.7E+07	3.8E-01	2.6E+00	2.2E-04
LMRG_01665	lmo2167		1.5E+08	6.8E+07	4.6E-01	2.2E+00	1.8E-02
LMRG_01666	lmo2166		7.3E+07	3.4E+07	4.7E-01	2.1E+00	1.9E-04
LMRG_01669	lmo2163		0.0E+00	1.3E+06	#DIV/0!	0.0E+00	3.7E-01
LMRG_01673	lmo2159		2.1E+07	0.0E+00	0.0E+00	#DIV/0!	1.2E-01
LMRG_01674	lmo2158		0.0E+00	1.3E+06	#DIV/0!	0.0E+00	3.7E-01
LMRG_01675	lmo2157	SepA	5.1E+07	6.2E+07	1.2E+00	8.2E-01	1.2E-01
LMRG_01677	lmo2155	NrdA	6.7E+08	5.0E+08	7.4E-01	1.4E+00	1.4E-01
LMRG_01678	lmo2154	NrdF	3.4E+08	2.5E+08	7.4E-01	1.4E+00	8.5E-02
LMRG_01679	lmo2153		3.5E+07	2.9E+07	8.2E-01	1.2E+00	1.3E-01
LMRG_01680	lmo2152		5.8E+07	2.8E+07	4.9E-01	2.0E+00	3.5E-02
LMRG_01683	lmo2149		4.2E+07	7.8E+07	1.8E+00	5.4E-01	1.8E-02
LMRG_01688	lmo2144		2.2E+06	1.7E+06	7.9E-01	1.3E+00	8.8E-01
LMRG_01690	lmo2557		6.8E+06	0.0E+00	0.0E+00	#DIV/0!	2.0E-04
LMRG_01691	lmo2556	FbaA	1.2E+10	1.7E+10	1.4E+00	7.2E-01	9.0E-04
LMRG_01692	lmo2555	LafA	4.3E+07	5.3E+07	1.2E+00	8.2E-01	1.4E-01
LMRG_01693	lmo2554	LafB	4.1E+07	4.7E+07	1.1E+00	8.7E-01	1.5E-01
LMRG_01695	lmo2552	MurA	2.0E+08	2.2E+08	1.1E+00	9.1E-01	4.1E-02
LMRG_01696	lmo2551	Rho	1.1E+08	1.2E+08	1.1E+00	9.3E-01	1.7E-01
LMRG_01697	lmo2550		4.9E+07	5.1E+07	1.0E+00	9.7E-01	7.0E-01
LMRG_01699	lmo2548	RpmE2	3.6E+09	3.6E+09	1.0E+00	9.9E-01	9.5E-01
LMRG_01700	lmo2547	Hom	1.9E+08	2.1E+08	1.1E+00	9.4E-01	1.7E-01
LMRG_01701	lmo2546	ThrC	3.4E+08	3.9E+08	1.1E+00	8.8E-01	5.3E-02
LMRG_01702	lmo2545	ThrB	8.4E+07	8.2E+07	9.8E-01	1.0E+00	6.1E-01
LMRG_01703	lmo2544	Tdk	1.7E+08	1.5E+08	8.7E-01	1.1E+00	1.8E-01
LMRG_01704	lmo2543	PrfA	5.6E+08	6.5E+08	1.2E+00	8.7E-01	3.5E-02
LMRG_01705	lmo2542	PrmC	2.6E+07	3.7E+07	1.4E+00	7.1E-01	5.8E-02
LMRG_01706	lmo2541		4.5E+07	4.5E+07	1.0E+00	1.0E+00	9.5E-01
LMRG_01707	lmo2540		9.6E+06	7.8E+06	8.1E-01	1.2E+00	9.2E-02
LMRG_01708	lmo2539	GlyA	2.3E+08	1.5E+08	6.4E-01	1.6E+00	5.7E-04
LMRG_01709	lmo2538	Upp	1.9E+09	2.3E+09	1.2E+00	8.2E-01	7.6E-03
LMRG_01710	lmo2537	MnaA	1.9E+08	2.2E+08	1.2E+00	8.4E-01	7.8E-03
LMRG_01713	lmo2535	AtpB	0.0E+00	2.1E+07	#DIV/0!	0.0E+00	9.6E-04
LMRG_01715	lmo2533	AtpF	5.4E+08	1.1E+09	2.1E+00	4.8E-01	1.3E-03
LMRG_01716	lmo2532	AtpH	3.6E+08	5.0E+08	1.4E+00	7.2E-01	1.8E-03
LMRG_01717	lmo2531	AtpA	3.8E+09	4.9E+09	1.3E+00	7.6E-01	3.4E-04
LMRG_01718	lmo2530	AtpG	3.8E+08	4.9E+08	1.3E+00	7.7E-01	9.4E-03
LMRG_01719	lmo2529	AtpD2	4.8E+09	6.3E+09	1.3E+00	7.6E-01	3.0E-03
LMRG_01720	lmo2528	AtpC	2.9E+08	3.5E+08	1.2E+00	8.1E-01	7.4E-02
LMRG_01722	lmo2526	MurA	6.3E+08	1.6E+09	2.5E+00	4.0E-01	4.7E-05
LMRG_01723	lmo2525	Mbl	5.2E+08	5.5E+08	1.0E+00	9.5E-01	3.0E-01
LMRG_01727	lmo2521	TagA	3.8E+06	4.1E+06	1.1E+00	9.3E-01	9.6E-01
LMRG_01728	lmo2520	MenC	0.0E+00	0.0E+00	#DIV/0!	#DIV/0!	#DIV/0!
LMRG_01730	lmo2518	LytR	6.3E+08	6.7E+08	1.1E+00	9.4E-01	1.1E-01
LMRG_01731	lmo2517		1.1E+07	1.1E+07	9.8E-01	1.0E+00	9.7E-01
LMRG_01732	lmo2516		3.9E+07	4.3E+07	1.1E+00	9.0E-01	3.3E-01
LMRG_01733	lmo2515	DegU	1.6E+08	1.3E+08	8.0E-01	1.2E+00	7.5E-02
LMRG_01734	lmo2514		3.1E+07	2.5E+07	8.2E-01	1.2E+00	2.1E-01
LMRG_01737	lmo2511	Hpf	9.3E+08	3.2E+08	3.5E-01	2.9E+00	1.1E-03
LMRG_01738	lmo2510	SecA	1.0E+09	9.2E+08	8.8E-01	1.1E+00	8.8E-02
LMRG_01739	lmo2509	PrfB	1.6E+08	1.5E+08	9.1E-01	1.1E+00	3.9E-01
LMRG_01740	lmo2508		0.0E+00	9.7E+05	#DIV/0!	0.0E+00	3.7E-01
LMRG_01741	lmo2507	FtsE	7.0E+08	9.0E+08	1.3E+00	7.8E-01	7.1E-02
LMRG_01742	lmo2506	FtsX	1.9E+08	2.6E+08	1.3E+00	7.4E-01	1.2E-02
LMRG_01743	lmo2505	Spl	1.9E+08	2.0E+08	1.0E+00	9.7E-01	7.5E-01
LMRG_01744	lmo2504		7.3E+06	0.0E+00	0.0E+00	#DIV/0!	1.7E-03
LMRG_01745	lmo2503	Cls	3.0E+07	1.8E+07	6.1E-01	1.7E+00	1.5E-03
LMRG_01746	lmo2502	MinJ	9.4E+06	7.0E+06	7.5E-01	1.3E+00	5.5E-01
LMRG_01747	lmo2501	PhoP	1.8E+07	1.3E+07	7.3E-01	1.4E+00	5.0E-02
LMRG_01748	lmo2500	PhoR	8.9E+06	6.4E+06	7.1E-01	1.4E+00	1.1E-03
LMRG_01755	lmo2493	CzrA	3.2E+07	1.5E+07	4.8E-01	2.1E+00	9.1E-03
LMRG_01757	lmo2491	PdeE	4.3E+07	2.8E+07	6.5E-01	1.5E+00	7.3E-04
LMRG_01759	lmo2489	UvrB	6.4E+07	7.5E+07	1.2E+00	8.5E-01	9.5E-02
LMRG_01760	lmo2488	UvrA	3.0E+08	3.0E+08	1.0E+00	1.0E+00	9.6E-01
LMRG_01761	lmo2487		4.3E+08	8.0E+08	1.8E+00	5.4E-01	2.3E-02
LMRG_01762	lmo2486		5.0E+07	7.7E+07	1.5E+00	6.6E-01	7.0E-02
LMRG_01765	lmo2483	HprK	1.1E+08	8.8E+07	7.6E-01	1.3E+00	6.1E-04
LMRG_01766	lmo2482	Lgt	0.0E+00	2.9E+06	#DIV/0!	0.0E+00	3.7E-01
LMRG_01767	lmo2481	PpaX	1.1E+08	9.9E+07	8.6E-01	1.2E+00	2.8E-01
LMRG_01769	lmo2479		7.3E+06	1.4E+07	2.0E+00	5.1E-01	1.6E-01
LMRG_01770	lmo2478	TrxB	2.2E+08	6.9E+07	3.1E-01	3.2E+00	6.6E-05

LMRG_01771	lmo2477	GalE	4.7E+07	2.0E+07	4.1E-01	2.4E+00	4.2E-03
LMRG_01772	lmo2476		7.9E+05	0.0E+00	0.0E+00	#DIV/0!	3.7E-01
LMRG_01773	lmo2475		5.3E+08	3.0E+08	5.7E-01	1.7E+00	4.4E-03
LMRG_01774	lmo2474	RapZ	8.9E+07	6.6E+07	7.3E-01	1.4E+00	1.9E-02
LMRG_01775	lmo2473	YvcK	5.0E+07	3.7E+07	7.5E-01	1.3E+00	1.6E-02
LMRG_01776	lmo2472	WhiA	5.0E+06	1.0E+07	2.1E+00	4.8E-01	1.2E-01
LMRG_01777	lmo2471	NamA	2.1E+08	1.3E+08	5.9E-01	1.7E+00	1.6E-05
LMRG_01779	lmo2469		0.0E+00	2.1E+06	#DIV/0!	0.0E+00	3.7E-01
LMRG_01780	lmo2468	ClpP1	9.9E+08	5.9E+08	5.9E-01	1.7E+00	6.6E-04
LMRG_01783	lmo2465		8.7E+06	8.4E+06	9.8E-01	1.0E+00	9.7E-01
LMRG_01784	lmo2464		5.0E+06	7.7E+06	1.5E+00	6.5E-01	3.6E-01
LMRG_01786	lmo2462		2.3E+08	3.8E+08	1.7E+00	6.0E-01	7.8E-05
LMRG_01787	lmo2461	SigL	7.6E+07	6.6E+07	8.7E-01	1.2E+00	4.8E-02
LMRG_01788	lmo2460		2.3E+08	2.3E+08	9.8E-01	1.0E+00	6.9E-01
LMRG_01789	lmo2459	Gap	4.7E+10	4.4E+10	9.4E-01	1.1E+00	1.0E-01
LMRG_01790	lmo2458	Pgk	1.3E+10	1.2E+10	9.5E-01	1.0E+00	2.5E-01
LMRG_01791	lmo2457	TpiA	9.6E+09	9.0E+09	9.4E-01	1.1E+00	9.1E-02
LMRG_01792	lmo2456	GpmI	8.6E+09	1.0E+10	1.2E+00	8.5E-01	8.3E-02
LMRG_01793	lmo2455	Eno	4.0E+10	3.8E+10	9.4E-01	1.1E+00	3.5E-01
LMRG_01796	lmo2452		8.5E+07	1.4E+08	1.6E+00	6.1E-01	7.6E-03
LMRG_01797	lmo2451	SecG	3.3E+07	3.8E+07	1.2E+00	8.7E-01	7.8E-01
LMRG_01798	lmo2450		8.1E+07	1.1E+08	1.3E+00	7.6E-01	1.9E-04
LMRG_01799	lmo2449	Rnr	1.7E+08	2.5E+08	1.4E+00	7.0E-01	3.7E-03
LMRG_01800	lmo2448	SmpB	2.0E+07	1.8E+07	8.9E-01	1.1E+00	6.3E-01
LMRG_01809	lmo2439		3.5E+07	6.4E+07	1.8E+00	5.5E-01	1.0E-01
LMRG_01810	lmo2438		1.7E+07	1.4E+07	8.5E-01	1.2E+00	2.4E-01
LMRG_01814	lmo2434	GadD3	2.5E+07	3.2E+07	1.3E+00	7.9E-01	3.8E-01
LMRG_01815	lmo2433		6.2E+07	4.1E+07	6.5E-01	1.5E+00	8.7E-03
LMRG_01817	lmo2431	HupD	5.5E+07	4.8E+07	8.8E-01	1.1E+00	1.0E-01
LMRG_01819	lmo2429	HupC	2.3E+07	1.8E+07	7.8E-01	1.3E+00	7.1E-02
LMRG_01821	lmo2427	RodA1	5.5E+06	8.8E+06	1.6E+00	6.3E-01	5.7E-01
LMRG_01822	lmo2426	SpxA2	1.2E+08	1.6E+07	1.3E-01	7.8E+00	5.7E-04
LMRG_01823	lmo2425	GcvH	1.8E+08	3.0E+07	1.7E-01	5.9E+00	7.0E-05
LMRG_01824	lmo2424		2.8E+06	0.0E+00	0.0E+00	#DIV/0!	1.2E-01
LMRG_01825	lmo2423		0.0E+00	6.4E+05	#DIV/0!	0.0E+00	3.7E-01
LMRG_01826	lmo2422	CesR	1.7E+07	1.4E+07	8.0E-01	1.2E+00	2.0E-02
LMRG_01827	lmo2421	CesK	1.2E+07	1.2E+07	9.6E-01	1.0E+00	7.7E-01
LMRG_01829	lmo2419	MetN	7.6E+07	9.8E+07	1.3E+00	7.7E-01	1.0E-02
LMRG_01830	lmo2418		1.5E+07	2.2E+07	1.4E+00	6.9E-01	2.3E-02
LMRG_01831	lmo2417		3.2E+08	3.0E+08	9.3E-01	1.1E+00	5.9E-01
LMRG_01832	lmo2416		1.9E+07	2.3E+07	1.3E+00	7.9E-01	5.8E-01
LMRG_01833	lmo2415	SufC	4.1E+08	3.4E+08	8.1E-01	1.2E+00	1.7E-01
LMRG_01834	lmo2414	SufD	3.3E+08	2.6E+08	8.0E-01	1.3E+00	1.0E-01
LMRG_01835	lmo2413		2.2E+07	1.5E+07	6.7E-01	1.5E+00	1.9E-01
LMRG_01836	lmo2412	SufU	4.2E+06	3.3E+06	7.9E-01	1.3E+00	8.7E-01
LMRG_01837	lmo2411	SufB	2.3E+08	1.7E+08	7.4E-01	1.3E+00	5.8E-02
LMRG_01841	lmo2407		3.6E+07	7.4E+07	2.1E+00	4.8E-01	1.0E-02
LMRG_01842	lmo2406		2.9E+07	0.0E+00	0.0E+00	#DIV/0!	1.3E-06
LMRG_01844	lmo2404		2.0E+06	0.0E+00	0.0E+00	#DIV/0!	3.7E-01
LMRG_01845	lmo2403		5.5E+07	1.2E+07	2.1E-01	4.7E+00	2.3E-02
LMRG_01846	lmo2402		4.4E+07	3.8E+07	8.5E-01	1.2E+00	3.8E-01
LMRG_01847	lmo2401		1.8E+08	1.9E+08	1.1E+00	9.5E-01	6.3E-01
LMRG_01850	lmo2398	LtrC	7.6E+06	8.1E+06	1.1E+00	9.4E-01	1.6E-01
LMRG_01851	lmo2397		1.3E+07	4.3E+06	3.3E-01	3.1E+00	1.1E-01
LMRG_01853	lmo2845	MdrB	1.1E+06	0.0E+00	0.0E+00	#DIV/0!	3.7E-01
LMRG_01854	lmo2844		9.1E+05	0.0E+00	0.0E+00	#DIV/0!	3.7E-01
LMRG_01855	lmo2843		1.7E+07	1.3E+07	7.7E-01	1.3E+00	1.1E-01
LMRG_01856	lmo2842		1.4E+07	1.0E+07	7.2E-01	1.4E+00	1.3E-02
LMRG_01867	lmo2831	PgmB	5.5E+07	5.4E+07	9.8E-01	1.0E+00	5.6E-01
LMRG_01869	lmo2829		1.6E+08	8.8E+07	5.6E-01	1.8E+00	4.0E-03
LMRG_01871	lmo2827		1.4E+06	2.9E+06	2.0E+00	5.0E-01	5.3E-01
LMRG_01873	lmo2825	SerC	2.8E+07	0.0E+00	0.0E+00	#DIV/0!	2.7E-05
LMRG_01875	lmo2823		4.2E+07	2.3E+07	5.4E-01	1.8E+00	9.8E-04
LMRG_01878	lmo2820		1.4E+07	6.7E+07	4.7E+00	2.1E-01	3.3E-02
LMRG_01883	lmo2815		5.9E+06	4.6E+06	7.8E-01	1.3E+00	6.2E-01
LMRG_01884	lmo2814		4.7E+06	2.6E+06	5.5E-01	1.8E+00	5.8E-01
LMRG_01887	lmo2811	MnmE	7.7E+07	8.0E+07	1.0E+00	9.6E-01	6.9E-01
LMRG_01888	lmo2810	MnmG	2.4E+08	2.3E+08	9.5E-01	1.0E+00	5.2E-01
LMRG_01889	lmo2807		0.0E+00	9.4E+05	#DIV/0!	0.0E+00	3.7E-01
LMRG_01891	lmo2805		3.0E+07	4.4E+07	1.5E+00	6.7E-01	2.4E-03
LMRG_01892	lmo2804		2.7E+06	1.4E+07	5.2E+00	1.9E-01	2.6E-02
LMRG_01895	lmo2802	RsmG	1.1E+08	1.7E+08	1.6E+00	6.3E-01	1.0E-03
LMRG_01896	lmo2801	NanE	0.0E+00	9.1E+06	#DIV/0!	0.0E+00	8.7E-04
LMRG_01897	lmo2800		2.2E+06	1.1E+07	4.9E+00	2.0E-01	1.4E-02
LMRG_01903	lmo2794	Noc	5.9E+07	5.6E+07	9.6E-01	1.0E+00	3.9E-01
LMRG_01905	lmo2792		5.3E+08	2.5E+08	4.8E-01	2.1E+00	2.3E-03
LMRG_01906	lmo2791	ParA	1.1E+08	9.9E+07	9.1E-01	1.1E+00	9.3E-02
LMRG_01907	lmo2790	ParB	2.0E+08	1.5E+08	7.6E-01	1.3E+00	2.5E-03
LMRG_01908	lmo2789		9.0E+06	4.6E+06	5.1E-01	2.0E+00	1.7E-01
LMRG_01909	lmo2788	BvrA	1.6E+06	4.3E+06	2.8E+00	3.6E-01	3.9E-01
LMRG_01912	lmo2785	Kat	2.8E+08	5.6E+06	2.0E-02	5.0E+01	1.4E-02
LMRG_01918	lmo2779	YchF	9.0E+08	1.4E+09	1.5E+00	6.5E-01	2.8E-03
LMRG_01923	lmo2772		0.0E+00	4.5E+05	#DIV/0!	0.0E+00	3.7E-01
LMRG_01925	lmo2770	GshF	3.2E+08	2.5E+07	7.9E-02	1.3E+01	6.0E-02
LMRG_01926	lmo2769	EslA	0.0E+00	4.7E+05	#DIV/0!	0.0E+00	3.7E-01
LMRG_01928	lmo2767	EslC	1.3E+08	2.2E+08	1.7E+00	5.9E-01	3.5E-02

LMRG_01929	lmo2766	EslR	1.0E+07	1.4E+07	1.4E+00	7.4E-01	4.3E-02
LMRG_01936	lmo2760		4.7E+06	3.3E+06	7.1E-01	1.4E+00	8.3E-01
LMRG_01937	lmo2759		7.4E+07	3.2E+07	4.4E-01	2.3E+00	4.4E-03
LMRG_01938	lmo2758	GuaB	2.3E+09	3.4E+09	1.5E+00	6.7E-01	1.2E-03
LMRG_01939	lmo2757	RecQ	3.8E+07	4.7E+07	1.2E+00	8.1E-01	6.4E-03
LMRG_01940	lmo2756	TopB	2.9E+08	2.8E+08	9.7E-01	1.0E+00	6.3E-01
LMRG_01942	lmo2754	PbpD1	3.0E+08	4.0E+08	1.4E+00	7.4E-01	1.1E-03
LMRG_01944	lmo2752		4.8E+07	1.7E+08	3.5E+00	2.9E-01	2.8E-03
LMRG_01945	lmo2751		8.0E+07	2.6E+08	3.2E+00	3.1E-01	1.6E-03
LMRG_01946	lmo2750	PabB	0.0E+00	1.3E+06	#DIV/0!	0.0E+00	3.7E-01
LMRG_01947	lmo2749		0.0E+00	2.0E+06	#DIV/0!	0.0E+00	3.7E-01
LMRG_01948	lmo2748		0.0E+00	5.8E+06	#DIV/0!	0.0E+00	1.2E-01
LMRG_01949	lmo2747	SerS	7.1E+08	6.9E+08	9.6E-01	1.0E+00	3.5E-01
LMRG_01950	lmo2746		2.8E+06	1.5E+06	5.3E-01	1.9E+00	5.6E-01
LMRG_01951	lmo2745		2.2E+07	2.2E+07	1.0E+00	9.9E-01	9.1E-01
LMRG_01953	lmo2743	Tal	2.2E+06	0.0E+00	0.0E+00	#DIV/0!	1.3E-01
LMRG_01957	lmo2739	CobB	1.3E+07	4.5E+06	3.5E-01	2.9E+00	1.4E-01
LMRG_01958	lmo2738		1.8E+07	2.1E+07	1.2E+00	8.7E-01	9.5E-02
LMRG_01959	lmo2737		2.4E+06	0.0E+00	0.0E+00	#DIV/0!	1.2E-01
LMRG_01966	lmo2730		1.4E+06	4.0E+06	2.9E+00	3.4E-01	3.4E-01
LMRG_01969	lmo2727		8.5E+06	0.0E+00	0.0E+00	#DIV/0!	1.1E-04
LMRG_01970	lmo2726		4.7E+06	2.9E+06	6.2E-01	1.6E+00	2.9E-01
LMRG_01971	lmo2725		5.2E+05	0.0E+00	0.0E+00	#DIV/0!	3.7E-01
LMRG_01972	lmo2724		4.4E+07	4.1E+07	9.3E-01	1.1E+00	4.8E-01
LMRG_01973	lmo2723		1.3E+07	2.5E+06	2.0E-01	5.1E+00	1.6E-02
LMRG_01976	lmo2720		2.0E+08	5.6E+06	2.8E-02	3.6E+01	4.9E-05
LMRG_01977	lmo2719	TadA	1.7E+07	0.0E+00	0.0E+00	#DIV/0!	7.4E-06
LMRG_01978	lmo2718	CydA	6.3E+07	4.9E+06	7.7E-02	1.3E+01	9.2E-04
LMRG_01980	lmo2716	CydC	5.7E+07	0.0E+00	0.0E+00	#DIV/0!	1.5E-06
LMRG_01981	lmo2715	CydD	5.5E+07	2.0E+06	3.6E-02	2.7E+01	1.3E-03
LMRG_01982	lmo2714		0.0E+00	2.8E+06	#DIV/0!	0.0E+00	3.7E-01
LMRG_01984	lmo2712		4.0E+05	0.0E+00	0.0E+00	#DIV/0!	3.7E-01
LMRG_01987	lmo2710		3.0E+07	3.6E+07	1.2E+00	8.3E-01	3.3E-02
LMRG_01988	lmo2709		1.3E+07	9.0E+06	6.8E-01	1.5E+00	3.4E-02
LMRG_01990	lmo2707		2.5E+08	2.2E+08	8.6E-01	1.2E+00	1.6E-01
LMRG_01992	lmo2705		3.7E+07	2.8E+07	7.7E-01	1.3E+00	2.1E-01
LMRG_01993	lmo2704	DnaX	9.3E+07	8.5E+07	9.1E-01	1.1E+00	6.7E-02
LMRG_01994	lmo2703		3.8E+08	3.1E+08	8.0E-01	1.2E+00	1.9E-02
LMRG_01995	lmo2702	RecR	4.3E+07	4.9E+07	1.1E+00	8.8E-01	3.0E-02
LMRG_01996	lmo2701		1.5E+07	1.5E+07	1.0E+00	1.0E+00	9.8E-01
LMRG_01997	lmo2700		3.3E+08	2.3E+08	7.0E-01	1.4E+00	1.1E-02
LMRG_01999	lmo2698		1.1E+06	1.2E+06	1.1E+00	9.0E-01	9.4E-01
LMRG_02000	lmo2697		2.3E+07	3.1E+07	1.3E+00	7.5E-01	1.9E-01
LMRG_02001	lmo2696	DhaL	8.4E+07	7.1E+07	8.4E-01	1.2E+00	2.7E-01
LMRG_02002	lmo2695	DhaK	4.5E+07	5.0E+07	1.1E+00	9.0E-01	2.4E-01
LMRG_02003	lmo0903		6.3E+07	5.7E+07	9.1E-01	1.1E+00	6.6E-01
LMRG_02004	lmo0904		1.3E+07	5.4E+06	4.3E-01	2.3E+00	7.1E-02
LMRG_02006	lmo0906	GorA	1.8E+08	9.5E+07	5.3E-01	1.9E+00	8.7E-04
LMRG_02007	lmo0907		1.3E+08	6.0E+07	4.8E-01	2.1E+00	1.0E-03
LMRG_02008	lmo0908		2.6E+06	0.0E+00	0.0E+00	#DIV/0!	3.7E-01
LMRG_02009	lmo0909		0.0E+00	5.0E+05	#DIV/0!	0.0E+00	3.7E-01
LMRG_02012	lmo0912	NirC	3.5E+07	4.7E+07	1.3E+00	7.5E-01	1.3E-01
LMRG_02013	lmo0913		7.5E+07	8.4E+07	1.1E+00	8.9E-01	4.2E-01
LMRG_02017	lmo0917		0.0E+00	1.5E+06	#DIV/0!	0.0E+00	3.7E-01
LMRG_02021	lmo0921		2.1E+06	2.1E+06	1.0E+00	9.7E-01	9.8E-01
LMRG_02022	lmo0922	CoaA	2.4E+07	3.0E+07	1.3E+00	7.9E-01	1.6E-02
LMRG_02023	lmo0923		9.2E+05	0.0E+00	0.0E+00	#DIV/0!	3.7E-01
LMRG_02024	lmo0925		3.8E+06	3.4E+06	8.9E-01	1.1E+00	3.6E-01
LMRG_02025	lmo0926		6.6E+06	6.8E+06	1.0E+00	9.7E-01	8.4E-01
LMRG_02026	lmo0927	LtaS	1.0E+08	1.2E+08	1.1E+00	9.0E-01	5.8E-01
LMRG_02028	lmo0929	SrtA	1.9E+08	1.0E+08	5.2E-01	1.9E+00	1.6E-03
LMRG_02029	lmo0930		6.5E+07	3.9E+07	6.1E-01	1.6E+00	1.3E-02
LMRG_02030	lmo0931	LplA1	1.4E+08	6.2E+07	4.5E-01	2.2E+00	5.2E-03
LMRG_02032	lmo0933	GtlA	6.7E+07	6.3E+07	9.4E-01	1.1E+00	5.7E-01
LMRG_02033	lmo0934	QueG	1.3E+07	7.5E+06	5.7E-01	1.7E+00	3.6E-02
LMRG_02034	lmo0935	TrmL	1.1E+08	1.2E+08	1.1E+00	9.3E-01	3.7E-01
LMRG_02035	lmo0936	NfsA	3.5E+07	3.0E+07	8.7E-01	1.1E+00	3.1E-01
LMRG_02037	lmo0938		3.1E+07	1.9E+07	6.0E-01	1.7E+00	1.5E-02
LMRG_02040	lmo0942		1.4E+08	2.4E+08	1.7E+00	5.8E-01	1.0E-03
LMRG_02041	lmo0943	Fri	2.2E+08	2.6E+08	1.2E+00	8.3E-01	2.2E-01
LMRG_02042	lmo0944		2.7E+06	0.0E+00	0.0E+00	#DIV/0!	3.7E-01
LMRG_02045	lmo0946		0.0E+00	1.9E+06	#DIV/0!	0.0E+00	3.7E-01
LMRG_02047	lmo0948		1.1E+07	1.4E+07	1.3E+00	8.0E-01	3.3E-01
LMRG_02049	lmo0950		2.7E+07	1.8E+07	6.5E-01	1.5E+00	1.7E-02
LMRG_02050	lmo0951		1.3E+07	0.0E+00	0.0E+00	#DIV/0!	4.9E-04
LMRG_02051	lmo0952		1.9E+07	2.6E+07	1.4E+00	7.3E-01	9.3E-03
LMRG_02052	lmo0953		1.0E+07	1.2E+07	1.2E+00	8.2E-01	2.9E-01
LMRG_02053	lmo0954		2.2E+06	0.0E+00	0.0E+00	#DIV/0!	3.7E-01
LMRG_02054	lmo0955		3.3E+08	6.0E+08	1.8E+00	5.5E-01	1.3E-02
LMRG_02055	lmo0956	NagA	2.5E+08	4.1E+08	1.6E+00	6.2E-01	4.6E-03
LMRG_02056	lmo0957	NagB	3.5E+08	6.4E+08	1.8E+00	5.4E-01	6.5E-03
LMRG_02057	lmo0958		4.4E+07	3.6E+07	8.2E-01	1.2E+00	6.2E-03
LMRG_02059	lmo0960		1.5E+08	2.1E+08	1.4E+00	6.9E-01	6.5E-03
LMRG_02060	lmo0961		2.8E+08	3.6E+08	1.3E+00	7.8E-01	5.7E-04
LMRG_02061	lmo0962	LemA	5.2E+08	4.8E+08	9.2E-01	1.1E+00	2.0E-01
LMRG_02062	lmo0963	HtpX	3.5E+07	3.5E+07	1.0E+00	1.0E+00	9.9E-01

LMRG_02064	lmo0965		2.4E+07	7.4E+06	3.1E-01	3.3E+00	1.8E-02
LMRG_02065	lmo0966		6.2E+06	0.0E+00	0.0E+00	#DIV/0!	1.2E-01
LMRG_02066	lmo0967		5.2E+07	3.9E+07	7.6E-01	1.3E+00	9.8E-03
LMRG_02067	lmo0968	NadK	2.6E+07	2.1E+07	8.1E-01	1.2E+00	1.5E-01
LMRG_02068	lmo0969		4.9E+06	0.0E+00	0.0E+00	#DIV/0!	1.2E-01
LMRG_02069	lmo0970	FabI	8.4E+08	6.6E+08	7.8E-01	1.3E+00	3.2E-02
LMRG_02070	lmo0971	DltD	3.2E+08	3.8E+08	1.2E+00	8.5E-01	2.6E-01
LMRG_02071	lmo0972	DltC	6.8E+08	8.4E+08	1.2E+00	8.1E-01	4.4E-01
LMRG_02073	lmo0974	DltA	5.9E+08	5.3E+08	8.9E-01	1.1E+00	5.3E-01
LMRG_02075	lmo0975	RpiA	0.0E+00	1.1E+06	#DIV/0!	0.0E+00	3.7E-01
LMRG_02077	lmo0977		1.3E+07	1.4E+07	1.1E+00	9.3E-01	6.5E-01
LMRG_02078	lmo0978		7.9E+08	7.7E+08	9.7E-01	1.0E+00	4.7E-01
LMRG_02079	lmo0979	LieA	1.2E+06	0.0E+00	0.0E+00	#DIV/0!	3.7E-01
LMRG_02080	lmo0980	LieB	1.8E+05	0.0E+00	0.0E+00	#DIV/0!	3.7E-01
LMRG_02081	lmo0981		0.0E+00	9.7E+05	#DIV/0!	0.0E+00	3.7E-01
LMRG_02082	lmo0982		1.8E+08	6.8E+07	3.9E-01	2.6E+00	5.6E-05
LMRG_02083	lmo0983		4.2E+07	4.9E+06	1.1E-01	8.7E+00	6.0E-03
LMRG_02088	lmo0988	PrfC	5.7E+08	7.1E+08	1.2E+00	8.0E-01	3.5E-02
LMRG_02089	lmo0989		5.9E+06	4.1E+06	6.9E-01	1.4E+00	4.6E-01
LMRG_02097	lmo0997	ClpE	2.3E+08	2.3E+09	9.9E+00	1.0E-01	1.1E-03
LMRG_02101	lmo1001		1.8E+07	0.0E+00	0.0E+00	#DIV/0!	1.2E-01
LMRG_02102	lmo1002	PtsH	7.1E+09	6.8E+09	9.7E-01	1.0E+00	7.5E-01
LMRG_02103	lmo1003	PtsI	6.8E+09	9.3E+09	1.4E+00	7.3E-01	2.2E-03
LMRG_02104	lmo1004		1.0E+06	0.0E+00	0.0E+00	#DIV/0!	3.7E-01
LMRG_02105	lmo1005		2.1E+07	1.7E+07	8.3E-01	1.2E+00	1.9E-02
LMRG_02106	lmo1006		6.9E+07	8.7E+07	1.3E+00	7.9E-01	9.7E-02
LMRG_02108	lmo1008		3.6E+08	4.1E+08	1.1E+00	8.9E-01	2.0E-01
LMRG_02109	lmo1009	CbpB	3.9E+07	3.5E+07	9.2E-01	1.1E+00	4.2E-01
LMRG_02110	lmo1010	CcpC	2.4E+07	1.4E+07	5.6E-01	1.8E+00	5.7E-03
LMRG_02111	lmo1011	DapH	6.1E+08	3.3E+08	5.3E-01	1.9E+00	9.1E-04
LMRG_02112	lmo1012		1.0E+08	7.1E+07	7.0E-01	1.4E+00	5.1E-03
LMRG_02113	lmo1013		6.0E+07	5.0E+07	8.4E-01	1.2E+00	2.5E-01
LMRG_02114	lmo1014	GbuA	2.7E+08	1.0E+08	3.8E-01	2.7E+00	7.3E-03
LMRG_02115	lmo1015	GbuB	1.7E+07	9.3E+06	5.3E-01	1.9E+00	1.2E-01
LMRG_02116	lmo1016	GbuC	2.9E+07	1.1E+07	3.9E-01	2.6E+00	7.0E-02
LMRG_02117	lmo1017		1.5E+08	4.4E+07	3.0E-01	3.3E+00	3.4E-04
LMRG_02118	lmo1018	CutC	5.9E+07	6.6E+07	1.1E+00	8.9E-01	2.9E-01
LMRG_02119	lmo1019		1.2E+08	1.7E+08	1.4E+00	7.1E-01	1.9E-03
LMRG_02120	lmo1020	LiaF	6.0E+06	4.5E+06	7.4E-01	1.3E+00	5.5E-01
LMRG_02121	lmo1021	LiaS	1.1E+07	1.7E+07	1.6E+00	6.1E-01	3.0E-03
LMRG_02122	lmo1022	LiaR	8.3E+07	9.0E+07	1.1E+00	9.2E-01	2.6E-01
LMRG_02123	lmo1023		1.8E+07	2.1E+07	1.2E+00	8.5E-01	1.7E-01
LMRG_02124	lmo1024		0.0E+00	1.8E+05	#DIV/0!	0.0E+00	3.7E-01
LMRG_02125	lmo1025		1.4E+06	8.1E+05	5.7E-01	1.8E+00	7.3E-01
LMRG_02126	lmo1026		2.6E+08	2.5E+08	9.6E-01	1.0E+00	3.8E-01
LMRG_02127	lmo1027	Rnj	7.0E+08	6.8E+08	9.7E-01	1.0E+00	5.2E-01
LMRG_02128	lmo1028	RpoY	8.3E+08	6.8E+08	8.2E-01	1.2E+00	6.9E-02
LMRG_02129	lmo1029		1.6E+08	2.3E+08	1.5E+00	6.9E-01	1.8E-02
LMRG_02130	lmo2590		4.6E+06	0.0E+00	0.0E+00	#DIV/0!	6.2E-05
LMRG_02132	lmo2592		2.7E+07	2.2E+07	8.1E-01	1.2E+00	1.2E-01
LMRG_02135		Csn2	6.5E+05	0.0E+00	0.0E+00	#DIV/0!	3.7E-01
LMRG_02137		Cas1	4.5E+05	0.0E+00	0.0E+00	#DIV/0!	3.7E-01
LMRG_02138		Cas9	9.2E+05	0.0E+00	0.0E+00	#DIV/0!	3.7E-01
LMRG_02140	lmo2596	RpsI	5.0E+09	5.5E+09	1.1E+00	9.0E-01	5.3E-02
LMRG_02141	lmo2597	RplM	3.4E+09	4.0E+09	1.2E+00	8.5E-01	4.7E-02
LMRG_02142	lmo2598	TruA	4.2E+07	3.8E+07	8.9E-01	1.1E+00	2.9E-01
LMRG_02143	lmo2599	EcfT	0.0E+00	1.1E+06	#DIV/0!	0.0E+00	3.7E-01
LMRG_02144	lmo2600	EcfA	8.6E+07	8.6E+07	1.0E+00	1.0E+00	1.0E+00
LMRG_02145	lmo2601	EcfA	7.9E+07	6.9E+07	8.7E-01	1.1E+00	3.2E-01
LMRG_02149	lmo2605	RplQ	4.0E+09	3.7E+09	9.2E-01	1.1E+00	2.7E-01
LMRG_02150	lmo2606	RpoA	2.9E+09	2.8E+09	9.9E-01	1.0E+00	8.2E-01
LMRG_02151	lmo2607	RpsK	2.3E+09	2.3E+09	9.8E-01	1.0E+00	6.9E-01
LMRG_02152	lmo2608	RpsM	3.2E+09	3.0E+09	9.4E-01	1.1E+00	1.7E-01
LMRG_02153	lmo2609	RpmJ	5.8E+08	1.3E+09	2.2E+00	4.5E-01	1.1E-01
LMRG_02155	lmo2611	Adk	1.6E+09	1.6E+09	9.9E-01	1.0E+00	8.3E-01
LMRG_02156	lmo2612	SecY	5.1E+07	4.8E+07	9.5E-01	1.0E+00	4.5E-01
LMRG_02157	lmo2613	RplO	3.8E+09	4.0E+09	1.0E+00	9.6E-01	6.1E-01
LMRG_02158	lmo2614	RpmD	3.9E+09	3.8E+09	9.6E-01	1.0E+00	6.9E-01
LMRG_02159	lmo2615	RpsE	5.4E+09	4.6E+09	8.5E-01	1.2E+00	8.4E-02
LMRG_02160	lmo2616	RplR	3.6E+09	3.5E+09	9.6E-01	1.0E+00	7.3E-01
LMRG_02161	lmo2617	RplF	6.5E+09	6.7E+09	1.0E+00	9.7E-01	6.7E-01
LMRG_02162	lmo2618	RpsH	4.5E+09	4.3E+09	9.5E-01	1.1E+00	5.8E-01
LMRG_02163	lmo2619	RpsZ	2.4E+08	4.9E+08	2.1E+00	4.8E-01	3.3E-02
LMRG_02164	lmo2620	RplE	9.9E+09	1.0E+10	1.0E+00	9.7E-01	5.1E-01
LMRG_02165	lmo2621	RplX	1.5E+09	1.7E+09	1.1E+00	8.8E-01	1.4E-01
LMRG_02166	lmo2622	RplN	3.9E+09	3.4E+09	8.7E-01	1.1E+00	8.7E-02
LMRG_02167	lmo2623	RpsQ	2.0E+09	1.7E+09	8.6E-01	1.2E+00	2.9E-01
LMRG_02168	lmo2624	RpmC	4.3E+09	4.1E+09	9.5E-01	1.1E+00	3.5E-01
LMRG_02169	lmo2625	RplP	2.0E+09	2.3E+09	1.1E+00	8.8E-01	8.1E-02
LMRG_02170	lmo2626	RpsC	7.6E+09	8.4E+09	1.1E+00	9.1E-01	7.9E-02
LMRG_02171	lmo2627	RplV	3.4E+09	3.7E+09	1.1E+00	9.1E-01	3.3E-01
LMRG_02172	lmo2628	RpsS	3.6E+09	4.0E+09	1.1E+00	8.9E-01	2.2E-01
LMRG_02173	lmo2629	RplB	9.4E+09	1.1E+10	1.2E+00	8.3E-01	1.9E-02
LMRG_02174	lmo2630	RplW	3.0E+09	2.5E+09	8.4E-01	1.2E+00	2.5E-03
LMRG_02175	lmo2631	RplD	6.0E+09	6.6E+09	1.1E+00	9.0E-01	3.5E-02
LMRG_02176	lmo2632	RplC	4.2E+09	4.7E+09	1.1E+00	8.9E-01	1.0E-01

LMRG_02177	lmo2633	RpsJ	5.2E+09	4.5E+09	8.6E-01	1.2E+00	1.8E-01
LMRG_02179	lmo2634	EcfT	2.0E+07	2.3E+07	1.2E+00	8.6E-01	2.6E-01
LMRG_02180	lmo2635	MenA	2.2E+07	2.2E+07	9.9E-01	1.0E+00	9.6E-01
LMRG_02181	lmo2636		4.7E+08	8.0E+08	1.7E+00	5.9E-01	5.9E-06
LMRG_02182	lmo2637	PplA	1.2E+10	1.7E+10	1.4E+00	7.3E-01	4.0E-03
LMRG_02183	lmo2638		3.4E+09	4.2E+09	1.2E+00	8.2E-01	1.2E-02
LMRG_02184	lmo2639	EetA	6.3E+06	1.1E+07	1.7E+00	5.8E-01	2.2E-01
LMRG_02186	lmo2641		4.3E+07	5.3E+07	1.2E+00	8.1E-01	1.4E-02
LMRG_02187	lmo2642		2.8E+06	3.0E+06	1.0E+00	9.6E-01	9.8E-01
LMRG_02188	lmo2643		4.7E+07	3.1E+07	6.6E-01	1.5E+00	3.0E-03
LMRG_02196	lmo2651		0.0E+00	3.1E+05	#DIV/0!	0.0E+00	3.7E-01
LMRG_02198	lmo2653	Tuf	3.9E+10	4.6E+10	1.2E+00	8.5E-01	1.3E-01
LMRG_02199	lmo2654	FusA	1.5E+10	1.8E+10	1.2E+00	8.4E-01	2.0E-02
LMRG_02200	lmo2655	RpsG	5.8E+09	6.3E+09	1.1E+00	9.1E-01	3.3E-01
LMRG_02201	lmo2656	RpsL	1.4E+09	3.9E+09	2.7E+00	3.7E-01	3.8E-03
LMRG_02202	lmo2657		1.4E+08	1.2E+08	8.4E-01	1.2E+00	2.0E-02
LMRG_02203	lmo2658		6.2E+07	7.3E+07	1.2E+00	8.6E-01	1.9E-01
LMRG_02205	lmo2660	Tkt	0.0E+00	1.5E+07	#DIV/0!	0.0E+00	5.1E-05
LMRG_02208	lmo2663		0.0E+00	2.7E+06	#DIV/0!	0.0E+00	3.7E-01
LMRG_02211	lmo2666		1.5E+07	2.2E+07	1.4E+00	7.1E-01	9.2E-02
LMRG_02212	lmo2667		1.1E+07	4.4E+07	3.9E+00	2.6E-01	5.3E-02
LMRG_02214	lmo2669		0.0E+00	1.1E+06	#DIV/0!	0.0E+00	3.7E-01
LMRG_02216	lmo2671		0.0E+00	8.8E+05	#DIV/0!	0.0E+00	3.7E-01
LMRG_02218	lmo2673		4.3E+07	1.6E+07	3.6E-01	2.8E+00	7.8E-04
LMRG_02219	lmo2674	RpiB	2.1E+08	1.5E+08	7.1E-01	1.4E+00	1.4E-02
LMRG_02220	lmo2675		0.0E+00	4.0E+06	#DIV/0!	0.0E+00	3.7E-01
LMRG_02222	lmo2677		6.7E+07	5.0E+07	7.5E-01	1.3E+00	2.7E-02
LMRG_02223	lmo2678	KdpE	6.2E+05	0.0E+00	0.0E+00	#DIV/0!	3.7E-01
LMRG_02228	lmo2683		1.5E+08	1.4E+08	9.2E-01	1.1E+00	6.4E-01
LMRG_02229	lmo2684		7.8E+07	6.6E+07	8.5E-01	1.2E+00	4.7E-01
LMRG_02230	lmo2685		1.6E+08	1.7E+08	1.1E+00	9.2E-01	7.6E-01
LMRG_02237	lmo2690		3.1E+07	2.7E+07	8.8E-01	1.1E+00	3.5E-01
LMRG_02238	lmo2691	NamA	5.3E+08	9.0E+08	1.7E+00	5.9E-01	3.8E-02
LMRG_02239	lmo2692	PstA	1.1E+08	2.4E+07	2.1E-01	4.7E+00	7.2E-07
LMRG_02240	lmo2693	Tmk	2.0E+08	2.2E+08	1.1E+00	9.2E-01	2.3E-01
LMRG_02241	lmo2694		2.9E+07	1.3E+07	4.7E-01	2.1E+00	4.6E-04
LMRG_02243	lmo0818		8.0E+05	0.0E+00	0.0E+00	#DIV/0!	3.7E-01
LMRG_02244	lmo0819		5.8E+06	5.9E+06	1.0E+00	9.9E-01	9.5E-01
LMRG_02245	lmo0820		3.1E+07	2.8E+07	9.0E-01	1.1E+00	2.9E-01
LMRG_02248	lmo0823		3.6E+07	0.0E+00	0.0E+00	#DIV/0!	2.0E-03
LMRG_02249	lmo0824		2.7E+06	1.1E+06	4.2E-01	2.4E+00	4.3E-01
LMRG_02250	lmo0825	HmgR	5.7E+07	6.2E+07	1.1E+00	9.3E-01	2.5E-01
LMRG_02251	lmo0826		4.5E+07	5.0E+07	1.1E+00	9.0E-01	3.6E-01
LMRG_02252	lmo0829	NifJ	2.7E+08	2.2E+08	8.1E-01	1.2E+00	1.7E-02
LMRG_02260	lmo0837		6.7E+05	0.0E+00	0.0E+00	#DIV/0!	3.7E-01
LMRG_02263	lmo0840		8.2E+06	8.0E+06	9.8E-01	1.0E+00	8.9E-01
LMRG_02264	lmo0841		5.8E+07	9.0E+06	1.5E-01	6.5E+00	3.5E-03
LMRG_02265	lmo0842		0.0E+00	1.9E+06	#DIV/0!	0.0E+00	3.7E-01
LMRG_02267	lmo0844		1.1E+07	0.0E+00	0.0E+00	#DIV/0!	3.9E-06
LMRG_02268	lmo0845		2.5E+07	2.2E+07	8.8E-01	1.1E+00	2.7E-01
LMRG_02270	lmo0847	GlnP	2.5E+08	4.2E+08	1.7E+00	5.8E-01	1.1E-04
LMRG_02271	lmo0848	GlnQ	3.0E+08	4.8E+08	1.6E+00	6.3E-01	2.5E-03
LMRG_02274	lmo0851		4.5E+07	5.1E+07	1.1E+00	8.8E-01	3.2E-01
LMRG_02275	lmo0852	SugR	2.9E+07	2.5E+07	8.5E-01	1.2E+00	5.0E-02
LMRG_02278	lmo0855	Ddl	7.0E+08	8.2E+08	1.2E+00	8.5E-01	4.9E-02
LMRG_02279	lmo0856	MurF	2.7E+08	4.0E+08	1.5E+00	6.7E-01	7.7E-04
LMRG_02280	lmo0857		3.4E+07	3.3E+07	9.8E-01	1.0E+00	8.7E-01
LMRG_02281	lmo0858		1.1E+07	4.7E+06	4.4E-01	2.3E+00	1.1E-01
LMRG_02282	lmo0859		1.7E+07	4.3E+07	2.5E+00	4.1E-01	1.6E-01
LMRG_02286	lmo0863		0.0E+00	4.2E+06	#DIV/0!	0.0E+00	1.2E-01
LMRG_02287	lmo0864		0.0E+00	2.0E+07	#DIV/0!	0.0E+00	9.9E-03
LMRG_02289	lmo0866	CshA	3.4E+09	4.4E+09	1.3E+00	7.7E-01	3.7E-02
LMRG_02294	lmo0870		1.1E+05	0.0E+00	0.0E+00	#DIV/0!	3.7E-01
LMRG_02297	lmo0873		0.0E+00	9.2E+05	#DIV/0!	0.0E+00	3.7E-01
LMRG_02306	lmo0882		1.9E+07	1.8E+07	9.4E-01	1.1E+00	3.4E-01
LMRG_02307	lmo0883		2.7E+07	2.5E+07	9.3E-01	1.1E+00	2.3E-01
LMRG_02308	lmo0884		4.2E+07	4.2E+07	1.0E+00	1.0E+00	9.6E-01
LMRG_02309	lmo0885	AcpS	3.3E+06	5.1E+06	1.6E+00	6.4E-01	3.4E-01
LMRG_02310	lmo0886	Alr	1.5E+07	1.2E+07	8.3E-01	1.2E+00	2.3E-01
LMRG_02311	lmo0887	MazE	1.6E+06	0.0E+00	0.0E+00	#DIV/0!	3.7E-01
LMRG_02312	lmo0888	MazF	4.7E+07	3.7E+07	7.9E-01	1.3E+00	1.2E-02
LMRG_02313	lmo0889	RsbR	1.7E+08	1.9E+08	1.1E+00	9.1E-01	1.9E-01
LMRG_02314	lmo0890	RsbS	3.5E+07	2.3E+07	6.7E-01	1.5E+00	1.3E-01
LMRG_02315	lmo0891	RsbT	1.2E+08	8.5E+07	7.2E-01	1.4E+00	5.9E-03
LMRG_02316	lmo0892	RsbU	2.8E+07	2.2E+07	7.8E-01	1.3E+00	1.3E-02
LMRG_02317	lmo0893	RsbV	1.2E+08	1.1E+08	9.9E-01	1.0E+00	9.5E-01
LMRG_02318	lmo0894	RsbW	1.7E+08	1.8E+08	1.1E+00	9.2E-01	4.8E-01
LMRG_02319	lmo0895	SigB	6.5E+07	7.2E+07	1.1E+00	9.0E-01	3.2E-01
LMRG_02320	lmo0896	RsbX	7.5E+06	3.4E+06	4.5E-01	2.2E+00	7.2E-02
LMRG_02321	lmo0897		2.7E+07	3.5E+07	1.3E+00	7.9E-01	2.8E-02
LMRG_02322	lmo0898		4.2E+08	3.7E+08	8.9E-01	1.1E+00	2.4E-01
LMRG_02328	lmo0077		2.7E+07	3.8E+07	1.4E+00	7.2E-01	1.1E-02
LMRG_02329	lmo0078		7.3E+07	6.4E+07	8.8E-01	1.1E+00	2.1E-01
LMRG_02331	lmo0083		4.1E+05	0.0E+00	0.0E+00	#DIV/0!	3.7E-01
LMRG_02332	lmo0084		1.3E+07	5.5E+06	4.4E-01	2.3E+00	1.0E-01
LMRG_02345	lmo0096		4.3E+09	3.6E+09	8.4E-01	1.2E+00	1.6E-02

LMRG_02346	lmo0097		2.5E+08	2.1E+08	8.5E-01	1.2E+00	1.5E-01
LMRG_02347	lmo0098		2.7E+09	2.1E+09	7.9E-01	1.3E+00	2.1E-03
LMRG_02348	lmo0099		3.8E+07	2.8E+07	7.2E-01	1.4E+00	2.7E-02
LMRG_02349	lmo0100		1.8E+07	0.0E+00	0.0E+00	#DIV/0!	7.5E-04
LMRG_02350	lmo0101		0.0E+00	5.6E+05	#DIV/0!	0.0E+00	3.7E-01
LMRG_02351	lmo0102		1.3E+06	0.0E+00	0.0E+00	#DIV/0!	3.7E-01
LMRG_02352	lmo0103		1.6E+07	7.3E+06	4.7E-01	2.1E+00	2.9E-02
LMRG_02355	lmo0106		0.0E+00	6.5E+05	#DIV/0!	0.0E+00	3.7E-01
LMRG_02359	lmo0110		3.3E+07	2.6E+06	8.0E-02	1.2E+01	7.8E-04
LMRG_02363	lmo0114		2.9E+06	4.3E+06	1.5E+00	6.8E-01	7.2E-01
LMRG_02379	lmo0130		3.3E+06	3.6E+06	1.1E+00	9.3E-01	9.6E-01
LMRG_02380	lmo0131	PdeB	6.2E+05	0.0E+00	0.0E+00	#DIV/0!	3.7E-01
LMRG_02381	lmo0132		9.2E+07	9.4E+07	1.0E+00	9.8E-01	8.4E-01
LMRG_02383	lmo0134		9.7E+06	1.2E+07	1.3E+00	8.0E-01	2.9E-01
LMRG_02384	lmo0135	CtaP	3.3E+09	2.6E+09	8.1E-01	1.2E+00	2.4E-01
LMRG_02385	lmo0136		0.0E+00	3.8E+06	#DIV/0!	0.0E+00	3.7E-01
LMRG_02386	lmo0137		1.2E+07	1.5E+07	1.2E+00	8.2E-01	3.6E-01
LMRG_02397	lmo0152		5.6E+08	7.7E+08	1.4E+00	7.3E-01	5.0E-02
LMRG_02398	lmo0153	ZinA	2.6E+06	7.0E+06	2.7E+00	3.7E-01	5.3E-02
LMRG_02403	lmo0158	YidA	2.8E+07	1.4E+07	4.8E-01	2.1E+00	1.0E-02
LMRG_02406	lmo0161		5.3E+07	5.5E+07	1.0E+00	9.6E-01	7.5E-01
LMRG_02407	lmo0162	HolB	8.5E+07	9.6E+07	1.1E+00	8.8E-01	9.5E-02
LMRG_02408	lmo0163	YaaT	1.2E+08	8.4E+07	6.7E-01	1.5E+00	2.0E-02
LMRG_02409	lmo0164	YabA	7.8E+07	1.0E+08	1.3E+00	7.5E-01	2.5E-02
LMRG_02410	lmo0165		2.8E+07	3.4E+07	1.2E+00	8.3E-01	1.2E-01
LMRG_02411	lmo0166		0.0E+00	1.2E+06	#DIV/0!	0.0E+00	3.7E-01
LMRG_02412	lmo0167	RsmI	2.2E+07	3.2E+07	1.5E+00	6.8E-01	8.3E-02
LMRG_02413	lmo0168	AbrB	1.5E+06	0.0E+00	0.0E+00	#DIV/0!	3.7E-01
LMRG_02415	lmo0170		5.8E+07	5.5E+07	9.5E-01	1.1E+00	5.1E-01
LMRG_02424	lmo2853		3.8E+08	3.5E+08	9.2E-01	1.1E+00	6.2E-01
LMRG_02425	lmo2854	YidC	1.1E+08	7.7E+07	7.1E-01	1.4E+00	1.3E-01
LMRG_02426	lmo2855	RnpA	6.5E+06	8.5E+06	1.3E+00	7.7E-01	7.3E-01
LMRG_02429	lmo0001	DnaA	2.4E+08	2.8E+08	1.2E+00	8.5E-01	2.4E-02
LMRG_02430	lmo0002	DnaN	6.6E+08	6.4E+08	9.7E-01	1.0E+00	3.7E-01
LMRG_02432	lmo0004		3.8E+07	3.6E+07	9.6E-01	1.0E+00	7.8E-01
LMRG_02433	lmo0005	RecF	9.9E+06	1.0E+07	1.0E+00	9.9E-01	9.5E-01
LMRG_02434	lmo0006	GyrB	4.3E+08	4.1E+08	9.6E-01	1.0E+00	4.2E-01
LMRG_02435	lmo0007	GyrA	5.8E+08	5.3E+08	9.2E-01	1.1E+00	1.9E-01
LMRG_02436	lmo0008	Cls	5.1E+07	3.0E+07	5.8E-01	1.7E+00	3.0E-03
LMRG_02437	lmo0009	SpeG	1.4E+07	1.1E+07	7.7E-01	1.3E+00	8.6E-02
LMRG_02439	lmo0010	Mvk	2.1E+07	2.2E+07	1.0E+00	9.7E-01	7.9E-01
LMRG_02440	lmo0011	Mpd	4.2E+07	4.4E+07	1.1E+00	9.4E-01	1.1E-01
LMRG_02441	lmo0012	Pmk	5.2E+07	2.7E+07	5.3E-01	1.9E+00	2.5E-01
LMRG_02443	lmo0014	QoxB	1.4E+06	0.0E+00	0.0E+00	#DIV/0!	3.7E-01
LMRG_02447	lmo0018	AscB	1.5E+07	1.5E+07	9.9E-01	1.0E+00	8.9E-01
LMRG_02449	lmo0020		2.0E+07	1.6E+07	8.3E-01	1.2E+00	9.6E-02
LMRG_02456	lmo0027		1.2E+07	3.3E+07	2.8E+00	3.5E-01	1.7E-03
LMRG_02459	lmo0030		2.7E+07	3.2E+07	1.2E+00	8.3E-01	2.1E-01
LMRG_02460	lmo0031		4.2E+07	4.6E+07	1.1E+00	9.0E-01	3.8E-01
LMRG_02461	lmo0032		0.0E+00	9.1E+06	#DIV/0!	0.0E+00	3.7E-01
LMRG_02471	lmo0042		5.2E+06	5.2E+06	1.0E+00	9.9E-01	9.9E-01
LMRG_02472	lmo0043	ArcA	0.0E+00	1.1E+07	#DIV/0!	0.0E+00	3.7E-01
LMRG_02473	lmo0044	RpsF	5.9E+09	5.4E+09	9.1E-01	1.1E+00	1.7E-01
LMRG_02474	lmo0045	Ssb	4.8E+08	4.0E+08	8.2E-01	1.2E+00	2.6E-02
LMRG_02475	lmo0046	RpsR	1.7E+09	2.1E+09	1.3E+00	7.9E-01	7.1E-03
LMRG_02476	lmo0047		1.1E+08	1.7E+08	1.5E+00	6.7E-01	3.3E-05
LMRG_02479	lmo0050	AgrC	1.3E+06	0.0E+00	0.0E+00	#DIV/0!	3.7E-01
LMRG_02481	lmo0052	PdeA	8.1E+07	5.4E+07	6.6E-01	1.5E+00	7.8E-04
LMRG_02482	lmo0053	RplI	6.0E+08	3.4E+08	5.6E-01	1.8E+00	1.1E-02
LMRG_02483	lmo0054	DnaC	6.8E+07	5.0E+07	7.4E-01	1.3E+00	4.2E-03
LMRG_02485	lmo0055	PurA	9.6E+08	1.9E+09	2.0E+00	5.0E-01	1.1E-03
LMRG_02486	lmo0056		1.8E+07	3.8E+07	2.2E+00	4.6E-01	6.2E-03
LMRG_02495	lmo1776		3.4E+06	1.9E+06	5.6E-01	1.8E+00	6.0E-01
LMRG_02496	lmo1775	PurE	4.1E+07	1.1E+08	2.7E+00	3.7E-01	1.0E-02
LMRG_02497	lmo1774	PurK	1.3E+08	1.6E+08	1.2E+00	8.1E-01	1.5E-01
LMRG_02498	lmo1773	PurB	2.8E+08	6.2E+08	2.2E+00	4.6E-01	2.9E-03
LMRG_02499	lmo1772	PurC	5.8E+07	1.4E+08	2.4E+00	4.1E-01	1.9E-02
LMRG_02500	lmo1771	PurS	5.0E+07	8.4E+07	1.7E+00	6.0E-01	4.0E-02
LMRG_02501	lmo1770	PurQ	2.9E+07	5.3E+07	1.9E+00	5.4E-01	5.8E-02
LMRG_02502	lmo1769	PurL	7.7E+07	1.7E+08	2.2E+00	4.5E-01	4.9E-03
LMRG_02503	lmo1768	PurF	1.5E+07	3.0E+07	2.0E+00	5.0E-01	2.9E-03
LMRG_02504	lmo1767	PurM	2.9E+07	6.4E+07	2.2E+00	4.5E-01	2.2E-02
LMRG_02505	lmo1766	PurN	3.5E+07	6.8E+07	2.0E+00	5.1E-01	6.1E-02
LMRG_02506	lmo1765	PurH	9.7E+07	1.8E+08	1.8E+00	5.4E-01	2.2E-02
LMRG_02507	lmo1764	PurD	4.8E+07	9.8E+07	2.0E+00	5.0E-01	9.6E-03
LMRG_02508	lmo1763	YerC/YerD	3.3E+07	2.5E+07	7.6E-01	1.3E+00	5.8E-02
LMRG_02511	lmo1760	PcrB	0.0E+00	1.8E+05	#DIV/0!	0.0E+00	3.7E-01
LMRG_02512	lmo1759	PcrA	4.9E+08	7.8E+08	1.6E+00	6.3E-01	1.9E-05
LMRG_02513	lmo1758	LigA	1.4E+08	2.1E+08	1.5E+00	6.6E-01	9.9E-04
LMRG_02514	lmo1757		2.2E+08	3.3E+08	1.5E+00	6.6E-01	7.2E-04
LMRG_02515	lmo1756	GatC	2.3E+08	2.4E+08	1.1E+00	9.5E-01	4.6E-01
LMRG_02516	lmo1755	GatA	1.2E+09	1.2E+09	1.1E+00	9.3E-01	1.3E-01
LMRG_02517	lmo1754	GatB	1.4E+09	1.6E+09	1.1E+00	8.8E-01	9.0E-02
LMRG_02518	lmo1753		1.1E+08	1.0E+08	9.1E-01	1.1E+00	1.8E-01
LMRG_02519	lmo1752		1.5E+07	1.9E+07	1.3E+00	7.6E-01	7.4E-02
LMRG_02521	lmo1750		8.6E+07	7.2E+07	8.3E-01	1.2E+00	4.6E-02

LMRG_02522	lmo1749	AroK	4.7E+07	4.7E+07	1.0E+00	1.0E+00	9.7E-01
LMRG_02523	lmo1748		1.1E+07	1.1E+07	9.6E-01	1.0E+00	8.7E-01
LMRG_02524	lmo1747	VirB	1.8E+07	2.6E+07	1.5E+00	6.6E-01	1.5E-02
LMRG_02525	lmo1746	VirA	0.0E+00	3.3E+06	#DIV/0!	0.0E+00	3.7E-01
LMRG_02526	lmo1745	VirR	5.4E+07	6.0E+07	1.1E+00	9.0E-01	4.9E-02
LMRG_02527	lmo1744		5.9E+07	7.3E+07	1.2E+00	8.1E-01	8.8E-02
LMRG_02528	lmo1743		1.1E+08	1.5E+08	1.4E+00	7.2E-01	2.3E-02
LMRG_02529	lmo1742	Ade	8.4E+07	1.2E+08	1.4E+00	7.2E-01	2.1E-02
LMRG_02530	lmo1741	VirS	1.2E+07	1.7E+07	1.4E+00	7.3E-01	1.3E-01
LMRG_02532	lmo1739		2.5E+06	1.9E+07	7.6E+00	1.3E-01	7.7E-03
LMRG_02533	lmo1738		9.1E+06	2.4E+07	2.6E+00	3.8E-01	2.8E-03
LMRG_02534	lmo1737		8.7E+07	1.0E+08	1.2E+00	8.7E-01	7.1E-02
LMRG_02535	lmo1736		0.0E+00	8.1E+05	#DIV/0!	0.0E+00	3.7E-01
LMRG_02536	lmo1735	GltC	0.0E+00	1.1E+06	#DIV/0!	0.0E+00	3.7E-01
LMRG_02537	lmo1734	GltB	1.4E+07	1.0E+08	7.3E+00	1.4E-01	6.7E-02
LMRG_02538	lmo1733	GltD	0.0E+00	7.7E+06	#DIV/0!	0.0E+00	1.4E-01
LMRG_02544	lmo1727		1.8E+06	1.8E+06	1.0E+00	1.0E+00	1.0E+00
LMRG_02545	lmo1726		8.5E+07	4.8E+07	5.6E-01	1.8E+00	1.8E-02
LMRG_02546	lmo1725		8.7E+05	0.0E+00	0.0E+00	#DIV/0!	3.7E-01
LMRG_02547	lmo1724		0.0E+00	3.5E+06	#DIV/0!	0.0E+00	1.2E-01
LMRG_02549	lmo1722		1.4E+08	1.5E+08	1.1E+00	9.3E-01	2.0E-01
LMRG_02550	lmo1721		1.2E+08	1.1E+08	9.5E-01	1.1E+00	2.7E-01
LMRG_02551	lmo1720		4.0E+07	2.7E+07	6.6E-01	1.5E+00	2.3E-01
LMRG_02552	lmo1719		2.4E+08	2.4E+08	9.7E-01	1.0E+00	8.2E-01
LMRG_02553	lmo1718		2.4E+08	2.4E+08	1.0E+00	1.0E+00	9.8E-01
LMRG_02556	lmo1713	MreB	5.2E+07	7.6E+07	1.5E+00	6.8E-01	2.2E-02
LMRG_02558	lmo1711		6.6E+08	6.4E+08	9.6E-01	1.0E+00	5.2E-01
LMRG_02559	lmo1710		3.8E+08	4.7E+08	1.2E+00	8.1E-01	1.7E-01
LMRG_02561	lmo1709	Map	5.5E+08	5.2E+08	9.4E-01	1.1E+00	3.9E-01
LMRG_02562	lmo1708		1.5E+07	1.4E+07	9.5E-01	1.1E+00	6.0E-01
LMRG_02563	lmo1707		0.0E+00	2.4E+06	#DIV/0!	0.0E+00	3.7E-01
LMRG_02565	lmo0303		2.9E+06	0.0E+00	0.0E+00	#DIV/0!	1.2E-01
LMRG_02566	lmo0302		3.0E+06	0.0E+00	0.0E+00	#DIV/0!	1.2E-01
LMRG_02575			2.3E+06	0.0E+00	0.0E+00	#DIV/0!	3.7E-01
LMRG_02576			2.5E+08	2.0E+08	8.0E-01	1.3E+00	2.4E-03
LMRG_02577			1.5E+07	1.4E+07	9.0E-01	1.1E+00	1.6E-01
LMRG_02578	lmo0293	RlmH	1.3E+07	1.1E+07	8.3E-01	1.2E+00	2.6E-01
LMRG_02579	lmo0292	HtrA	2.3E+08	6.5E+08	2.8E+00	3.6E-01	2.2E-02
LMRG_02580	lmo0291		2.7E+07	2.7E+07	1.0E+00	1.0E+00	9.8E-01
LMRG_02581	lmo0290		1.3E+07	1.3E+07	1.1E+00	9.5E-01	7.0E-01
LMRG_02582	lmo0289	YycH	3.8E+07	4.9E+07	1.3E+00	7.7E-01	2.6E-02
LMRG_02583	lmo0288	WalK	5.6E+07	6.6E+07	1.2E+00	8.4E-01	2.1E-02
LMRG_02584	lmo0287	VicR	4.5E+08	4.6E+08	1.0E+00	9.6E-01	6.3E-01
LMRG_02585	lmo0286		1.4E+07	1.7E+07	1.2E+00	8.4E-01	3.4E-01
LMRG_02586	lmo0285		2.1E+08	2.0E+08	9.1E-01	1.1E+00	3.8E-01
LMRG_02587	lmo0284	MetN	5.0E+07	5.4E+07	1.1E+00	9.2E-01	3.7E-01
LMRG_02589	lmo0282		3.2E+07	8.3E+06	2.6E-01	3.9E+00	4.7E-02
LMRG_02590	lmo0281		7.5E+06	0.0E+00	0.0E+00	#DIV/0!	1.1E-04
LMRG_02595	lmo0280	NrdG	1.3E+07	3.0E+06	2.2E-01	4.5E+00	5.9E-02
LMRG_02596	lmo0279	NrdD	4.9E+08	4.2E+08	8.4E-01	1.2E+00	2.3E-01
LMRG_02597	lmo0278	UgpC	1.5E+08	1.9E+08	1.3E+00	8.0E-01	1.2E-01
LMRG_02598	lmo0277		4.6E+07	4.2E+07	9.0E-01	1.1E+00	3.9E-01
LMRG_02599	lmo0276		4.6E+07	5.3E+07	1.2E+00	8.6E-01	1.7E-01
LMRG_02603	lmo0273		2.7E+08	4.0E+08	1.5E+00	6.7E-01	8.9E-03
LMRG_02604	lmo0272	YidA	1.6E+08	2.8E+08	1.7E+00	5.8E-01	2.3E-04
LMRG_02605	lmo0271		2.4E+08	2.4E+08	9.8E-01	1.0E+00	5.1E-01
LMRG_02607	lmo0269	OppC	0.0E+00	1.4E+06	#DIV/0!	0.0E+00	3.7E-01
LMRG_02608	lmo0268		4.3E+07	5.9E+07	1.4E+00	7.3E-01	5.1E-02
LMRG_02609	lmo0267		2.1E+07	8.9E+06	4.2E-01	2.4E+00	3.0E-02
LMRG_02611	lmo0265		1.9E+07	2.3E+07	1.2E+00	8.4E-01	2.1E-02
LMRG_02614	lmo0192	PurR	7.2E+07	6.4E+07	8.8E-01	1.1E+00	4.7E-01
LMRG_02615	lmo0193		7.1E+07	1.1E+08	1.6E+00	6.4E-01	4.0E-02
LMRG_02616	lmo0194		3.5E+07	4.7E+07	1.3E+00	7.5E-01	1.5E-02
LMRG_02617	lmo0195		2.2E+07	2.5E+07	1.1E+00	8.8E-01	1.2E-01
LMRG_02618	lmo0196	SpoVG1	1.3E+09	1.2E+09	8.9E-01	1.1E+00	3.3E-01
LMRG_02619	lmo0197	SpoVG2	1.0E+09	8.0E+08	7.9E-01	1.3E+00	5.1E-02
LMRG_02620	lmo0198	GlmU	9.2E+08	1.2E+09	1.3E+00	7.8E-01	4.4E-03
LMRG_02621	lmo0199	Prs	1.9E+09	2.1E+09	1.1E+00	8.9E-01	7.3E-02
LMRG_02622	lmo0200	PrfA	1.4E+06	0.0E+00	0.0E+00	#DIV/0!	3.7E-01
LMRG_02629	lmo0207		0.0E+00	3.4E+07	#DIV/0!	0.0E+00	3.7E-01
LMRG_02630	lmo0208		1.1E+08	4.7E+07	4.2E-01	2.4E+00	4.4E-03
LMRG_02631	lmo0209	AdlP	0.0E+00	9.4E+05	#DIV/0!	0.0E+00	3.7E-01
LMRG_02632	lmo0210	Ldh	1.9E+09	3.1E+09	1.6E+00	6.2E-01	1.1E-04
LMRG_02633	lmo0211	RplY	5.9E+08	2.0E+08	3.4E-01	2.9E+00	2.8E-03
LMRG_02635	lmo0213	Pth	8.3E+07	8.8E+07	1.1E+00	9.5E-01	5.1E-01
LMRG_02636	lmo0214	Mfd	2.9E+08	3.1E+08	1.1E+00	9.3E-01	2.3E-01
LMRG_02638	lmo0216		6.4E+07	6.5E+07	1.0E+00	9.8E-01	8.5E-01
LMRG_02640	lmo0218		5.9E+08	6.1E+08	1.0E+00	9.7E-01	5.2E-01
LMRG_02641	lmo0219	TilS	6.6E+08	6.8E+08	1.0E+00	9.6E-01	3.4E-01
LMRG_02642	lmo0220	FtsH	1.4E+09	1.2E+09	8.1E-01	1.2E+00	7.5E-03
LMRG_02643	lmo0221	CoaX	8.2E+06	0.0E+00	0.0E+00	#DIV/0!	1.2E-01
LMRG_02644	lmo0222	HslO	3.1E+08	1.7E+08	5.6E-01	1.8E+00	1.7E-04
LMRG_02645	lmo0223	CysK	3.5E+09	2.0E+09	5.9E-01	1.7E+00	3.8E-03
LMRG_02648	lmo0261		1.9E+07	1.3E+07	7.2E-01	1.4E+00	8.6E-03
LMRG_02649	lmo0260		1.2E+06	0.0E+00	0.0E+00	#DIV/0!	3.7E-01
LMRG_02650	lmo0259	RpoC	6.5E+09	6.4E+09	9.9E-01	1.0E+00	7.4E-01

LMRG_02651	lmo0258	RpoB	6.0E+09	6.4E+09	1.1E+00	9.3E-01	5.6E-02
LMRG_02652	lmo0257		1.6E+07	4.1E+07	2.6E+00	3.8E-01	2.3E-02
LMRG_02653	lmo0256		7.6E+07	1.0E+08	1.3E+00	7.6E-01	5.8E-03
LMRG_02655	lmo0251	RplL	1.8E+10	1.9E+10	1.1E+00	9.4E-01	5.0E-01
LMRG_02656	lmo0250	RplJ	8.5E+09	7.8E+09	9.2E-01	1.1E+00	2.7E-01
LMRG_02657	lmo0249	RplA	5.4E+09	5.6E+09	1.0E+00	9.6E-01	6.3E-01
LMRG_02658	lmo0248	RplK	5.4E+09	4.5E+09	8.3E-01	1.2E+00	5.7E-03
LMRG_02660	lmo0246	NusG	9.3E+08	1.1E+09	1.2E+00	8.5E-01	2.6E-02
LMRG_02661	lmo0245	SecE	0.0E+00	8.3E+06	#DIV/0!	0.0E+00	3.7E-01
LMRG_02662	lmo0244	RpmG	2.8E+07	5.0E+07	1.8E+00	5.6E-01	2.4E-01
LMRG_02663	lmo0243	SigH	2.9E+06	4.0E+06	1.4E+00	7.2E-01	6.8E-01
LMRG_02664	lmo0242		5.7E+05	0.0E+00	0.0E+00	#DIV/0!	3.7E-01
LMRG_02665	lmo0241	RlmB	8.9E+07	7.2E+07	8.0E-01	1.2E+00	2.6E-02
LMRG_02666	lmo0240	MrnC	2.9E+07	2.7E+07	9.5E-01	1.1E+00	4.9E-01
LMRG_02667	lmo0239	CysS	3.2E+08	2.6E+08	7.9E-01	1.3E+00	1.6E-02
LMRG_02669	lmo0237	GltX	1.6E+09	1.5E+09	9.3E-01	1.1E+00	2.5E-01
LMRG_02670	lmo0236	IspF	2.5E+07	3.3E+07	1.3E+00	7.4E-01	4.9E-03
LMRG_02671	lmo0235	IspD	4.6E+06	1.4E+06	3.1E-01	3.2E+00	9.0E-02
LMRG_02672	lmo0234		1.1E+08	9.1E+07	8.3E-01	1.2E+00	2.5E-01
LMRG_02673	lmo0233	RadA	6.5E+07	1.1E+08	1.8E+00	5.7E-01	2.2E-04
LMRG_02674	lmo0232	ClpC	1.4E+09	2.0E+09	1.4E+00	7.1E-01	1.7E-02
LMRG_02675	lmo0231	McsB	5.6E+07	9.6E+07	1.7E+00	5.9E-01	3.7E-02
LMRG_02676	lmo0230		3.3E+07	6.2E+07	1.9E+00	5.3E-01	3.8E-02
LMRG_02677	lmo0229	CtsR	3.8E+07	8.9E+07	2.4E+00	4.2E-01	2.0E-04
LMRG_02678	lmo2589	TetR	1.5E+07	1.5E+07	9.9E-01	1.0E+00	9.6E-01
LMRG_02679	lmo2588	MdrT	4.6E+05	0.0E+00	0.0E+00	#DIV/0!	3.7E-01
LMRG_02684	lmo2583	HssR	2.4E+07	1.1E+07	4.5E-01	2.2E+00	4.6E-01
LMRG_02685	lmo2582	HssS	7.7E+05	0.0E+00	0.0E+00	#DIV/0!	3.7E-01
LMRG_02689	lmo2579		4.8E+07	2.9E+07	5.9E-01	1.7E+00	7.1E-03
LMRG_02690	lmo2578		1.1E+07	7.5E+06	6.8E-01	1.5E+00	3.3E-02
LMRG_02691	lmo2577		1.6E+08	2.2E+08	1.3E+00	7.5E-01	4.6E-02
LMRG_02694	lmo2574		1.3E+07	3.6E+06	2.7E-01	3.8E+00	2.3E-01
LMRG_02696	lmo2572		4.1E+06	4.6E+06	1.1E+00	8.8E-01	6.1E-01
LMRG_02699	lmo2569		3.5E+07	3.5E+07	1.0E+00	1.0E+00	1.0E+00
LMRG_02703	lmo2566	LipL	4.0E+07	4.3E+07	1.1E+00	9.4E-01	7.2E-02
LMRG_02704	lmo2565		5.3E+07	6.2E+07	1.2E+00	8.5E-01	2.4E-01
LMRG_02705	lmo2564		7.4E+07	1.7E+07	2.4E-01	4.2E+00	3.3E-02
LMRG_02707	lmo2562		2.2E+07	1.7E+07	7.9E-01	1.3E+00	2.0E-01
LMRG_02708	lmo2561	ArgS	1.3E+09	1.4E+09	1.1E+00	9.0E-01	3.9E-03
LMRG_02709	lmo2560	RpoE	4.3E+08	7.1E+08	1.6E+00	6.1E-01	2.7E-03
LMRG_02710	lmo2559	PyrG	2.3E+09	2.3E+09	9.8E-01	1.0E+00	6.4E-01
LMRG_02711	lmo2366		9.6E+06	3.4E+06	3.5E-01	2.8E+00	2.8E-02
LMRG_02712	lmo2367	Pgi	5.2E+09	5.2E+09	9.9E-01	1.0E+00	8.6E-01
LMRG_02714	lmo2369	YugI	1.3E+08	8.2E+07	6.3E-01	1.6E+00	2.8E-02
LMRG_02715	lmo2370		1.2E+08	1.3E+08	1.1E+00	9.3E-01	2.3E-01
LMRG_02716	lmo2371		1.2E+08	3.2E+07	2.6E-01	3.8E+00	7.2E-07
LMRG_02717	lmo2372		1.5E+08	2.8E+07	1.9E-01	5.3E+00	5.5E-04
LMRG_02718	lmo2373		3.7E+08	2.1E+08	5.8E-01	1.7E+00	2.2E-03
LMRG_02719	lmo2374		7.7E+06	8.3E+06	1.1E+00	9.2E-01	7.7E-01
LMRG_02721	lmo2376		5.4E+08	3.1E+08	5.6E-01	1.8E+00	4.3E-04
LMRG_02729	lmo2384	MnhG	2.1E+06	0.0E+00	0.0E+00	#DIV/0!	3.7E-01
LMRG_02730	lmo2385	MenI	6.0E+07	8.1E+07	1.4E+00	7.3E-01	2.4E-01
LMRG_02731	lmo2386		1.2E+07	1.2E+07	9.9E-01	1.0E+00	9.5E-01
LMRG_02732	lmo2387		1.7E+06	0.0E+00	0.0E+00	#DIV/0!	3.7E-01
LMRG_02733	lmo2388		2.9E+06	6.0E+06	2.1E+00	4.8E-01	5.1E-01
LMRG_02734	lmo2389		1.5E+08	5.2E+07	3.5E-01	2.8E+00	1.3E-03
LMRG_02735	lmo2390		3.9E+07	3.2E+06	8.1E-02	1.2E+01	1.1E-03
LMRG_02736	lmo2391		3.7E+07	3.0E+07	8.1E-01	1.2E+00	1.4E-01
LMRG_02742	lmo0177	MetG	1.1E+09	1.4E+09	1.3E+00	7.7E-01	4.1E-05
LMRG_02743	lmo0178		4.1E+07	4.2E+07	1.0E+00	9.6E-01	7.0E-01
LMRG_02746	lmo0181		1.4E+08	1.7E+08	1.2E+00	8.3E-01	2.7E-01
LMRG_02747	lmo0182		4.1E+07	1.1E+08	2.6E+00	3.8E-01	1.9E-02
LMRG_02748	lmo0183		4.0E+07	9.2E+07	2.3E+00	4.4E-01	3.2E-02
LMRG_02750	lmo0185	YcfH	8.8E+07	6.2E+07	7.0E-01	1.4E+00	2.1E-03
LMRG_02751	lmo0186		2.8E+07	2.0E+07	7.1E-01	1.4E+00	3.4E-01
LMRG_02752	lmo0187	RnmV	2.6E+07	2.8E+07	1.1E+00	9.3E-01	6.0E-01
LMRG_02753	lmo0188	RsmA	1.2E+08	1.7E+08	1.4E+00	7.1E-01	5.2E-03
LMRG_02754	lmo0189		0.0E+00	1.5E+06	#DIV/0!	0.0E+00	3.7E-01
LMRG_02755	lmo0190	IspE	2.1E+07	2.5E+07	1.2E+00	8.3E-01	1.7E-01
LMRG_02757	lmo1683	PerR	5.2E+07	5.4E+07	1.0E+00	9.7E-01	6.9E-01
LMRG_02759	lmo1685	HemL	2.2E+08	3.2E+08	1.4E+00	6.9E-01	2.7E-03
LMRG_02760	lmo1686		0.0E+00	2.4E+06	#DIV/0!	0.0E+00	3.7E-01
LMRG_02761	lmo1687		2.2E+07	7.7E+06	3.5E-01	2.8E+00	6.0E-02
LMRG_02762	lmo1688	FabL	2.6E+07	5.6E+06	2.1E-01	4.7E+00	2.7E-02
LMRG_02764	lmo1690		0.0E+00	2.4E+05	#DIV/0!	0.0E+00	3.7E-01
LMRG_02765	lmo1691		7.1E+07	6.2E+07	8.7E-01	1.1E+00	1.6E-01
LMRG_02766	lmo1692		2.1E+07	1.9E+07	8.8E-01	1.1E+00	4.3E-01
LMRG_02768	lmo1694		5.8E+06	1.3E+07	2.2E+00	4.5E-01	7.0E-02
LMRG_02769	lmo1695	MprF	1.1E+08	1.1E+08	9.8E-01	1.0E+00	8.7E-01
LMRG_02771	lmo1697		5.6E+06	1.7E+06	3.0E-01	3.3E+00	9.0E-02
LMRG_02773	lmo1699		2.3E+08	4.0E+07	1.7E-01	5.9E+00	3.3E-03
LMRG_02774	lmo1700		6.0E+06	0.0E+00	0.0E+00	#DIV/0!	1.4E-01
LMRG_02775	lmo1701		4.1E+07	4.8E+07	1.2E+00	8.5E-01	2.4E-01
LMRG_02776	lmo1702	FosX	0.0E+00	5.1E+05	#DIV/0!	0.0E+00	3.7E-01
LMRG_02777	lmo1703	RumA	1.2E+08	1.2E+08	1.0E+00	9.7E-01	7.0E-01
LMRG_02779	lmo1705		8.7E+06	1.5E+07	1.8E+00	5.6E-01	2.0E-01

LMRG_02782	lmo0815		2.5E+06	0.0E+00	0.0E+00	#DIV/0!	1.2E-01
LMRG_02783	lmo0814	FabK1	8.1E+08	1.1E+09	1.4E+00	7.1E-01	7.0E-04
LMRG_02784	lmo0813		9.3E+07	1.0E+08	1.1E+00	9.1E-01	4.2E-01
LMRG_02786	lmo0811		4.8E+07	0.0E+00	0.0E+00	#DIV/0!	5.6E-06
LMRG_02787	lmo0810		1.1E+07	1.1E+07	1.0E+00	9.5E-01	5.1E-01
LMRG_02790	lmo0807	PotA	5.1E+07	5.1E+07	1.0E+00	1.0E+00	9.8E-01
LMRG_02791	lmo0806		1.4E+07	1.7E+07	1.2E+00	8.1E-01	1.5E-01
LMRG_02793	lmo0804		0.0E+00	3.1E+05	#DIV/0!	0.0E+00	3.7E-01
LMRG_02794	lmo0803		8.0E+06	6.4E+06	8.0E-01	1.2E+00	2.5E-01
LMRG_02795	lmo0802		1.1E+07	2.3E+07	2.2E+00	4.5E-01	2.1E-02
LMRG_02797	lmo2143		0.0E+00	1.4E+07	#DIV/0!	0.0E+00	2.2E-02
LMRG_02798	lmo2142		9.0E+06	2.1E+07	2.3E+00	4.4E-01	6.6E-02
LMRG_02800	lmo2140	LstB	8.0E+05	0.0E+00	0.0E+00	#DIV/0!	3.7E-01
LMRG_02801	lmo2139	LstA	1.2E+07	0.0E+00	0.0E+00	#DIV/0!	1.1E-04
LMRG_02811	lmo1787	RplS	4.9E+09	5.2E+09	1.1E+00	9.4E-01	1.1E-01
LMRG_02815	lmo1791		3.0E+07	2.7E+07	8.8E-01	1.1E+00	2.2E-01
LMRG_02816	lmo1792	TrmD	3.2E+07	3.2E+07	9.9E-01	1.0E+00	9.4E-01
LMRG_02817	lmo1793	RimM	1.3E+08	1.2E+08	9.6E-01	1.0E+00	4.6E-01
LMRG_02819	lmo1795		1.2E+07	4.8E+06	4.0E-01	2.5E+00	4.9E-02
LMRG_02820	lmo1796		1.4E+08	9.9E+07	7.2E-01	1.4E+00	9.4E-02
LMRG_02821	lmo1797	RpsP	2.3E+09	2.0E+09	8.9E-01	1.1E+00	3.5E-01
LMRG_02827	lmo1785	InfC	1.8E+09	1.6E+09	8.6E-01	1.2E+00	4.3E-02
LMRG_02828	lmo1784	RpmI	7.4E+08	1.3E+09	1.7E+00	5.9E-01	5.5E-04
LMRG_02829	lmo1783	RplT	2.0E+09	2.5E+09	1.3E+00	7.9E-01	8.9E-02
LMRG_02830	lmo1782	Xth	1.7E+07	6.0E+06	3.5E-01	2.8E+00	5.8E-02
LMRG_02832	lmo1780	PepT	2.8E+08	1.0E+08	3.7E-01	2.7E+00	2.1E-04
LMRG_02836	lmo0228	LysS	1.3E+09	1.9E+09	1.4E+00	7.0E-01	8.9E-05
LMRG_02837	lmo0227	DusB	6.7E+07	4.8E+07	7.1E-01	1.4E+00	1.9E-02
LMRG_02838	lmo0226	FolK	4.6E+06	3.7E+06	8.1E-01	1.2E+00	7.8E-01
LMRG_02839	lmo0225	FolA	4.2E+07	3.5E+07	8.3E-01	1.2E+00	2.6E-01
LMRG_02843	lmo0900		6.0E+07	2.6E+07	4.4E-01	2.3E+00	3.0E-03
LMRG_02844	lmo0901	CelB	1.5E+06	0.0E+00	0.0E+00	#DIV/0!	3.7E-01
LMRG_02845	lmo0902		7.7E+06	1.4E+06	1.9E-01	5.4E+00	2.0E-01
LMRG_02856	lmo1297		1.7E+07	1.8E+07	1.1E+00	9.4E-01	3.0E-01
LMRG_02869	lmo0783		1.6E+08	1.6E+08	9.9E-01	1.0E+00	9.6E-01
LMRG_02874	lmo0675	FliN	1.3E+07	5.9E+06	4.6E-01	2.2E+00	1.4E-01
LMRG_02878	lmo0642		0.0E+00	1.5E+06	#DIV/0!	0.0E+00	3.7E-01
LMRG_02879	lmo0528	PssB	5.7E+06	1.8E+06	3.2E-01	3.1E+00	1.0E-01
LMRG_02891			1.2E+07	6.7E+06	5.8E-01	1.7E+00	2.4E-01
LMRG_02900	lmo2773		1.4E+06	0.0E+00	0.0E+00	#DIV/0!	3.7E-01
LMRG_02904	lmo2652		1.3E+07	2.6E+07	2.0E+00	4.9E-01	5.1E-01
LMRG_02906	lmo2610	InfA	3.4E+08	4.3E+08	1.3E+00	8.0E-01	3.5E-01
LMRG_02909	lmo2558	Ami	1.1E+09	1.3E+09	1.2E+00	8.3E-01	1.0E-01
LMRG_02917	lmo2364	RofA	4.0E+06	2.4E+06	6.0E-01	1.7E+00	2.8E-01
LMRG_02922	lmo2267	AddA	2.8E+07	3.4E+07	1.2E+00	8.3E-01	8.5E-02
LMRG_02924	lmo2222		6.9E+06	1.5E+07	2.2E+00	4.6E-01	1.2E-02
LMRG_02938	lmo0238	CysE	2.4E+06	0.0E+00	0.0E+00	#DIV/0!	3.7E-01
LMRG_02940	lmo1557	HemA	3.6E+07	3.6E+07	9.9E-01	1.0E+00	8.4E-01
LMRG_02943	lmo1574	DnaE	4.5E+07	6.1E+07	1.4E+00	7.4E-01	3.4E-02
LMRG_02949	lmo0945	ComEC	1.2E+07	1.2E+07	1.0E+00	1.0E+00	9.8E-01
LMRG_02950	lmo0940		1.8E+08	2.9E+08	1.6E+00	6.1E-01	7.3E-03
LMRG_02958	lmo1706		3.8E+05	0.0E+00	0.0E+00	#DIV/0!	3.7E-01
LMRG_02963	lmo0191	ChbG	1.7E+08	1.5E+08	9.0E-01	1.1E+00	2.6E-01
LMRG_02970	lmo0184		1.1E+08	1.8E+08	1.7E+00	6.0E-01	5.9E-02
LMRG_02971	lmo0386	IolD	5.0E+05	0.0E+00	0.0E+00	#DIV/0!	3.7E-01
LMRG_02973	lmo1036		0.0E+00	9.8E+06	#DIV/0!	0.0E+00	5.3E-06
LMRG_02974	lmo1070		0.0E+00	1.5E+06	#DIV/0!	0.0E+00	3.7E-01
LMRG_02975	lmo1296	HflX	2.6E+06	0.0E+00	0.0E+00	#DIV/0!	3.7E-01
LMRG_02977	lmo1751	RlmD	6.9E+07	5.5E+07	8.0E-01	1.3E+00	6.4E-02
LMRG_02979	lmo1859	MsrB	2.7E+07	2.1E+07	7.9E-01	1.3E+00	8.0E-02
LMRG_02980	lmo1978	Zwf	5.9E+08	5.3E+08	9.0E-01	1.1E+00	7.5E-02
LMRG_02985	lmo2824		4.2E+07	2.2E+07	5.3E-01	1.9E+00	2.8E-04

Table S4. Strains used in this study

Strain	Description	Reference or Source
E. coli		
MLR-E0472	*pPL2.spxA1*	(1)
MLR-E0558	*pPL2.mCherry*	(2)
MLR-E0559	*pPL2.GFP*	(3)
MLR-E0893	*pLIM*	Gift from Arne Rietsch (Case Western Reserve University)
MLR-E1078	*pLIMΔspxA1*	This work
MLR-E1079	*pLIMΔmecA*	This work
MLR-E1080	*pLIMΔclpE*	This work
MLR-E1082	*pKSV7ΔflaA*	This work
L. monocytogenes		
MLR-L0001	10403S	(4)
MLR-L0081	Δ*yjbH*	(5)
MLR-L0408	*yjbH::Tn*	(5)
MLR-L0424	*gtcA::Tn*	(5)
MLR-L0448	*clpC::Tn*	(6)
MLR-L0449	*clpX::Tn*	(6)
MLR-L0470	Δ*spxA2*	(7)
MLR-L0551	*pPL2.mCherry*	(2)
MLR-L0552	*pPL2.GFP*	(3)
MLR-L0752	Δ*rex*	(8)
MLR-L0828	Δ*kat* Δ*lmo2784 pPL2.lmo2784*	(1)
MLR-L0888	Δ*hemEH*	(1)
MLR-L0944	Δ*lmo2113*	(9)
MLR-L0953	Δ*lmo1609*	(9)
MLR-L0954	Δ*lmo0983*	(9)
MLR-L0959	Δ*tpx*	(9)
MLR-L1066	Δ*spxA1*	This work
MLR-L1067	Δ*spxA1 pPL2.spxA1*	This work
MLR-L1068	Δ*flaA*	This work
MLR-L1069	Δ*clpE*	This work
MLR-L1070	Δ*mecA*	This work
MLR-L1072	Δ*spxA1* Δ*mecA*	This work
MLR-L1073	Δ*spxA1* Δ*clpE*	This work
MLR-L1074	Δ*spxA1 clpC::Tn*	This work
MLR-L1075	Δ*spxA1 clpX::Tn*	This work
MLR-L1076	Δ*spxA1 yjbH::Tn*	This work
MLR-L1083	Δ*spxA1 pPL2.mCherry*	This work
MLR-L1094	Δ*spxA1* Δ*spxA2*	This work

References for Table S4

1. Cesinger MR, Thomason MK, Edrozo MB, Halsey CR, Reniere ML. 2020. *Listeria monocytogenes* SpxA1 is a global regulator required to activate genes encoding catalase and heme biosynthesis enzymes for aerobic growth. *Mol Microbiol* 114:230–243.
2. Vincent WJB, Freisinger CM, Lam P, Huttenlocher A, Sauer J-D. 2016. Macrophages mediate flagellin induced inflammasome activation and host defense in zebrafish. *Cell Microbiol* 18:591–604.
3. Chen C, Nguyen BN, Mitchell G, Margolis SR, Ma D, Portnoy DA. 2018. The Listeriolysin O PEST-like Sequence Co-opts AP-2-Mediated Endocytosis to Prevent Plasma Membrane Damage during *Listeria* Infection. *Cell Host Microbe* 23:786-795.e5.
4. Bécavin C, Bouchier C, Lechat P, Archambaud C, Creno S, Gouin E, Wu Z, Kühbacher A, Brisse S, Pucciarelli MG, García-Del Portillo F, Hain T, Portnoy DA, Chakraborty T, Lecuit M, Pizarro-Cerda J, Moszer I, Bierne H, Cossart P. 2014. Comparison of Widely Used *Listeria monocytogenes* Strains EGD, 10403S, and EGD-e Highlights Genomic Variations Underlying Differences in Pathogenicity. *mBio* 5:e00969-14.
5. Reniere ML, Whiteley AT, Portnoy DA. 2016. An *In Vivo* Selection Identifies *Listeria monocytogenes* Genes Required to Sense the Intracellular Environment and Activate Virulence Factor Expression. *PLoS Pathog* 12:e1005741.
6. Zemansky J, Kline BC, Woodward JJ, Leber JH, Marquis H, Portnoy DA. 2009. Development of a mariner-based transposon and identification of *Listeria monocytogenes* determinants, including the peptidyl-prolyl isomerase PrsA2, that contribute to its hemolytic phenotype. *J Bacteriol* 191:3950–3964.
7. Whiteley AT, Ruhland BR, Edrozo MB, Reniere ML. 2017. A Redox-Responsive Transcription Factor Is Critical for Pathogenesis and Aerobic Growth of *Listeria monocytogenes. Infect Immun* 85:e00978-16.
8. Halsey CR, Glover RC, Thomason MK, Reniere ML. 2021. The redox-responsive transcriptional regulator Rex represses fermentative metabolism and is required for *Listeria monocytogenes* pathogenesis. *PLoS Pathog* 17:e1009379.
9. Cesinger MR, Schwardt NH, Halsey CR, Thomason MK, Reniere ML. 2021. Investigating the Roles of *Listeria monocytogenes* Peroxidases in Growth and Virulence. *Microbiol Spectr* 9:e00440-21.
